# dTMP imbalance through thymidylate 5′-phosphohydrolase activity induces apoptosis in triple-negative breast cancers

**DOI:** 10.1038/s41598-022-24706-4

**Published:** 2022-11-21

**Authors:** Dae-Ho Kim, Jin-Sook Kim, Chang-Soo Mok, En-Hyung Chang, Jiwon Choi, Junsub Lim, Chul-Ho Kim, Ah-Reum Park, Yu-Jeong Bae, Bong-Seong Koo, Hyeon-Cheol Lee

**Affiliations:** 1 Research Center, BPgene Co, Ltd, Seoul, 03127 Republic of Korea; 2 Department of Molecular Science and Technology, Ajou University, Suwon, 16499 Republic of Korea; 3 Department of Otolaryngology, Ajou University School of Medicine, Suwon, 16499 Republic of Korea; 4 Department of Life Science, Dongguk University Biomedi Campus, Gyeonggi-do, 10326 Republic of Korea; 5 ForBioKorea Co., Ltd, Seoul, Republic of Korea

**Keywords:** Biochemistry, Cancer, Drug discovery

## Abstract

Immunotherapy has a number of advantages over traditional anti-tumor therapy but can cause severe adverse reactions due to an overactive immune system. In contrast, a novel metabolic treatment approach can induce metabolic vulnerability through multiple cancer cell targets. Here, we show a therapeutic effect by inducing nucleotide imbalance and apoptosis in triple negative breast cancer cells (TNBC), by treating with cytosolic thymidylate 5'-phosphohydrolase (CT). We show that a sustained consumption of dTMP by CT could induce dNTP imbalance, leading to apoptosis as tricarboxylic acid cycle intermediates were depleted to mitigate this imbalance. These cytotoxic effects appeared to be different, depending on substrate specificity of the 5′ nucleotide or metabolic dependency of the cancer cell lines. Using representative TNBC cell lines, we reveal how the TNBC cells were affected by CT-transfection through extracellular acidification rate (ECAR)/oxygen consumption rate (OCR) analysis and differential transcription/expression levels. We suggest a novel approach for treating refractory TNBC by an mRNA drug that can exploit metabolic dependencies to exacerbate cell metabolic vulnerability.

## Introduction

Conventional anti-cancer therapy is often limited by chemoresistance, radiation resistance, and severe side effects like cardiotoxicity or neurocognitive deficits. Accordingly, there is great need for innovative cancer therapeutics. Triple-negative breast cancer (TNBC) comprises 15–20% of breast cancers and as an aggressive malignancy that is not susceptible to hormone inhibition must be treated with conventional chemotherapy^1^. Even though neoadjuvant chemotherapy is partially helpful, TNBC treatment still remains a challenge as patients inevitably develop resistance, making it difficult to fully eradicate their tumors. Immunotherapy has led to a paradigm shift in treatment options and sparked an abundance of research with immuno-oncology drugs in new combinations and indications. Recently, the FDA granted an accelerated approval to the combination of atezolizumab (Tecentriq) and nab-paclitaxel (Abraxane) for the frontline treatment of patients with unresectable locally advanced or metastatic PD-L1-positive TNBC^2^. Successes continue to be based on findings of useful immunological targets such as PD-L1, AKT and PARP. While immunotherapy is being evaluated with neoadjuvants to accelerate a more robust immune response, improvement of the response rates for immunotherapies remains a great challenge for cancer treatment in general and for breast cancer in particular due to overactive immune responses, complexity, and uncertainties.

Many researchers have tried to distinguish cancer cells from normal cells by differences in cellular metabolism related to cancer cell growth and proliferation^3–7^. Renewed interests in understanding the mechanisms and consequences of altered tumor metabolism were driven when Nobel Prize winning biochemist Otto Warburg first described glucose-dependent growth in cancer cells^8,9^. Metabolic analysis showed that cancer cells overuse TCA metabolites rather than oxidative phosphorylation as the source of building blocks for early stage cancer cell growth, resulting in glucose-dependent growth known as the Warburg effect. To reduce the resulting surplus NADH and recover the TCA cycle, cancer cells increase the demand for glutamine, if lactate production is at a maximum. The surplus ammonia generated is incorporated into pyrimidine metabolism to minimize damage to cancer cells, releasing the orotate intermediate of the pyrimidine biosynthetic pathway. The released orotate is then converted to urea in the blood to reduce toxicity^10,11^. These metabolic features were considered a direct consequence of perturbations in signaling cascades triggered by mutations or changes of expression in tumor suppressors and oncogenes^12,13^. These findings suggested that mitochondrial oxidative phosphorylation and cancer glycolytic phenotypes strongly associate with de novo nucleotide synthesis, enabling rapid cellular growth under the selective pressure of a relatively hypoxic microenvironment. Additionally, many metabolic genes co-amplified with oncogenes may support oncogenic drivers by reprogramming metabolic pathways to confer survival and growth advantages, positively selected during tumor development^14^.

Unlike normal cells, TNBC cells can gain metabolic flexibility by remodeling their metabolic pathways to use alternative nutrient sources from the environment for cell survival and proliferation. However, this burdensome metabolic reprogramming may introduce a vulnerability in TNBC cells by restricting metabolite availability. Under these conditions, TNBC cells need to balance metabolite availability with the energetic cost of synthesis^6,9^. These metabolic differences, referred to as metabolic vulnerability, between cancerous and normal cells are broadly found in TNBC, which exhibit the inevitable dependence on the mitochondrial TCA cycle, with slight differences observed in TNBC subtypes. It was also strongly suggested that tumors with a certain metabolic deficiency may have higher dependency on the complementary metabolism gene or pathway, thus creating a rational targeting strategy^15^. Most cancer cells including TNBC likely employ different mechanisms not only to activate de novo and salvage pathways but also to desensitize feedback regulatory pathways to circumvent allosteric inhibition, ensuring a continuous flow of cellular nitrogen toward pathways for cell proliferation^10–13,16^. Thus, interrupting a continuous flow of the cellular nitrogen required for dNTP biosynthesis could severely shock cancer cells, with resulting damages varying depending on metabolic vulnerabilities^9^. Since pyrimidine biosynthesis has been closely linked to the metabolic shift for proliferation of cancerous cells, we focused on the highly specific pyrimidine nucleotide-hydrolyzing enzyme, 5′ nucleotidase, as a potential drug candidate to exploit and increase the metabolic vulnerability of TNBC cells^17,18^. Here, we proposed an innovative approach where metabolically vulnerable TNBC cells could be forced to reprogram their metabolic pathway by expressing a cytosolic thymidylate 5'-phosphohydrolase (CT), which has higher specificity for dTMP than any other dNMP. Metabolic effects of a phage-derived CT (T001) on TNBC cells were investigated. The expression of CT T001 induced a nucleotide imbalance due to dTMP overconsumption, increasing the metabolic vulnerability of the TNBC cells.

As discussed above, treating TNBC by targeting oncogenes is challenging since oncogenic signaling can trigger reprogramming of metabolic pathways, altering metabolic gene expression. The novel approach presented here targeting universal metabolic vulnerability in TNBC cells therefore could be a potentially effective alternative, either alone or together with specific immunological therapy. We demonstrate that treating refractory TNBC by a CT mRNA drug can exacerbate metabolic vulnerability derived from dependencies on glycolysis and oxidative phosphorylation. This metabolic targeting approach could open a new therapeutic window that is not obvious in the conventional oncology pipeline.

## Methods

### Cells and media

Human triple-negative breast cancer cell lines (MDA-MB-231, BT-549, MDA-MB-468) were purchased from the American Type Culture Collection (ATCC). To better reflect physiological conditions, cells were cultured in Plasmax™ cell culture medium (Ximbio) supplemented with 2.5% dialyzed FBS (Gibco) and 1X Penicillin–Streptomycin (Gibco). Cells were maintained at 37 °C under 5% CO_2_ in a humidified incubator.

### Optimization of mRNA structure

Since the translational efficiency of mRNA is tightly regulated to ensure a proper amount of protein in cells, it is important in functional activity to choose appropriate 5′UTR together with 3′UTR, which contains AU-rich elements, polyadenylation signals, cis and trans regulatory elements. To evaluate the effect of UTRs on translational efficiency of mRNA, we generated 10 different GFP-encoding in vitro transcription (IVT) templates. All template plasmids for IVT in this study were based on pIRES vector. To prepare the UTR screening plasmid, pIRES was linearized by NheI/NotI digestion and three DNA fragments, 5′UTR and 3′UTR and the reporter gene (GFP), were amplified by PCR for Gibson assembly. After Gibson assembly was completed, each sequence of the constructed plasmids was confirmed by sequencing. The UTRs used in this study are described in Supp. Fig. S1A. To assess how different mRNA-structures affect the onset, duration and level of gene expression, we transfected MCF7 cell line with 10 different GFP mRNAs synthesized from all IVT templates and analyzed GFP expression by flow cytometry (Supp. Fig. S1B). We observed that the 9th set of GFP mRNA showed higher expression with extended durability than other sets. Using this optimized platform for mRNA expression, we prepared mRNA for the following experiments from each plasmid DNA template by replacing the GFP gene with the genes of interest. The CT gene used in this study was synthesized with codon optimization for expression in human cell lines by Integrated DNA Technologies, Inc. For comparison of substrate preferences, human 5′-nucleotidase (CdN) and signal sequence-deleted mitochondrial 5′-nucleotidase (NT5M) were also prepared by PCR amplification from human cDNA library.

### Preparation of CT mRNA

To prepare template DNA for IVT, each DNA template including the transcription cassette was amplified by PCR. The amplicons were purified using a PCR purification kit (Qiagen) before being used as templates for IVT. In vitro transcribed mRNAs were prepared using a HiScribe T7 ARCA mRNA kit (with tailing) (NEB) with 0.5–1 μg template DNA and 1.25 mM of 5-methyl-CTP (TriLink Biotechnologies). Reaction conditions and mRNA purification procedures followed the NEB manufacturer’s instructions without modification.

### In vitro transcribed mRNA transfection

Cells were plated for 24 h before transfection in a 6-well plate at 5 × 10^5^ cells/well. For transfection mixture preparation, appropriate amounts of mRNA complexed with Lipofectamine MessengerMAX (Invitrogen) were diluted in Opti-MEM medium (Gibco) to 200 µl total volume. The transfection mixture was added to the cells, and cells were incubated at 37 °C in 5% CO_2_ for time periods depending on the experiment. Transfection efficiency was evaluated by uptake of GFP mRNA as measured by determining GFP fluorescence using flow cytometry (Beckman).

### dNTPs extraction

According to Wilson et al., we followed a published protocol with slight modification^19^. Cells were plated before transfection in 6-well plates at 5 × 10^5^ cells/well and allowed to adhere and enter log-phase growth for 24 h. Cells were transfected with 1.25 µg of mRNA and then incubated for 6 h. Cells were washed twice with PBS and detached using 0.25% trypsin–EDTA (Gibco). The counted cell samples (3 × 10^6^ cells) were centrifuged at 3000 rpm for 5 min at 4 °C and resuspended in 500 µl of 60% ice-cold methanol by vortexing. To quench residual enzymatic activity and facilitate extraction, samples were incubated for 3 min at 95 °C. The samples were centrifuged at 18,500*g* for 6 min at 4 °C to remove cell debris. The supernatants were passed through a pre-equilibrated Amicon Ultra-0.5-ml centrifugal filter Unit (Merck) to remove macromolecules according to the manufacturer’s instruction. Then, methanol and hydrophobic metabolites were removed by washing twice with 1.4 ml diethyl ether. Residual diethyl ether was removed using a Speed vacuum concentrator (Biotron) for 15 min and the resultant pellet was resuspended in ~ 100 µl nuclease-free water and assayed immediately or stored at − 80 °C until use.

### Q5 DNA polymerase and EvaGreen-based assay for dNTPs using 197-nt templates

A published protocol was followed without modifications^20^. For 2 × master mix, the final concentrations of components were the following: 2 × Q5 reaction buffer, 0.5 µM primer and 0.5 µM 197-nt template, 0.1 mM dNTPs (except for target dNTP) and 2.5 µM EvaGreen (Biotum). Q5 DNA polymerase (NEB) was then added to reaction mixtures as follows: final concentration of 20 U/ml for dATP and dTTP, and 10 U/ml for dGTP and dCTP detection. Five µl of each 2 × master mix was pipetted into Hard-Shell® 96-Well PCR Plates (Bio-Rad) according to dNTP species. Then 5 µl of samples was added to each well for dNTP quantification by real-time PCR. For initial optimization runs, the thermal cycler (CFX96, Bio-Rad) was programmed to heat the plate to 98 °C (10 s) followed by cooling to the final reaction temperature (67 °C). The baseline fluorescence was immediately read after reaching the target temperature. Thereafter, the fluorescence (SYBR Green/FAM channel of the instrument) was recorded typically once every 5 min for total of 75 min. Data were normalized to the volume of estimated samples.

### Cell viability assay

Cells were plated for 24 h before transfection in 6-well plates at 5 × 10^5^ cells/well and transfected with 1.25 µg of mRNA for 24 h. After incubation, cells were treated with EZ-Cytox (DoGen) and incubated for 1 h at 37 °C, 5% CO_2_. The amount of formazan dye formed as a result of the cleavage of the tetrazolium salt WST-1 was measured at 450 nm using a Multiskan Microplate Reader (Thermo Fisher Scientific).

### NADPH/NADP determination

MDA-MB-231 cells were seeded in 6-well plates at 5 × 10^5^ cells/well. MDA-MB-231 cells were transfected with 1.25 µg of CT mRNA and incubated for 6 h. 4 × 10^6^ total cells were harvested for assay. Intracellular NADPH levels were measured using the NADP/NADPH assay kit (Abcam, ab65349). Following the manufacturer’s protocol without modification, NADPH and NADP concentrations were determined based on absorbance at 450 nm. Concentrations of the samples were calculated according to standard curves simultaneously generated from a gradient of concentrations of NADP and NADPH.

### Apoptosis assay

Cells were seeded in 6-well plates at 5 × 10^5^ cells/well and transfected with 0.625, 1.25, or 2.5 µg of mRNA after incubating for 24 h. After treating with 0.25% trypsin–EDTA (Gibco), cells were harvested by centrifugation at 3000 rpm for 3 min, and the pellets were resuspended in PBS. According to the manufacturer’s protocol, cells were stained with FITC annexin V Apoptosis Detection Kit (BD biosciences) and 10,000 cells were read from each sample by detecting relative fluorescence using a flow cytometer (Beckman). Using CytExpert 2.4 software (Beckman), annexin V-FITC positive/PI-PE negative cells and annexin V-FITC/PI-PE double-positive cells were considered to be in the early and late stages of apoptosis, respectively.

### Western blotting

Cells were plated in 6-well plates at 5 × 10^5^ cells/well for 24 h before transfection. Cells were transfected with 1.25 µg of mRNA and incubated for time intervals specified for each experiment. After transfection, cell lysates were extracted using RIPA buffer (Sigma) and protein concentration was determined by a DC protein assay (Bio-Rad). Samples were boiled for 5 min and 10 μg of protein were separated by SDS-PAGE, transferred to PVDF membrane using eBlot™ L1 Protein Transfer System (Genscript), blocked with 5% BSA (Sigma) prepared in 0.1% TBS-Tween20 and probed with the relevant primary antibody overnight at 4 °C. Membranes were then incubated with horseradish-peroxide-conjugated secondary antibodies at room temperature, and proteins were detected using Amersham ECL Select Western Blotting Detection Reagent (Cytiva). The blots were cut prior to hybridization with antibodies. Original blots are presented in Supplementary Information (Figure Raw data). Blots were imaged using ChemiDoc XRS+ System (Bio-Rad).

Primary antibodies were purchased as follows: p-CAD(S1859) (Cell signaling technology, 12662), p-CPS2(T456) (Santa cruz biotechnology, sc-377559), CPS2 (Santa cruz biotechnology, sc-376072), TYMS (Cell signaling technology, 9045), TK1 (Cell signaling technology, 28755), p-p70S6K(T389) (Cell signaling technology, 9205), p70S6K (Cell signaling technology, 2708), p-p44/42 MAPK(ERK1/2)(T202/Y204) (Cell signaling technology, 4370), p44/42 MAPK(ERK1/2) (Cell signaling technology, 4695), RRM1 (Cell signaling technology, 8637), RRM2 (Cell signaling technology, 65939), SAMD1 (Cell signaling technology, 49158), c-Myc (Cell signaling technology, 18583), Glutaminase-1 (Cell signaling technology, 49363), Glutamate dehydrogenase 1/2 (Cell signaling technology, 12793), Hexokinase I (Cell signaling technology, 2024), Hexokinase II (Cell signaling technology, 2867), PFKP (Cell signaling technology, 8164), PKM 1/2 (Cell signaling technology, 3190), Pyruvate dehydrogenase (Cell signaling technology, 3205), LDHA (Cell signaling technology, 3582), SDHA (Cell signaling technology, 11998), Fumarase (Cell signaling technology, 4567), Citrate Synthase (Cell signaling technology, 14309), ACO2 (Cell signaling technology, 6571), IDH2 (Cell signaling technology, 56439), OGDH (Cell signaling technology, 26865), p53 (Cell signaling technology, 2527), p-Histon H2A.X(S139) (Cell signaling technology, 2577), PARP (Cell signaling technology, 9542), Myc-tag (Cell signaling technology, 2272), and GAPDH (Cell signaling technology, 2118). Secondary antibodies were purchased as follows: Goat Anti-Mouse IgG H&L (HRP) (Abcam, ab6789), and Goat Anti-Rabbit IgG H&L (HRP) (Abcam, ab97051).

### ECAR/OCR analysis

XF sensor cartridges were hydrated before each assay. After 200 µl of XF calibrant was added, the XF sensor cartridges were incubated overnight at 37 °C in a CO_2_-free incubator. Cells were plated in XF cell culture microplates at 4 × 10^4^ cells/well and were incubated at 37 °C under 5% CO_2_ in a humidified incubator, overnight. Cells were transfected with 50 ng of mRNA and incubated for 6 h. During the assay, cells were incubated in XF media with 5.5 mM glucose, 0.65 mM glutamine, and 0.1 mM pyruvate. Basal and maximal oxygen consumption rate (OCR) and extracellular acidification rate (ECAR) were measured using the Seahorse XFe96 analyzer (Seahorse bioscience). For OCR analysis, oligomycin (1.5 µM), FCCP (0.5 µM) and a mixture of antimycin A (0.5 µM) and rotenone (0.5 µM) were injected consecutively with a specific time gap, and OCR values were measured after each injection. For ECAR analysis, similarly, a mixture of antimycin A (0.5 µM) and rotenone (0.5 µM), and 2-deoxy-d-glucose (2-DG) (50 mM) were injected consecutively with a specific time gap and ECAR values were measured after each injection. Data were normalized to the number of cells.

### RNA-seq

Extensively characterized TNBC cell lines, MDA-MB-231, BT-549 and MDA-MB-468 were plated in 6-well plates at 5 × 10^5^ cells/well for 24 h before transfection. Each cell line was transfected with CT at 1.25 µg of mRNA and incubated for 6 h, 12 h and 24 h. RNA was extracted using the RNeasy Plus Mini kit (Qiagen). The RNA integrity was assessed with an Agilent 2100 Bioanalyzer (Agilent Technologies). RNA-seq libraries were generated using Illumina mRNA TruSeq kit with dual index barcoding. Multiplexed libraries were sequenced at the Cold Spring Harbor Laboratory core sequencing facility. Approximately 8,000,000 paired-end 76-bp reads were sequenced per replicate on a HiSeq 2500 instrument in RAPID mode. After removing adaptor sequences with Trimmomatic^21^, RNA-seq reads were aligned to GRCm38-mm10 with STAR^22^. Genome-wide transcript counting was performed by HTSeq or feature counts to generate a fragments per kb of million mapped reads matrix^23^. Differential expression analysis was performed with DESeq2 package in R (ver. 4.1, http://www.bioconductor.org/packages/release/bioc/html/DESeq2.html)^24^. Genes with a real adjusted *p* value were used for downstream analyses. The datasets generated and/or analyzed during the current study are available in the NCBI Gene Expression Omnibus (GEO) Database repository, GSE201421.

### Immunofluorescence microscopy

Cells were plated in a 12-well plate including a cover slip at 1.5 × 10^4^ cells/well and incubated for 24 h. Cells were transfected with 0.625 µg of mRNA and incubated for 6 and 12 h. The transfected cells washed with cold PBS were fixed with 4% paraformaldehyde (HanLab, Seoul, Korea) for 15 min at RT. After additional washing with cold PBS, the fixed cells were permeated with 0.1% Triton X-100 (Sigma) in PBS for 10 min at RT. For cell immuno-staining, cells were incubated in a blocking solution containing 1% BSA (Sigma) and 0.01% Triton X-100 in PBS for 1 h at RT and then incubated with relevant primary antibodies in blocking solution overnight at 4 °C. Primary antibodies were purchased as follows: p-Histon H2A.X(S139) (Cell signaling technology, 2577), GFP (Abcam, ab290). For fluorescence labeling, cells washed with 0.01% Triton X-100 in PBS were stained with Alexa488 or Alexa555-conjugated secondary antibody (Invitrogen) for 1 h at RT in the dark. After additional washes, the nucleus was stained with Hoechst33342 (Sigma) for 1 min at RT in the dark. Images of the mounted cells on microscope slides were generated using a fluorescence microscope (Nikon).

### Statistics and reproducibility

GraphPad PRISM 8 and R software 4.1 were used for statistical analyses. Sample size, error bars and statistical tests are reported in the figure legends. Exact *p* values are shown where possible. *p* value ranges are reported when the number of significant digits exceeded the limits calculated by the statistical package. Statistical tests include unpaired two-tailed Student’s t-test. All sequencing experiments were done once. The number of times experiments were performed with similar results is indicated in each legend.

### Reporting summary

Further information on research design is available in the Nature Research Reporting Summary linked to this paper.

## Results

### Suppression of cellular proliferation by transfection of CT mRNA to MDA-MB-231 cells

The cytosolic thymidylate 5'-phosphohydrolase (CT) derived from PBS2 bacteriophage is known to hydrolyze dTMP into thymidine with higher catalytic efficiency than any other nucleotide monophosphate (Supp. Fig. S2). We hypothesized that CT expression in cancer cells might selectively lower the intracellular dTMP pool and induce nucleotide pool imbalance, inhibiting the growth of cancer cells by disrupting the balanced nucleotide supply required for continuous, unregulated replication.

**Figure 1:**
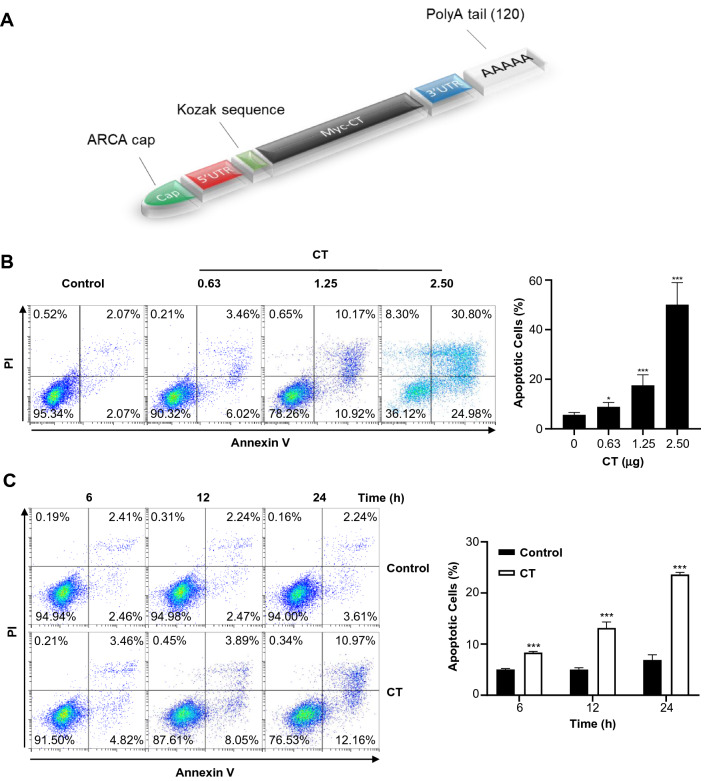

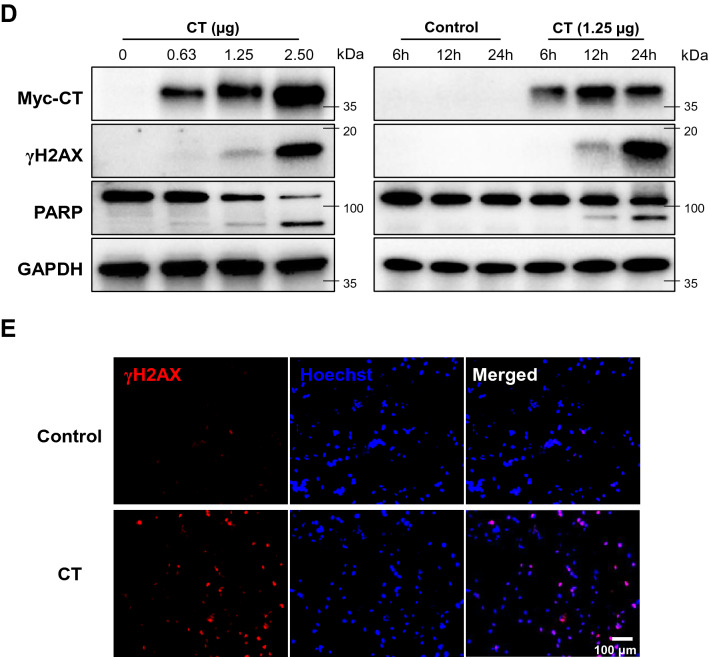
Suppression of MDA-MB-231 cell proliferation by CT mRNA. (**A**) CT-mRNA structure. CT mRNA was transcribed as a construct composed of the CT structural gene flanked with 5′- and 3′-UTRs. For stable translation in cancer cells, ARCA and a poly A tail were added to CT mRNA. (**B**) Flow cytometry analysis of the concentration-dependent cytotoxicity in MDA-MB-231 by CT transfection. Representative results were shown as quadrant analysis of fluorescence intensity of gated MDA-MB231 cells in annexin V-FITC and PI channels. MDA-MB231 cells were incubated without (control) or with CT at concentrations of 0.63, 1.25, 2.5 µg mRNA/5 × 10^5^ cells for 24 h. CT mRNA efficiently induced apoptosis in MDA-MB231 cells. (**C**) Time-course cytotoxicity of MDA-MB-231 cells by CT mRNA transfection (1.25 μg). (**D**) Identification of apoptotic markers by treating with concentration-dependent manner for 12 h (left) and with time-dependent manner with 1.25 µg mRNA (right). Original blots are presented in Supplementary Information (Figure Raw data). (**E**) Visualization of apoptosis in MDA-MD-231 cells at 24 h post-CT transfection. The presence of phosphorylated H2AX (Alexa555-labeld antibody, red) in DNA in nucleus (Hoechst33342, blue) in MDA-MB-231 cells after CT-mRNA transfection were visualized by fluorescence microscopy. Percentages of viable, apoptotic, and necrotic cells without (control) or with CT treatment were represented as the mean of three experiments (n = 3, Student’s t-test *p < 0.05, **p < 0.01, ***p < 0.001, significantly different from the control).

To test this notion and investigate CT-induced cellular inhibitory effects on cancer cells, the codon optimized CT gene was assembled into an optimized mRNA platform for intracellular expression (Fig. 1A). The resulting CT mRNA permits translation of CT appended with a unique N-terminal Myc tag for specific immunodetection. We then examined concentration- and time-dependent apoptotic effects by flow cytometry analysis of gated MDA-MB-231 cells. Figure 1B shows the typical results of quadrant analysis of fluorescence intensity of gated MDA-MB-231 cells in annexin V-FITC and PI-PE channels after treatment with various concentrations of CT mRNA. The lower left quadrant represents viable (normal) cells, the lower right quadrant represents apoptotic (early apoptotic) cells, and the upper right quadrant represents necrotic (late apoptotic) cells. As shown in Fig. 1B, the ratio of the apoptotic cells increased in a concentration-dependent manner. Additionally, the percentage of apoptotic cells increased in a time-dependent manner for MDA-MB-231 cells transfected with CT mRNA (Fig. 1C). In transfecting 1.25 μg of CT to 5 × 10^5^ cells, apoptotic cells were increased up to 23.6 ± 0.35% (n = 3, *p* < 0.001) at 24 h. This suppressive effect of CT mRNA on MDA-MB-231 cell viability was attributed to apoptosis, as evidenced by γH2AX expression and PARP cleavage (89 kDa) (Fig. 1D,E). A previous study reported that cleavage of PARP1 occurs during both necrosis and apoptosis^25^. Shin et al. reported that in the case of necrosis, PARP1 was cleaved by lysosomal protease, resulting in a 55-kDa cleaved-PARP1 (C-PARP1) protein, whereas in the case of apoptosis, caspase 3 cleaves PARP1 to produce an 89-kDa C-PARP1 protein^26^. According to these reports, it was reasonable to regard our case as apoptosis rather than necrosis. Also in cell cycle analysis, dNTP imbalance induced by decrease of dTTP resulted in an increase of sub G1 cell fraction, rather than S-phase arrest of cell cycle which is mainly observed in increased dNTP imbalance (Supp. Fig. S3). Based on these findings we next investigated if the apoptotic induction in MDA-MB-231 cells may be related to the change in their intracellular nucleotide levels, caused by reprogramming metabolic pathways to compensate for dTTP deficiency.

### Changes in cellular metabolism following transfection of CT mRNA in MDA-MB-231 cells

**Figure 2:**
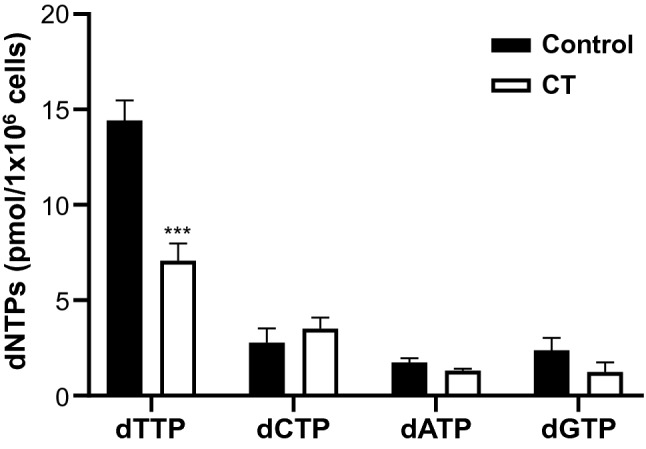
Changes in intracellular dNTP pools in MDA-MB-231 following CT transfection. EvaGreen-based assay for dNTPs using 197-nt templates was carried out for quantification of dNTP pools in MDA-MD-231 cells after CT transfection (1.25 μg/5 × 10^5^ cells).

**Figure 3:**
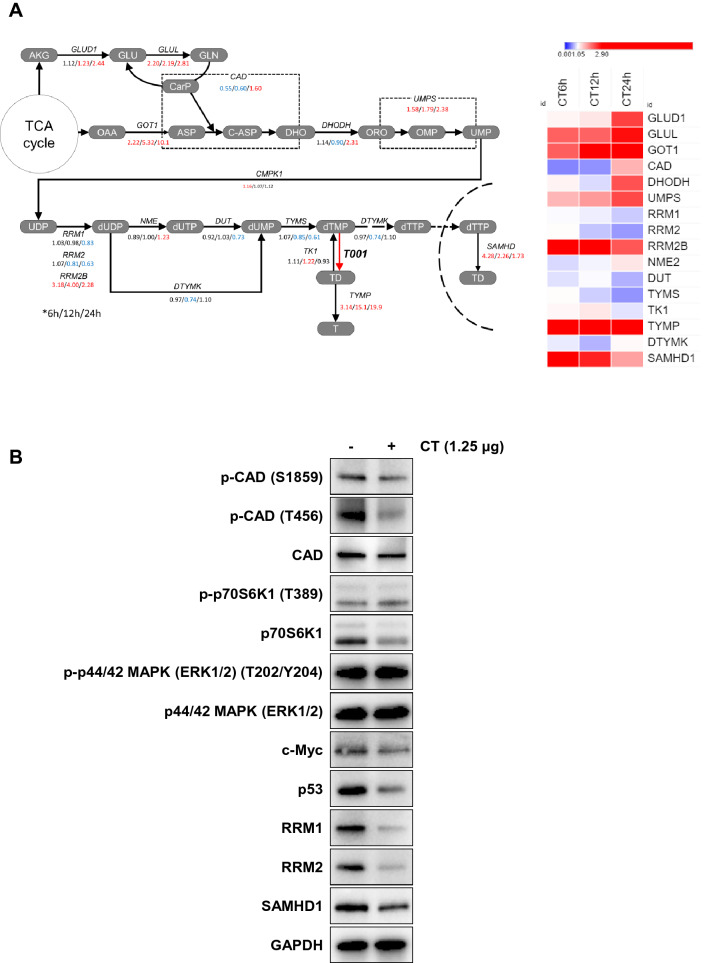
Expressional changes of essential pyrimidine biosynthetic enzymes regulated by oncogene driven signaling. (**A**) RNAseq analysis of pyrimidine metabolic pathway (6 h/12 h/24 h). AKG: α-ketoglutarate, GLU: glutamate, GLN: glutamine, CarP: carbamoyl phosphate, OAA: oxaloacetate, ASP: aspartate, C-ASP: carbamoyl-aspartate, DHO: dihydroorotate, ORO: orotate, TD: thymidine, T: thymine, GLUD: glutamate dehydrogenase, GLUL: glutamate-ammonia ligase, GOT: glutamic-oxaloacetic transaminase, CAD: carbamoyl-phosphate synthetase, aspartate transcarbamylase, and dihydroorotase complex, DHODH: dihydroorotate dehydrogenase, UMPS: UMP synthetase, RRM1/2: ribonucleotide reductase catalytic subunit M1/2, RRM2B: ribonucleotide reductase regulatory TP53, NME: nucleoside diphosphate kinase, DUT: dUTPase, TYMS: thymidylate synthase, DTYMK: deoxythymidylate kinase, TK1: cytosolic thymidine kinase, TYMP: thymidine phosphorylase, SAMHD: SAM and HD domain containing dNTP triphosphohydrolase, T001: enzyme translated from CT. (**B**) Western blot analysis of MDA-MB-231 cells was carried out to investigate the expression changes of essential pyrimidine biosynthetic enzymes and the oncogene-driven signaling proteins. Original blots are presented in Supplementary Information (Figure Raw data).

Indeed, intracellular dTTP level was largely decreased compared with the other nucleotide levels in CT-transfected MDA-MB-231 cells as shown in Fig. 2. This observation indicated that CT can induce dNTP imbalance by selective dTMP degradation. The coordinated synthesis and degradation of dNTPs to maintain a balanced intracellular dNTP pool are critical to ensure the fidelity of DNA synthesis and DNA damage repair^27^. The observed decreased intracellular dTTP level may affect two distinct pathways of dNTP synthesis in order to recover from the intracellular dNTP pool imbalance – de novo synthesis in the cytoplasm or the salvage pathways in the cytoplasm and mitochondria. Building on the connection between pyrimidine metabolism and CT expression, we sought to identify the metabolic pathways specifically changed in CT-treated cells. As shown in the schematic pathway obtained from RNAseq analysis, a few rate-limiting pyrimidine biosynthetic enzymes (CAD and TYMS) were decreased immediately after CT treatment and then recovered, whereas a thymidine-degradation enzyme (TYMP) and feedback regulated enzyme (UMPS) were upregulated (Fig. 3A). Additionally, the influx from TCA cycle intermediates was increased for facilitating to supply the precursors required in pyrimidine synthesis (GLUD1, GLUL and GOT).

On the other hand, overall changes in the dNTP pool can generally perturb cell cycle-dependent activity and allosteric regulation of ribonucleotide reductase (RNR) and mammalian triphosphohydrolase (SAMHD1)^26^. Indeed, SAMHD1 mRNA expression was increased by 4.3-fold at 6 h, and then slowly decreased (Fig. 3A). Even though this decreasing trend in the amplified transcription of SAMHD1 at 24 h might result in loosening the regulation of CAD, DHODH and UMPS, the critical enzymes involved in direct influx of dTMP (TYMS and TYMP) did not appear to be recovered to normal levels in cancer cells. This means that nucleotide imbalance driven from dTMP degradation can put upward pressure on the feedback regulated enzymes, but rather put pressure on stopping the pyrimidine synthesis in terms of signal transduction.

Considering the significant increase in SAMHD1 mRNA observed in MDA-MB-231 cells during CT treatment, immunoblotting was performed to determine if the increase in SAMHD1 mRNA directly reflected the expression of SAMHD1 protein. Interestingly, even though SAMHD1 mRNA was largely up-regulated in MDA-MB-231 cells (Fig. 3A), SAMHD1 protein level was not detectably increased (Fig. 3B). This discrepancy suggests that post-transcriptional and/or post-translational regulations might also contribute to SAMHD1 protein expression in different cell types.

Apart from CAD transcript level, CAD function is regulated in multiple ways, including allosteric regulation, covalent regulation by phosphorylation, and metabolite channeling^28^. Phosphorylation of CAD on Ser1859 (S1859) is regulated by (mTOR)-S6K^29,30^, the mammalian target of rapamycin, whereas phosphorylation on Thr456 (T456) is regulated by EGFR^31^. In our case, phosphorylation of CAD on T456 was significantly decreased, but levels of unmodified and S1859 phosphorylated CAD proteins were slightly decreased or not changed, compared with control (untreated) at 24 h (Fig. 3B). Accompanying this metabolic change in the presence of CT, mutated oncogenes like c-Myc and p53 were also found to be decreased (Fig. 3B). Generally, the energy metabolism of MDA-MB-231 is rewired by mutated oncogenic malfunction (gain-of-function or loss-of-function), which can help cancer cells to survive and adapt in the tumor microenvironment (TME)^32,33^. Since redox homeostasis can affect various target genes and proteins, the decrease of c-Myc and mutated p53 in MDA-MB-231 was expected to be somewhat related to the slow-down in energy metabolism and disturbance of TME adaptation of cancer by impaired redox homeostasis. Nevertheless, increased metabolic vulnerability may be a more significant cause of cell death compared to direct signal transduction^6^.

**Figure 4:**
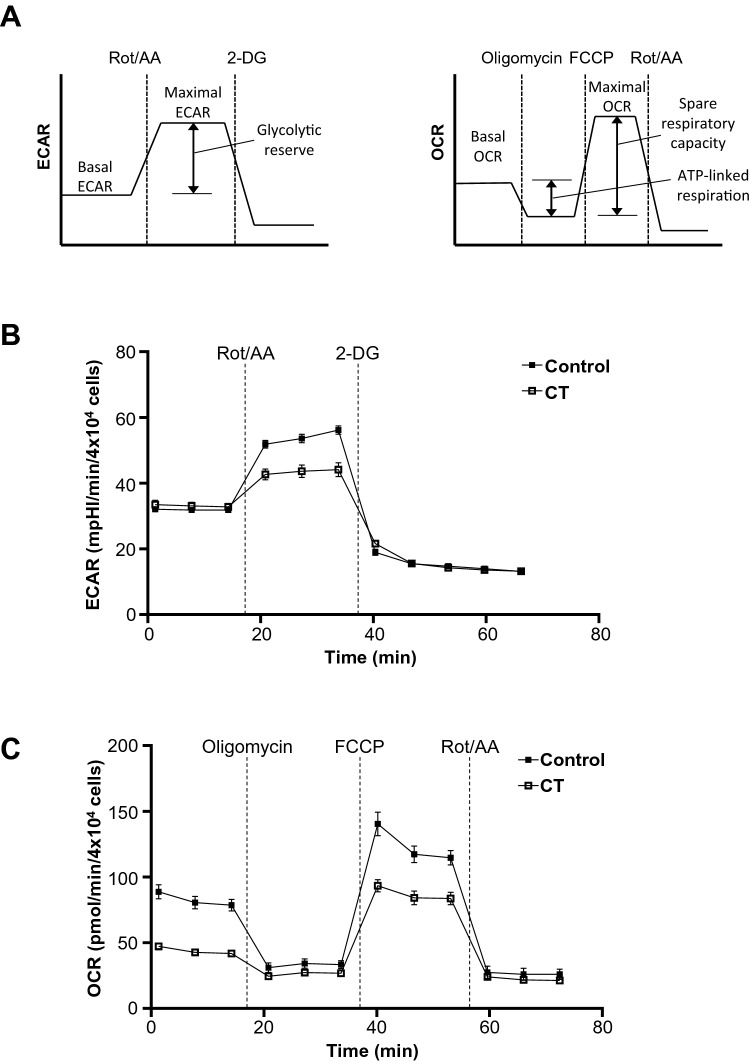
MDA-MB-231 maximal metabolic profiles with CT transfection. (**A**) Schematic indicating derivation of maximal metabolic rates, glycolytic reserve, and spare respiratory capacity from the experiment in (**B**,**C**). (**B**) ECAR assay for MDA-MB-231 transfected with CT. Cells were maintained in uniform media prior to measuring ECAR. ECAR was measured twice in the basal state, and then twice again following each metabolic inhibitor treatment (0.5 μM rotenone/0.5 μM antimycin A, 50 mM 2-DG) at 7 min intervals. (**C**) OCR assay for MDA-MB-231 transfected with CT. OCR was measured twice in the basal state, and then twice again following each metabolic inhibitor treatment (1.5 µM oligomycin, 0.5 μM FCCP, 0.5 μM rotenone/0.5 μM antimycin A) at 7 min intervals. ECAR and OCR values were normalized to a measurement of total cell numbers for each cell line in each assay. Data are their averages from one representative experiment, with error bars representing SD.

Indeed, nucleotide metabolism is closely linked to energy metabolism, and biosynthesis of nucleotides in cancer cells is essential for cell proliferation. Since cancer cells continuously proliferate and divide, various metabolites and sufficient energy for survival are normally supplied from glycolysis, the TCA cycle, and oxidative phosphorylation. As shown in the glycolytic rate (extracellular acidification rate, ECAR) and oxygen consumption rate (OCR) analysis, both basal and maximal OCR were largely decreased in CT-treated MDA-MB-231 cells, whereas maximal ECAR was decreased but basal ECAR was not (Fig. 4). The observation that basal ECAR was largely unchanged by CT treatment, indicated that the glycolytic intermediates were not shunted to other pathways as much as other TCA intermediates in the adapted state of cancer. In other words, these results indicated that TCA cycle intermediates were more directly used in nucleotide biosynthesis, but glycolytic intermediates could be indirectly required via anaplerotic reaction for the TCA cycle. If prolonged in this metabolic state, the TCA cycle impaired by the overconsumption of pyrimidine deoxynucleotides could cause severe burden to redox homeostasis of MDA-MB-231 cells. Indeed, the NADP/NADPH ratio of CT-transfected cells was significantly increased, indicating that these cells could have a serious trouble in controlling the intracellular redox balance (Supp. Fig. S4). Taken together, regardless of whether energy metabolism is reprogrammed and adapted by oncogenic malfunction in MDA-MB-231 cells or not, the local overconsumption of pyrimidine deoxynucleotides could severely burden the redox homeostasis of metabolically vulnerable cancer cells, like the so-called butterfly effect. Additionally, these severe burdens could induce apoptosis accompanied with mitigating dysfunction by oncogenic proteins as shown in Fig. 3.

### Differences in substrate preference for dTMP among 5′-nucleotidases

Given the necessity of cancer cells to generate sufficient nucleotides to meet the demand for increased DNA replication and RNA synthesis and observations that the pyrimidine pathway is inappropriately activated in most cancers, various cellular functions including nucleotide synthesis may be seriously affected by a dNTP pool imbalance. As shown previously, the dNTP pool imbalance caused by CT severely burdened breast cancer cells. A recent study on the physiology of cancer cells showed an increase of dNTP pools was accompanied by higher mutation rates due to reduced fidelity of DNA replication^34^. In contrast, early studies showed that a decrease in dNTP pools by hydroxyurea (HU) lead to an S-phase arrest by replication stress^35,36^. It seems that not only are the overall levels of dNTPs important for genome stability but also the balance between individual dNTPs, which can lead to positive or negative effects on cancer cell therapy. Based on this as a cancer treatment approach, it is necessary to induce dNTP perturbation towards inhibiting cell growth and not triggering rescue mutations. This dNTP perturbation is closely related to dNTP availability, which depends on the specificity of the 5'-nucleotidase used to induce dNTP imbalance. Thus, the specific decrease of dTTP levels rather than the decrease in the overall dNTP pool possibly induced dNTP imbalance more effectively, which was expected to lead to cell death distinguishable from use of a conventional inhibitor for nucleotide synthesis.

**Figure 5:**
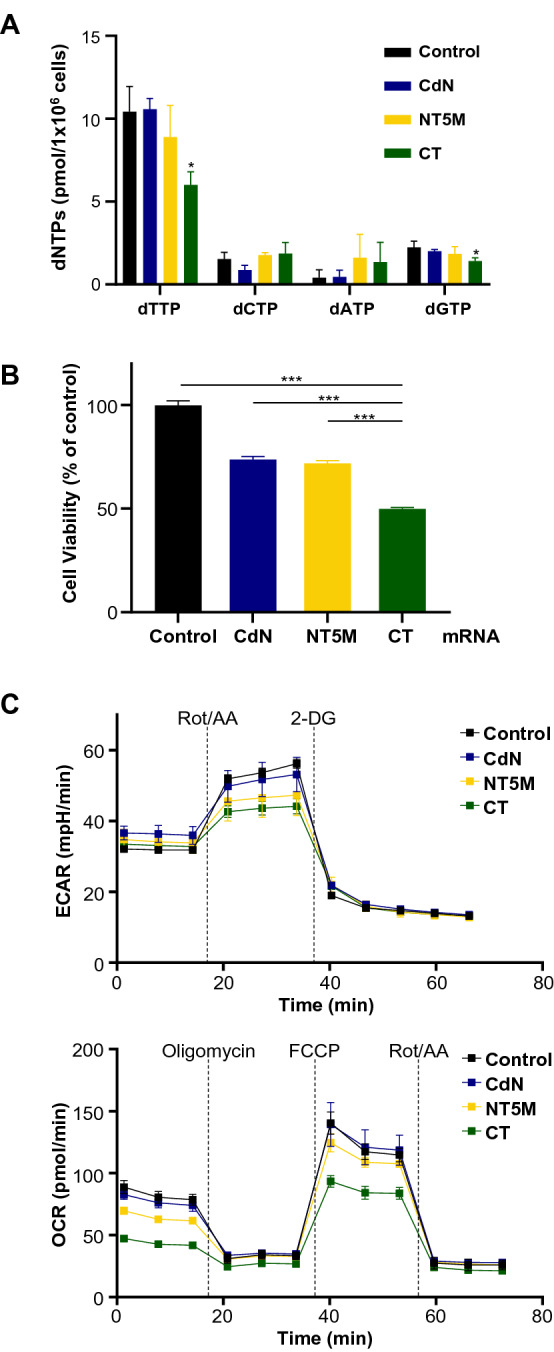

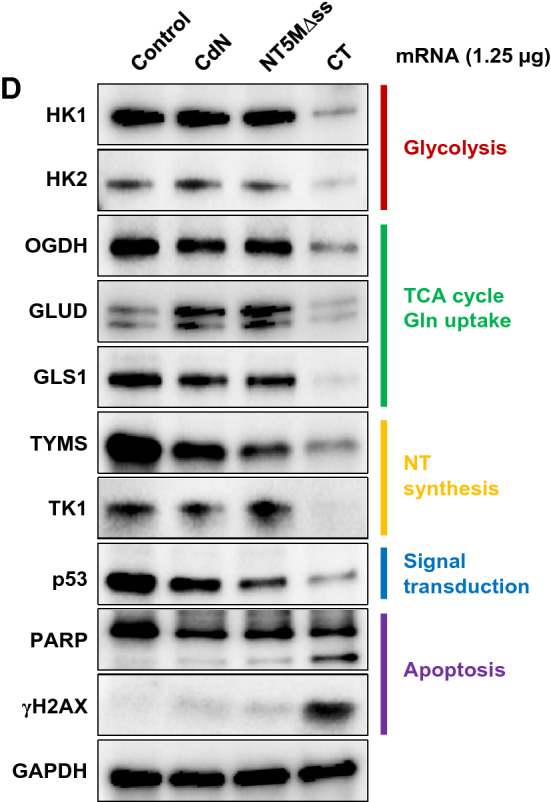
Cellular effects on differences of substrate preference on dTMP. (**A**) Intracellular dNTP pools with 5′ nucleotidases with different substrate preferences. CdN: cytosolic 5′-nucleotidase; NT5M: human mitochondrial 5′-nucleotidase (signal-sequence deleted). (**B**) Cell viability assay. Cells were plated 24 h in a 6-well plate at 5 × 10^5^ cells/well, then transfected with 1.25 µg of mRNA for 24 h. For quantification, the amount of formazan dye formed was measured at 450 nm. (**C**) ECAR and OCR assay for comparing 5′-nucleotidase substrate preferences in MDA-MB-231. (**D**) Western blot analysis for representative proteins related to cellular function. Hexokinase (HK1/2) in glycolysis; α-ketoglutarate dehydrogenase (OGDH) in important branching position of TCA cycle; glutamate dehydrogenase (GLUD) and glutaminase (GLS1) in glutaminolysis; thymidylate synthase (TYMS) and thymidine kinase (TK1) in essential entry position of dTTP synthesis; cleaved poly ADP-ribose polymerase (PARP) and phosphorylated H2AX (γH2AX) as apoptosis marker. Original blots are presented in Supplementary Information (Figure Raw data).

Herein, we observed that the specific decrease of dTTP by CT could directly destabilize the dNTP pool and investigated whether the degree of dNTP pool imbalance was directly related to cell viability and the frequency of mutations during replication. The effects on MDA-MB-231 cells based on the specificity of the 5'-nucleotidase were analyzed by the differences in the dNTP pools, cell viability, and ECAR/OCR (Fig. 5). As proof-of-concept, mRNA constructs of human cytosolic 5′-nucleotidase (CdN) and human mitochondrial 5′-nucleotidase (signal-sequence deleted) (NT5M) which are known to have different substrate preferences, were created by replacing the CT gene in the existing construct (Supp. Fig. S2). As shown in Fig. 5A, dTTP levels in the presence of NT5M and CT were further decreased, compared to CdN which has broad dNTP specificity. In contrast, cell viability trends were similar overall to their dTTP levels. Notably, the lowest dTTP level among the tested 5′ nucleotidases was observed with CT (Fig. 5B).

Even though the difference between NT5M and CT was not clear solely based on dNTP level analysis, CT appeared to have much more burden to OXPHOS than glycolysis in OCR/ECAR analysis, compared to NT5M. As shown in OCR analysis (Fig. 5C), CT showed both lower basal respiration and glycolytic reserve than NT5M. Also in ECAR analysis, the ECAR profile of CT was similar to that of NT5M, but the lowest glycolytic reserve in the tested group was shown with CT. These results indicated that cell viability could be affected by catalytic activity as well as substrate preference of the enzymes, and the CT transfected cells were forced to exploit TCA cycle to a greater extent to supply pyrimidine than with either CdN or NT5M. Many researchers have reported that the excess pyrimidine biosynthesis in normal cells could be induced by upregulating TCA cycle enzymes in dNTP deficient condition^37^. However, unlike normal cells, metabolically vulnerable TNBC cells (MDA-MB-231) experienced severe metabolic stress due to the overconsumption of glucose and glutamine caused by upregulation of TCA cycle enzymes. This accumulated metabolic fatigue resulted in deactivating glycolysis and the TCA cycle by resource-burnout, ultimately leading to apoptosis. Induction of apoptosis was assessed by examining phosphorylation of H2AX at Ser139 (γH2AX) which marks DNA double-strand breaks and the levels of cleaved PARP (Fig. 5D). In CT mRNA-transfected MDA-MB-231 cells, bands of γH2AX and cleaved PARP were significantly increased, compared with CdN and NT5M. Since the degree of apoptosis appeared to differ with the differing substrate specificity of the 5'-nucleotidase used, these results supported the theory that substrate specificity was closely related to apoptotic effects in TNBC cells.

**Figure 6:**
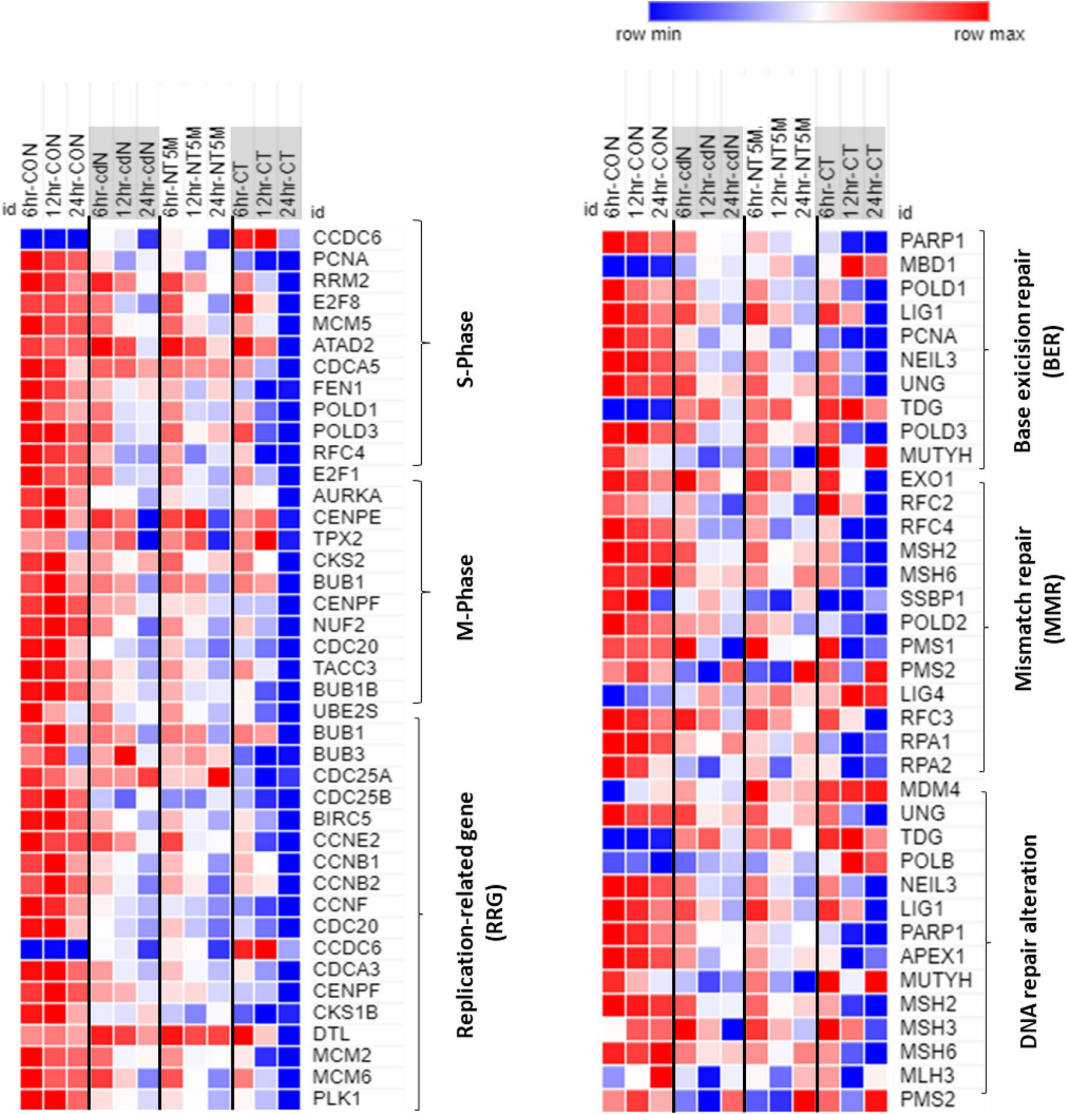
RNAseq analysis of cancer-helping dysregulated gene transcriptome in MDA-MB-231 cells transfected with 5′-nucleotidases with different specificities on dNMP. From whole RNAseq data, gene sets related to S or M-phase checkpoint and replication/repair were extracted and sorted. The arranged RNAseq data showed that the expression pattern was gradually reversed depending on the specificity of 5′nucleotidases for dTMP. CCDC6, coiled-coil domain containing 6; PCNA, proliferating cell nuclear antigen; RRM2, ribonucleotide reductase regulatory subunit M2; E2F8, E2F transcription factor 8; MCM5, minichromosome maintenance complex component 5; ATAD2, ATPase family AAA domain containing 2; CDCA5, cell division cycle associated 5; FEN1, flap structure-specific endonuclease 1; POLD1, DNA polymerase delta 1; POLD3, DNA polymerase delta 3; RFC4, replication factor C subunit 4; AURKA, aurora kinase A; CENPE, centromere protein E; TPX2, microtubule nucleation factor; CKS2, CDC28 protein kinase regulatory subunit 2; BUB, mitotic checkpoint serine/threonine kinase; CENPF, centromere protein F; NUF2, NUF2 component of NDC80 kinetochore complex; CDC20, cell division cycle 20; TACC3: transforming acidic coiled-coil containing protein 3; UBE2S, ubiquitin conjugating enzyme E2 S; CDC25A/B, cell division cycle 25A/B; BIRC5, baculoviral IAP repeat containing 5; CCNE2, cyclin E2; CCNB1/2, cyclin B1/2; CCNF, cyclin F; CDCA3, cell division cycle associated 3; CKS1B, CDC28 protein kinase regulatory subunit 1B; DTL, denticleless E3 ubiquitin protein ligase homolog; MCM, minichromosome maintenance complex component; PLK1, polo like kinase 1.

Clinical behavior of breast cancers is linked to high proliferative activity and mutational burden^38–40^. Recently, a notable study reported the expression of replication-related genes (RRGs) in TNBC cells^41^. The expression of RRGs was explored in various TNBC subtypes and showed that TNBC cells exhibited high expression of RRGs, indicating elevated replication activity. DNA replication consumes substantial energy and nucleotide resources, and may imbalance the nucleotide pool, causing replication stress and increased mutational burden^42,43^. Consistent with those studies, our RNAseq data similarly showed that control MDA-MB-231 cells exhibited high expression of replication-related genes (RRG, t-test *p* < 0.001) along with increased expression of BER and MMR gene sets (Fig. 6 and supp. Fig. S5A). Surprisingly, we found differences among CdN, NT5M and CT-treated cells in the expression of all clustered categories including the RRG set. For CdN and NT5M, general stress responses induced by nucleotide-deficiency during DNA replication were observed, and the transcriptional profiles appeared to be intermediate between those of control and CT (Fig. 6 and Supp. Fig. S5A). In contrast, most of the expression in both the RRGs set and the repair system set of CT mRNA-transfected cells were reduced or even shut down. TNBCs with compromised p53 function exhibit a prevalence of C-to-T transition, typically associated with misincorporation of nucleotides^44,45^. Misincorporated nucleotides are normally removed by DNA repair mechanisms such as MMR and BER, but TNBC cells with inadequate activity or genetic alterations in these mechanisms may exhibit an increase in mutational frequency^44,46,47^. Expression of the 5'-nucleotidases in these genetically altered TNBCs could make the metabolism of TNBC more vulnerable, further exacerbating or alleviating the mutational burden depending on the substrate specificity of the 5' nucleotidase (Supp. Fig. S5A).

Notably, the expression of CCDC6, MBD1, TDG, LIG4, MDM4, and POLB, which are hardly expressed in MDA-MB-231, were significantly increased as the substrate specificity of the 5'-nucleotidases became narrower, whereas PCNA, FEN1, CDC20, CDC25AB, PLK1, and PARP1, which are upregulated in human cancer, were markedly decreased (Fig. 6 and Supp. Table S1). Replication-related genes and check point genes, such as those listed above, are known as cancer-helping genes, driving various uncontrolled cellular responses in cancer cells. Thus, these findings were encouraging, showing that the tumor-adaptive functions of MDA-MB-231 cells were completely inverted by CT expression. Expanding on these results, we could infer that, regardless of oncogenic mutations in TNBC cells, such as MDA-MB-231, the progression of the cell cycle and DNA replication could be suppressed even by the induced metabolic burden caused by CT (Fig. 6 and Supp. Fig. S5B).

### Metabolic effects of CT according to TNBC cell subtypes

Overall TNBC cell models and patient samples are characterized by elevated glycolysis, compared to ER+ breast cancer cell lines^48^. In particular, MDA-MB-231 and MDA-MB-468 TNBC cell models were reported to harbor high glycolytic flux and low OXPHOS activity, which showed that TNBC cells could switch to a glycolytic program under conditions of limited oxygen more easily than non-TNBC cells^48,49^. These metabolic characteristics of TNBC may be due to either impaired mitochondrial activity or metabolic needs for tumorigenesis of TNBC cells. Above, we revealed that cells with CT expression were increasingly forced to exploit the TCA cycle to supply pyrimidine. Since glycolysis was not equally exploited, we hypothesized that the more a certain cell line was dependent on mitochondrial energy metabolism, the more cell viability was damaged by CT expression. To elucidate the effects of CT according to diverse metabolic activity in TNBC cell lines, we examined three TNBC cell lines which are representative of several TNBC subtypes identified by Lehmann et al^50^. These cell lines were BT-549 (mesenchymal stem-like (M); basal B), MDA-MB-231 (mesenchymal stem-like (MSL); basal B) and MDA-MB-468 (basal-like1 (BL1); basal A) cells.

**Figure 7:**
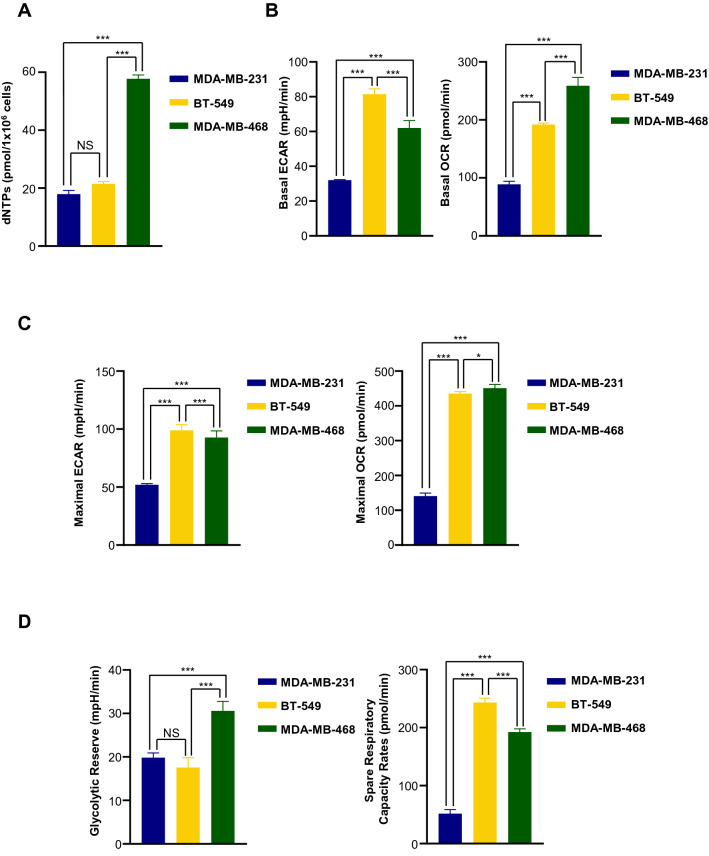

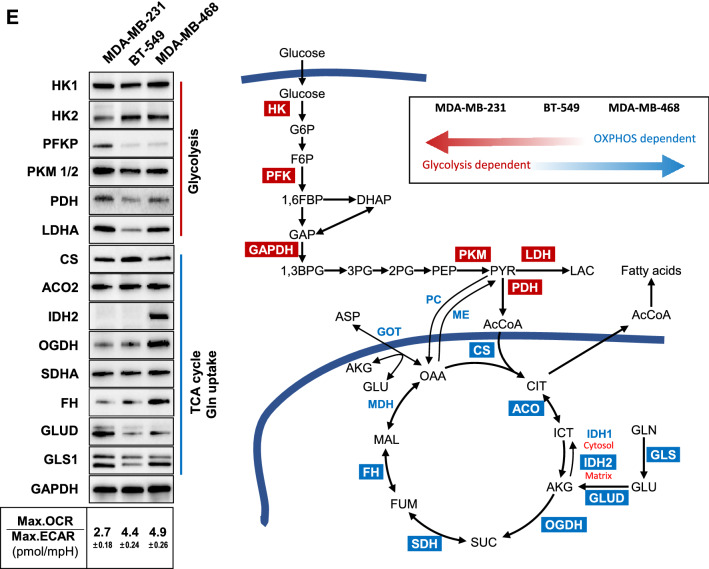
Effects of CT on energy metabolism according to TNBC cell subtypes. (**A**) Quantification of total dNTP levels of three TNBC cell lines with different genetic backgrounds. Considering Lehmann classification, three representative cell lines of TNBC subtypes were determined as BT-549 (mesenchymal stem-like (M); basal B), MDA-MB-231 (mesenchymal stem-like (MSL); basal B) and MDA-MB-468 (basal-like1 (BL1); basal A) cells. (**B**) Basal ECAR and basal OCR analysis of three TNBC cell lines. (**C**) Maximal ECAR and basal OCR analysis of three TNBC cell lines. (**D**) Glycolytic reserve and spare respiratory capacity rate of three TNBC cell lines. Metabolic profiles were measured using an extracellular flux analyzer (Seahorse Bioscience). ECAR and OCR values were normalized to a measurement of total cell numbers for each cell line in each assay. Data are averages from one representative experiment, with error bars representing SD. (**E**) Metabolic dependency of three TNBC cells tested. Original blots are presented in Supplementary Information (Figure Raw data).

In Fig. 7A, the MDA-MB-468 cell line showed much higher dNTP levels compared with 2 mesenchymal stem-like cell lines (BT-549 and MDA-MB-231). Excess nucleotide biosynthesis mainly occurred in cancer cells where the glutamine consumption rate is increased^51,52^. In order to resolve the redox stress (NADH overproduction) caused by rapid glucose consumption via only glycolysis under limited oxygen conditions, cancer cells may switch from glucose supply usage to glutamine to lower redox stress. In addition to serving as a carbon source, glutamine is important as a nitrogen donor for cancer cell growth, supporting the increased demand for nucleotides^51^. MDA-MB-231 cells exhibited the lowest glycolytic rates (ECAR) among the tested TNBC cells, while BT-549 and MDA-MB-468 cells displayed approximately 2.5 and 2 times the glycolytic rate of MDA-MB-231 cells, respectively (Fig. 7B). Meanwhile, BT-549 and MDA-MB-468 displayed approximately 2.2 and 3 times the OCR of MDA-MB-231 cells. It appeared that the difference in dNTP levels among cancer cell lines was more strongly dependent on OCR than on glycolysis rate. For example, although the glycolysis rate of BT-549 cells was higher than that of MDA-MB-468, dNTP levels of BT-549 did not increase significantly. Accordingly, MDA-MB-468 cells showing the highest OCR also showed the highest dNTP levels.

Next, we tested whether cells are able to alter metabolic pathways to compensate for lacking metabolites when another pathway is perturbed. As with previous reports, inhibition of one metabolic pathway (e.g., glycolysis) allows measurement of the maximal capability of the other pathway (e.g., oxidative metabolism)^48^. To determine the maximal glycolytic and oxygen consumption capabilities in these model TNBC cells, we measured respiration arrest-induced maximal glycolytic rates and depolarization-induced maximal OXPHOS rates (Fig. 7C). The MDA-MB-231 cells exhibited the least metabolic flexibility, as demonstrated by calculated glycolytic reserve and spare respiratory capacity rates, whereas the MDA-MB-468 cells exhibited the greatest metabolic flexibility (Fig. 7D). When western blot analysis is taken into consideration with maximal ECAR/maximal OCR ratios, MDA-MB-231 cells appeared to be more glycolysis-dependent, while MDA-MB-468 was more OXPHOS-dependent (Fig. 7C,E). This observation indicated that MDA-MB-468, unlike the other 2 cell lines, could convert more glutamine to aspartate, enabling a high level of pyrimidine synthesis. Indeed, glutamine influx from α-KG to OAA in the TCA cycle was upregulated to a greater extent in MDA-MB-468 compared with the other 2 cell lines. This result showed that the level of dNTPs, especially pyrimidines, required in the cell were closely related on the dependence on mitochondrial metabolism rather than on glycolysis (Fig. 7E). Metabolic capacity and adaptability could be a major difference of mesenchymal-like versus basal-like subtypes according to studies on drug responses of metabolic rates and principle metabolic components^9,48^. Similarly, in our study, basal-like cell line, MDA-MB-468, showed both higher OCR and ECAR, resulting in an increased dNTP pool. On the other hand, mesenchymal-like cell lines, MDA-MB-231 cells, had both lower OCR and ECAR, corresponding to a decreased dNTP pool. The other mesenchymal-like cell line, BT-549 cells, showed intermediate characteristics of these different cell types, with low dNTP pools, but relatively higher OCR and ECAR than MDA-MB-231 cells. Taken together, the expression profiles of essential rate-limiting enzymes agreed with the tendency observed in maximal OCR/ECAR ratio and these findings indicated that metabolic dependency could be classified according to TNBC subtypes. Interestingly, even though the measured values using cultured cells in different media seemed to be quite different to each other, our observation using Plasmax media (FBS 2.5%) was shown to have similar tendency to the result (DMEM, FBS 10%) obtained by Lanning et al^48^.

**Figure 8:**
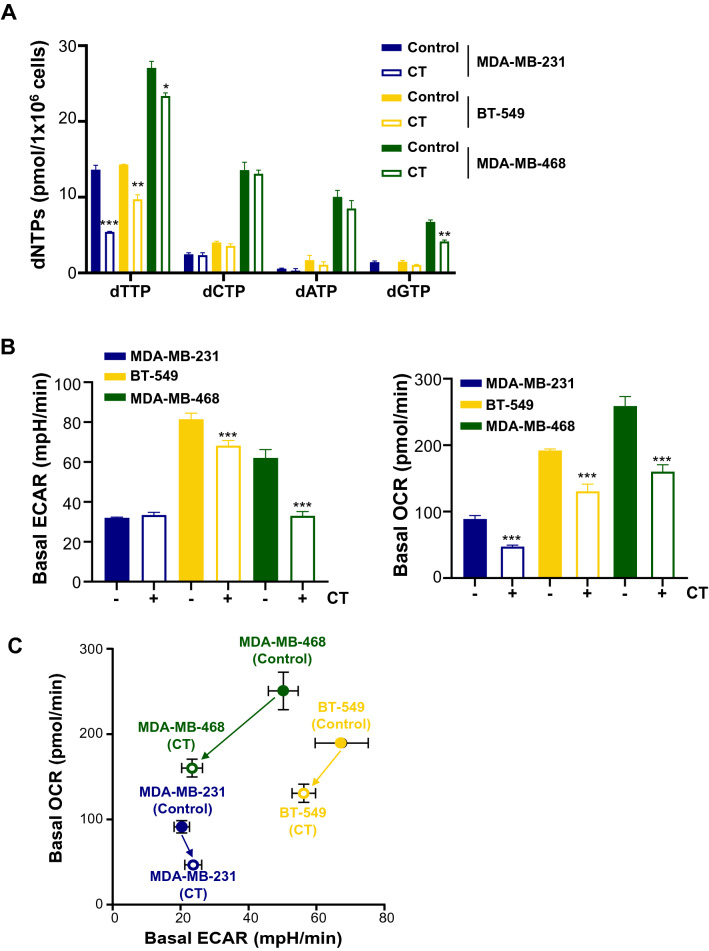

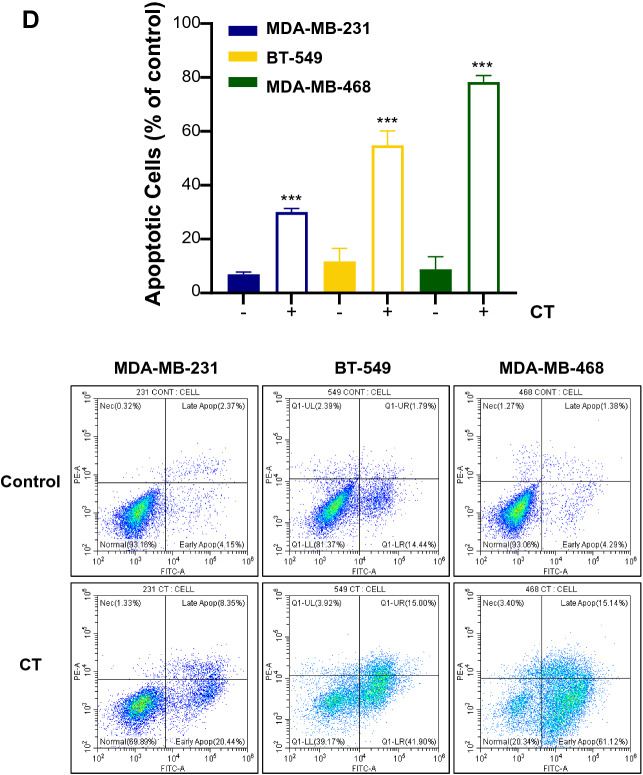
Metabolic effects of CT mRNA transfection in TNBC cells. (**A**) Change of intracellular dNTP pools in TNBC cells by CT transfection. EvaGreen-based assay for dNTPs using 197-nt templates was carried out for quantification of dNTP pools in TNBC cells after CT transfection (1.25 μg/5 × 10^5^ cells). (**B**) Basal ECAR and OCR of TNBC cells transfected by CT. (**C**) Metabolic shift by CT transfection plotted from relative OCR and ECAR data from (**B**). Relative OCR and ECAR data were plotted simultaneously to reveal changes of overall relative basal metabolic profiles for each TNBC cell. The length between control and CT indicated the degree of metabolic shift. (**D**) The ratios of apoptotic cells induced by transfecting CT to each TNBC cell line. In flow cytometry analysis, the ratio of apoptotic cells was proportional to the degree of metabolic shift. Annexin V-FITC positive/PI-PE negative cells and annexin V-FITC/PI-PE double-positive cells were judged to be in the early and late stages of apoptosis, respectively.

**Figure 9:**
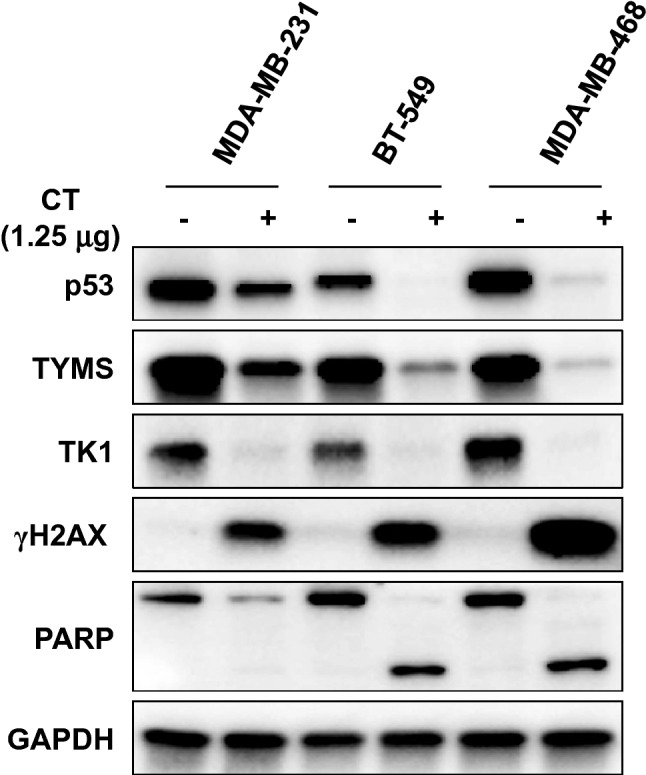
Western blot analysis of CT transfected TNBC at 12 h. All TNBC cells used in this study have mutant p53 as follows; p53(R280K) in MDA-MB-231, p53(R249S) in BT549, and p53(R273H) in MDA-MB-468^76^. The band for p53 in MDA-MB-231 represents the mutant p53 as the smaller protein and the other bands for BT549 and MDA-MB-468 as the larger protein. Original blots are presented in Supplementary Information (Figure Raw data).

To verify the metabolic effects of CT mRNA transfection, we also assessed dNTP pool size and metabolic rates of TNBC cells exhibiting various metabolic flexibility. As shown in Fig. 8A, the dTTP pool appeared to be decreased to a lesser extent in MDA-MB-468 cells with OXPHOS-dependency rather than in MDA-MB-231 cells with glycolysis-dependency, suggesting a strong tendency to replenish dTTP via a high metabolic rate. Consistent with these results, both basal OCR and ECAR showed the greatest decrease in CT-transfected MDA-MB-468 cells, followed by BT-549 cells and MDA-MB-231 cells (Fig. 8B). Looking at the trends in relative basal metabolic rates after CT mRNA transfection, MDA-MB-468 cells appeared to show greater metabolic shift compared to other cells (Fig. 8C). Overall metabolic rate profiles indicated a critical focus on metabolic flexibility. Indeed, large metabolic shifts in cancer cells can mean narrow metabolite availability, which can impose a severe burden on metabolically vulnerable cells. Therefore, cancer cells with higher metabolic flexibility and nucleotide levels could rapidly undergo apoptosis by irreversible disruption of reprogrammed metabolic flux, driven by the specific depletion of dTTP due to the narrow substrate specificity of transfected CT. In alignment with the metabolic rate data above, the CT mRNA-transfected TNBC cells consistently exhibited the trends of apoptotic death as observed by flow cytometry (Fig. 8D). This was confirmed by increased γH2AX and cleaved PARP in western blot analysis (Fig. 9). Also, TYMS and TK1, which are involved in the de novo and salvage pathways, were decreased corresponding to the degree of metabolic shift of TNBC cells in MDA-MB-231, and a decrease in mutated p53 oncogene accompanied the metabolic changes induced by CT (Fig. 9). Although wild type p53 is known to play an important role in tumor suppression, it is likely that the CT-induced reduction of mutated p53 in TNBC adversely affected cancer survival and TME adaptation.

Taken together, dTTP depletion caused by selective dTMP degradation may require excessive aspartate for pyrimidine biosynthesis and reprogramming to incorporate glutamine metabolism into the TCA cycle. The degree of metabolic shift in cancer cells could be determined by their metabolic flexibility between glycolysis and OXPHOS, which could increase metabolic vulnerability in p53 mutated TNBC cells. Especially since aspartate and glutamate are required for de novo pyrimidine synthesis, NADPH generation by a malic enzyme, and glutathione synthesis, dTTP depletion caused by selective dTMP degradation is presumed to induce apoptosis derived from excessive OAA consumption and disruption of redox homeostasis.

## Discussion

In our study, we uncovered a possible therapeutic option in managing advanced breast cancers involving TNBC, which is known as a major clinical problem with limited therapeutic options. The current concept in TNBC therapy is that the metabolic change for maintaining a balanced nucleotide pool in proliferating cells can be exploited to over-biosynthesize nucleotides regardless of metabolic availability, inducing cell death by activating opposing signaling pathways. We showed cells with greater metabolic flexibility, such as MDA-MB-468, are able to better maintain the dTTP pool compared to cells with lower metabolic flexibility, such as MDA-MB-231. We also demonstrated that a strong push to replenish dTTP driven by the high metabolic rate in MDA-MB-468, led to apoptotic death by largely burdened metabolic shifts in glycolysis and OXPHOS (Fig. 9). We found dTTP depletion caused by the selective dTMP degradation, could induce apoptosis differently according to substrate specificity of the 5′ nucleotidase and cellular metabolic vulnerability. It also appears that the greater the metabolic burden on already vulnerable cells, the stronger the cancer cells' tendency to attempt to recover by overusing the vulnerable metabolic pathways to survive. The bias towards use of these vulnerable metabolites could cause ROS accumulation and disruption of redox homeostasis in cancer cells, and the accumulation of this intracellular damage can trigger apoptosis.

According to a previous report, the genotoxicity and increased mutagenesis in cancer cells were known to frequently occur by overall changes in the dNTP pool^34,53^. However, in normal cells, to protect DNA damage by overall increase of the dNTP pool, cell cycle arrest can be occurred by S-phase entry^54,55^. In mammalian cells, the cell cycle regulation of the two main enzymes controlling dNTP pool sizes is adjusted to the requirements of DNA replication. Synthesis by the ribonucleotide reductase peaks during S-phase, and catabolism by SAMHD1 is maximal during G1 phase when large dNTP pools would prevent cells from preparing for a new round of DNA replication^55^. Franzolin et al. attempted to increase dNTP concentrations by silencing SAMHD1, a key dNTP catabolic enzyme that coordinates the dNTP pool with the cell cycle^55^. They demonstrated that SAMHD1 is accompanied by the cell-cycle regulation of dNTP pool sizes and found that dGTP is the preferred substrate of SAMHD1 showing the largest effect in all cases. We observed that CT transfection increased sub-G1 fractionation of MDA-MB-231 cells and partially decreased transcription related to S/M-phase entry (Supp. Fig. S3 and S5B). These results indicate that changes induced by CT-treatment could affect the pyrimidine nucleotide biosynthetic pathway and cell cycle-dependent activity in cancer cells. This cellular stress could be caused by the sustained substrate cycle between degradation and synthesis of dTMP, followed by delay of replication, which may lead to cell death by apoptosis. Paradoxically, checkpoint pathway activation has been reported to lead to downregulation of dNTP availability in mammalian cells^56^. Downregulation of Chk1 leads to Mus81/Eme2/ Mre11-dependent DNA damage which, via activation of the ATM pathway, appears to limit dNTPs available for replication and results in slower replication fork progression. Both cases seem to depend on upregulation or changed subcellular localization of the RRM2 but the details of this link are not clear. Taken together, dNTP imbalance by CT could induce both compensation of the lacking nucleotides and blocking miss-incorporation into DNA by activation of SAMHD1. As demonstrated in this study, dTMP selective hydrolysis by CT could trigger cell apoptosis imbalanced from opposing signals between the nucleotide feedback regulation and the decreased DNA instability.

The term ‘undruggable’ refers to ‘difficult to drug’ or ‘yet to be drugged’ but not necessarily ‘impossible to target pharmacologically.’ Although progress is being made to ‘drug’ many of these targets, many difficulties still exist in overcoming obstacles for targeting currently intractable and undruggable oncogenes. As part of this effort to treat “undruggable cancer,” the expanded cancer hallmark was suggested by researchers for finding innovative cancer targets, such as intrinsic metabolic targets, TME targets, the protein phosphorylation network, oncogenic cell states and so on^6,57,58^. Among these expanded cancer hallmarks, the strategy to target metabolic vulnerability could be a promising alternative for so-called ‘undruggable cancers’ when an oncogene is not ideally ‘druggable’. Strategies to target metabolic vulnerability go beyond the study of simply blocking a metabolic pathway or inhibiting biosynthesis by signal transduction and include strategies to induce specific metabolic exhaustion to reprogram cellular metabolism with complementary abilities. In this study, we showed that CT could induce apoptosis in ‘undruggable’ TNBC cells depending on their metabolic vulnerability and suggested a ‘druggable’ treatment based on exploiting metabolic vulnerability.

TNBC cells used in this study have different forms of mutated p53, which is thought to contribute to the adaptation of TNBC cells to tumor-associated stress stimuli. As shown in previous therapies for TNBC, TNBCs are known to be very difficult to treat due to the ambiguity of the TP53 target and the diversity of cancers. Thus, there remain many difficulties in making TP53 a “druggable” target by previous targeted therapy for TNBC. To date, TP53 has been a controversial target in cancer therapy, given the fact that the TP53 can be both cancer-prone and inhibitory. Loss or mutation of the p53 tumor suppressor gene is one of the most common recurrent genetic abnormalities observed in TNBC cells^59^. Indeed, mutated p53 proteins execute their oncogenic gain of function (GOF) by interacting with transcription factors or cofactors to promote or repress gene expression or with proteins irrelevant to transcription^60^. Moreover, mutated p53 can orchestrate stress response mechanisms that facilitate tumor cell survival and adaptation to multiple intrinsic and extrinsic stress conditions^32^. As shown in Fig. 9, the unexpected decrease of mutated p53 was found in all three TNBC cell types treated with CT. This result was somewhat reminiscent of an earlier report where imbalanced nucleotide pools in human normal fibroblast BJ cells, arising as a consequence of DNA damage, triggered p53 activation through the human counterpart to the Mec1-Rad53-Dun1 pathway^61^. However, in this study, CT-induced dNTP pool imbalance in TNBC cells, already up-regulated by mutated p53, led to a decrease of mutated p53. Since the decreased level of p53 in TNBC cells was specific to treatment by CT, the pool imbalance by CT was presumed not to affect the tumor suppression effect of p53 but the decrease of crosstalk between the mutated p53 and the tumor stress response factor.

As discussed earlier, abnormal dNTP pool ratios are associated with increased rates of mutagenesis. Balanced levels of intracellular dNTPs are critical in maintaining the genomic integrity of cells^34,42,43^. In practice, many types of cancer cells with rapid cellular proliferation show enhanced mutagenesis and dysregulation of the cell cycle due to impairment of intracellular dNTP homeostasis^35,62^. It is interesting to consider to what extent the genome integrity checkpoint acts to recognize imbalanced pools and minimize mutagenesis. The checkpoint responded when ≥ 1 of the dNTPs were depleted, but not when all 4 pools were adequate, even if imbalanced^63^. Hence, the consequences of highly unbalanced pools per se did not seem to be sensed as sufficiently damaging to activate the checkpoint. In this study, we elucidated that the supplementation of pyrimidine nucleotides would be highly activated within the range allowed by cellular capacity, if the dTTP present in excess in TNBC cells was selectively and sharply decreased. However, we also expected that TNBC cells would not sense the dNTP imbalance as sufficiently damaging to activate the checkpoint, unless a certain dNTP were completely deficient. Indeed, the transcripts involved in DNA replication and repair of misincorporated nucleotides appeared to be reduced overall. This observation alone could not prove whether the damage to the DNA was severe, but it did show that cellular activity misincorporating nucleotides into DNA was at least reduced. Recently, efforts have focused on treating refractory cancers by altering the dNTP pool through metabolic reprogramming along with efforts to mitigate the risk of DNA mutations^43,61,63–65^. Although many researchers have considered nucleotide metabolism as a potential promising therapeutic target, to date, the only proven examples involve inhibition of a biosynthetic pathway by enzyme inhibitors like metabolite analogues and by the oncogenes related to nucleotide regulation^66–68^.

A plethora of studies have shown that oncogene-driven cancers exhibit higher levels of activation of the pyrimidine de novo pathway, indicating that the inhibition of pyrimidine biosynthetic enzymes could be a promising opportunity for cancer therapy. In recent years, DHODH has emerged as a promising synthetic lethal target for various oncogenic events, including BRAF (V600E) mutation, PTEN deficiency, and RAS mutation^69–71^. Since oncogene-driven cancers show high complexity and diversity in genetic backgrounds, the best therapy must consider the genetic features of each. In this study, we suggest targeting pyrimidine metabolism vulnerability as an innovative strategy for treatment of various oncogene-driven cancers, in agreement with other researchers. However, instead of using a direct inhibitor to block the pyrimidine de novo pathway, we demonstrated a novel approach to promote continuous TNBC cell consumption of resources for pyrimidine synthesis by inducing nucleotide imbalance. Also, we used mRNA-encoded CT capable of selective hydrolysis of dTMP to thymidine to minimize mutagenesis caused by nucleotide imbalance. Because additional nucleotide deficiencies can activate excessive repair mechanisms for misincorporation into DNA due to uncontrolled replication, increasing dTTP in relation to other nucleotides could be the best strategy for consuming resources of pyrimidines. As described in this study, dTMP-specific CT showed more effective synthetic lethality on TNBC cells, depending upon metabolic flexibility and vulnerability for supplying the nucleotide pool, compared to less selective enzymes. Since overcoming oxidative stress is a critical step for tumor progression, NADPH plays a key role in cellular anti-oxidation systems in cancer cells by providing reducing equivalents to generate reduced forms of antioxidant molecules, which are highly correlated with biological behaviors in cancer cell^72,73^. Thus, the decrease of glutathione and NADPH derived from overconsuming glutamate, could generate excess ROS. The decrease of NADPH/NADP that induces apoptosis by dysregulating redox homeostasis in tumor progression-adapted cells may be one of the important MOAs of CT treatment.

Since pyrimidine is a basic and indispensable substrate for nucleic acids and other substances, this dependence on the pyrimidine pathway leads to the synthetic lethal target of pyrimidine synthesis in cancer cells, suggesting hardwired metabolic vulnerabilities in different gain-of-function mutant cancers^74,75^. At this time, this metabolic vulnerability is mainly caused by crosstalk between the pyrimidine pathway and other metabolic signaling, contributing to cancer complexity and diversity. Thus we need a greater understanding of metabolic heterogeneity and complexity of gain-of-function mutant cancers for effective clinical therapy. Importantly, increasing evidence has demonstrated that dNTP imbalance closely correlates with various oncogenic signaling pathways and redox homeostasis in many cancers, as discussed above. It is worth noting that classical one-size-fits-all treatment may fail to achieve satisfactory clinical benefits due to heterogeneous and flexible cancer metabolism. Metabolic reprogramming by CT could be an innovative alternative cancer treatment approach that facilitates tumor suppression by targeting metabolic vulnerability. This has promise to pave the way for new cancer treatments, including the development of drugs not currently considered in traditional oncology pipelines.

## Supplementary Information

Supplementary Information.

## Data Availability

All data generated or analyzed during this study are included in this published article (and its Supplementary Information files). The raw RNA-Seq fastq data discussed in this publication have been deposited in NCBI’s Gene Expression Omnibus and are accessible through GEO Series accession number GSE201421 (https://www.ncbi.nlm.nih.gov/geo/query/acc.cgi?acc=GSE201421).
