# Supplementary material for: dTMP imbalance through thymidylate 5′-phosphohydrolase activity induces apoptosis in triple-negative breast cancers
Source: Sci Rep. 2022 Nov 21;12:20027. doi: 10.1038/s41598-022-24706-4 (PMC9681768; doi:10.1038/s41598-022-24706-4)
Supplement: Supplementary file 1 — Supplementary Information. [file 41598_2022_24706_MOESM1_ESM.pdf]

# dTMP imbalance through thymidylate 5’-phosphohydrolase activity induces apoptosis in triple-negative breast cancers

Dae-Ho Kim[1,2,3], Jin-Sook Kim[1], Chang-Soo Mok[1,4], En-Hyung Chang[1], Jiwon Choi[1], Junsub Lim[1], Chul-Ho Kim[3], Ah-Reum Park[5], Yu-Jeong Bae[5], Bong-Seong Koo[1]* and Hyeon-Cheol Lee[1]*

These authors contributed equally: Dae-Ho Kim, Jin-Sook Kim

**A**

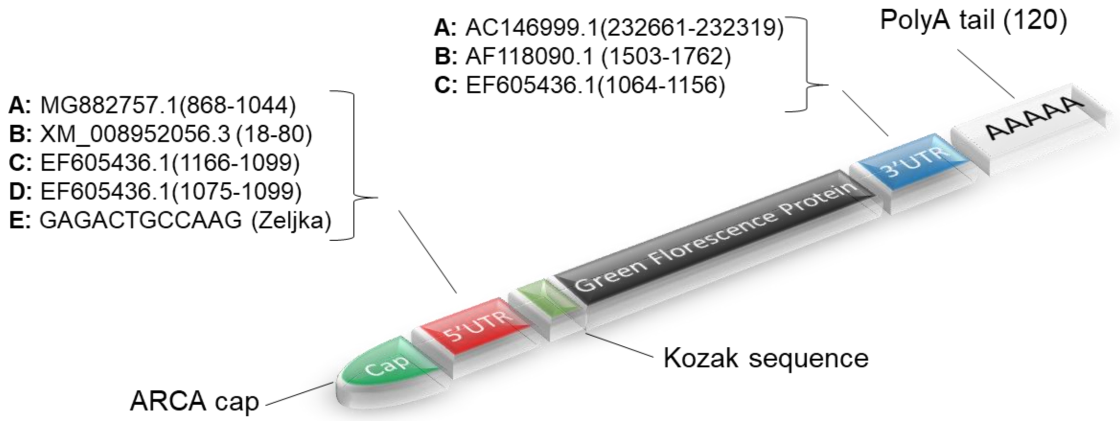


| ID | 5'UTR/3'UTR |
|---|---|
| 1 | A/A |
| 2 | A/- |
| 3 | A/B<br>(short A tail 64) |
| 4 | A/B |
| 5 | B/B |
| 6 | C/C |
| 7 | D/C |
| 8 | E/C |
| 9 | -/C |

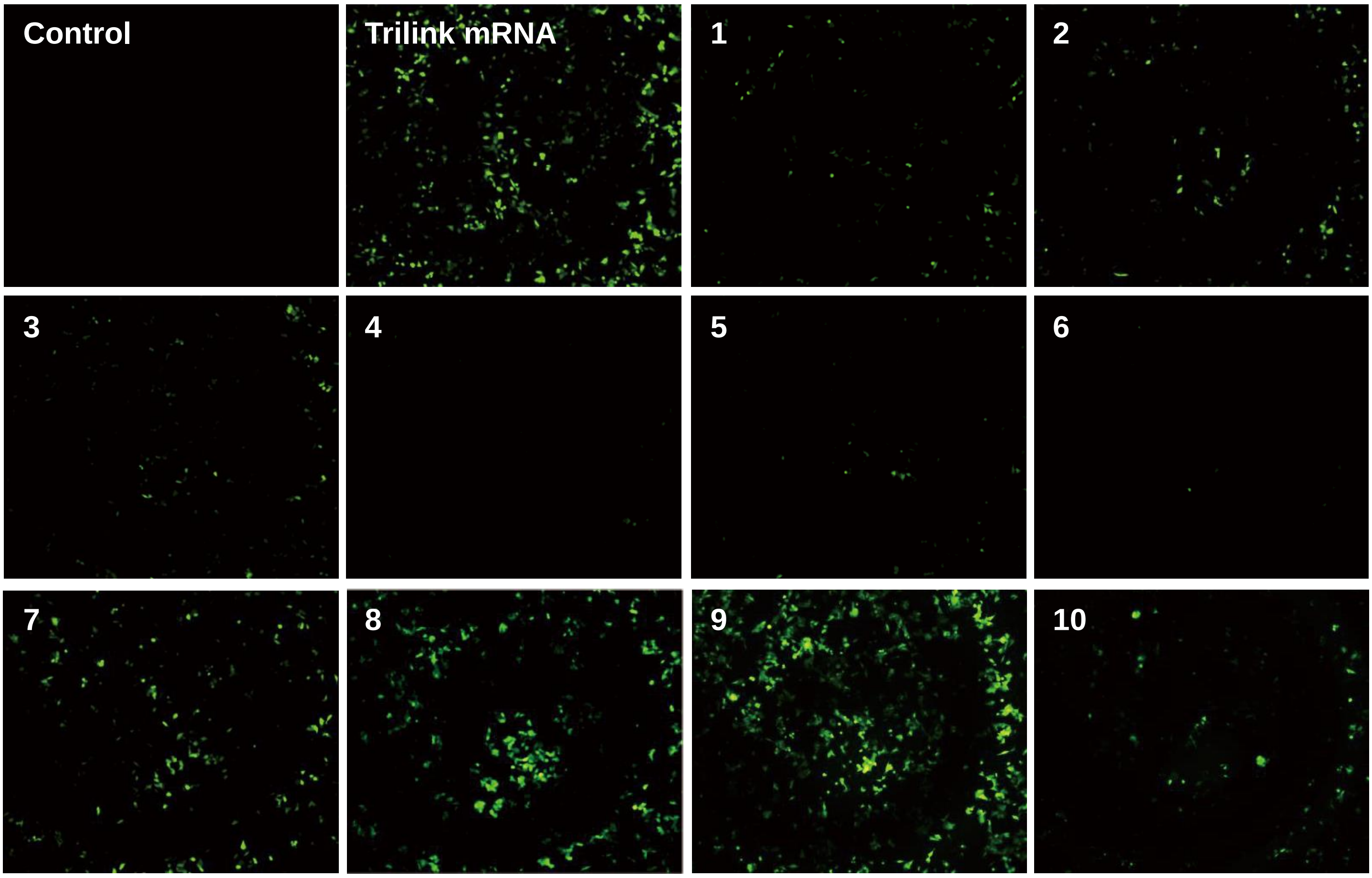


**Supplementary Figure S1 (continued). IVT mRNA construct optimization.** To optimize IVT construct, we evaluated combinatory IVT mRNAs constructed with five different 5'UTRs and three different 3'UTR. ARCA cap and poly A tail were commonly added after each IVT reaction. Positive control, Trilink mRNA was manufactured by Trilink co ltd and had the same structure to our constructs except Clean cap$^{TM}$ and 5'/3' UTR. (A) Structure of EGFP mRNAs and images of EGFP mRNAs by fluorescence microscopy. (B) Evaluation of EGFP transfection efficiency by flow cytometry.

**B**

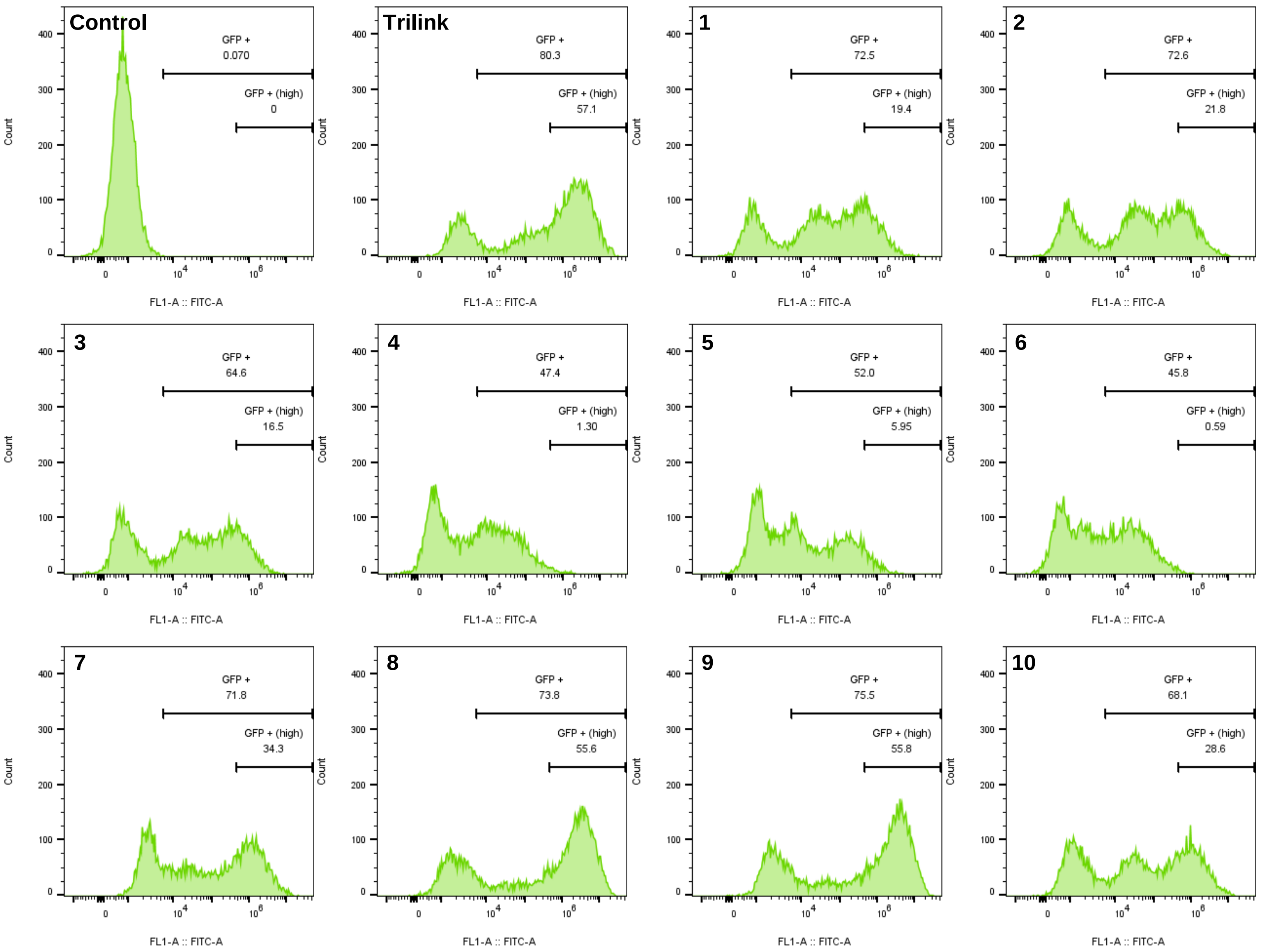


| mRNA | con | Trilink | 1 | 2 | 3 | 4 | 5 | 6 | 7 | 8 | 9 | 10 |
|---|---|---|---|---|---|---|---|---|---|---|---|---|
| GFP-positive (high) cells | 0 | 57.1 | 19.4 | 21.8 | 16.5 | 1.30 | 5.95 | 0.59 | 34.3 | 55.6 | 55.8 | 28.6 |

* GFP+ (high) : Higher 50% of GFP fluorescent region

**Supplementary Figure S1 (continued). IVT mRNA construct optimization.** To optimize IVT construct, we evaluated combinatory IVT mRNAs constructed with five different 5'UTRs and three different 3'UTR. ARCA cap and poly A tail were commonly added after each IVT reaction. Positive control, Trilink mRNA was manufactured by Trilink co ltd and had the same structure to our constructs except Clean cap$^{TM}$ and 5'/3' UTR. (A) Structure of EGFP mRNAs and images of EGFP mRNAs by fluorescence microscopy. (B) Evaluation of EGFP transfection efficiency by flow cytometry.

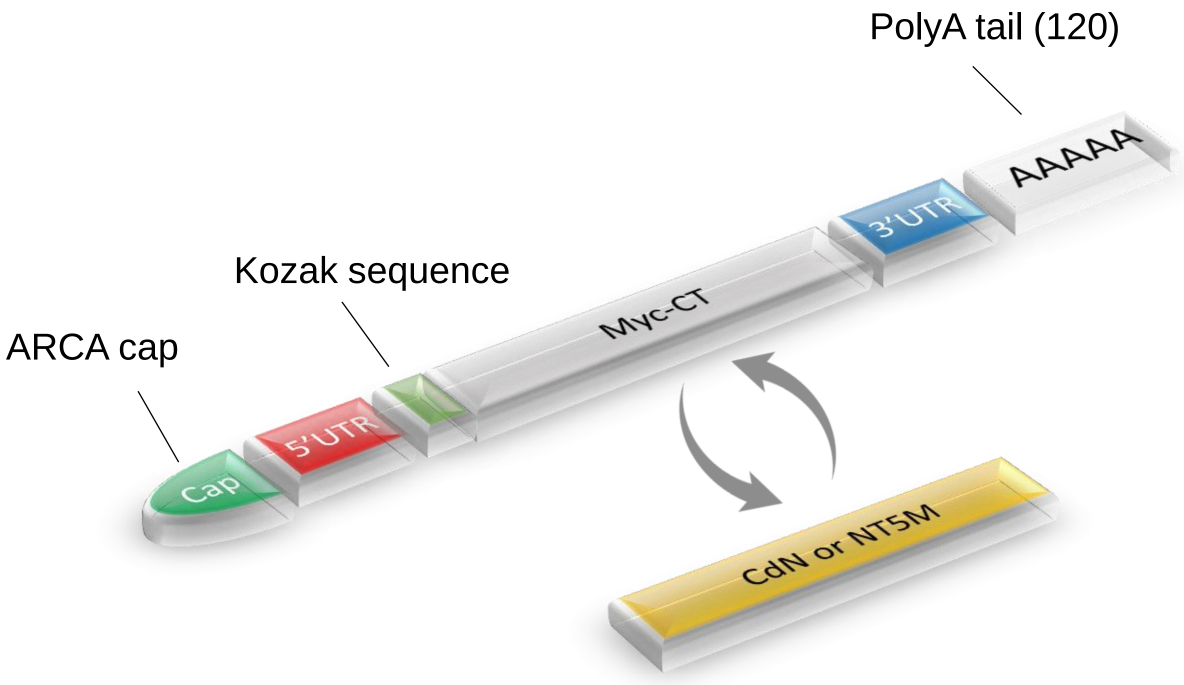


- CdN: 5'-nucleotidase, human cytosolic (NM_001252377)
- NT5M: 5'-nucleotidase, human mitochondrial (NM_020201), signal sequence deleted
- CT : PBS2 5’-nucleotidase

| 5’ NT | Alias | Preferred substrate (Km) | References |
|---|---|---|---|
| PBS2 TMP phosphohydrolase | T001 | dTMP (0.01 mM)<br>dGMP (0.7 mM)<br>dUMP (0.8 mM) | Methods Enzymol. 51:285–290 (1978),<br>Also In-house data |
| Human mitochondrial 5’doxyribonucleotidase | NT5M (hdNT-2) | dGMP (0.09 mM)<br>dUMP (0.16 mM)<br>dTMP (0.3 mM) | Biochem. 46:13809-13818 (2007)<br>Biochemical Pharmacol. 66: 471–479 (2003) |
| Human cytosolic 5’deoxyribonucleotidase | CdN (hdNT-1) | dUMP (1.5 mM)<br>dTMP (1.5 mM)<br>dAMP (3.0 mM)<br>dGMP (3.3 mM) | J. Biol. Chem. 265:6589-6595 (1990)<br>Biochemical Pharmacol. 66: 471–479 (2003) |
| Murine cytosolic 5’deoxyribnucleotidase | mdNT-1 | dUMP (0.8 mM)<br>dAMP (1.0mM)<br>dGMP (1.2mM)<br>dTMP (1.4 mM) | J. Biol. Chem. 275:5409-5415 (1990)<br>Biochemical Pharmacol. 66: 471–479 (2003) |

**Supplementary Figure S2. Construction of 5’nucleotidase IVTs with different dNMP specificity.** To investigate the difference among dNMP specificity of 5’-nucleotidases, additional two mRNA constructs of human cytosolic 5’-nucleotidase (CdN) and human mitochondrial 5’-nucleotide (signal-sequence deleted) (NT5M) which were known to have different substrate preference, were created by replacing CT gene. The table shows the nucleotide substrate preference of each 5’-nucleotidase as representative Km values.

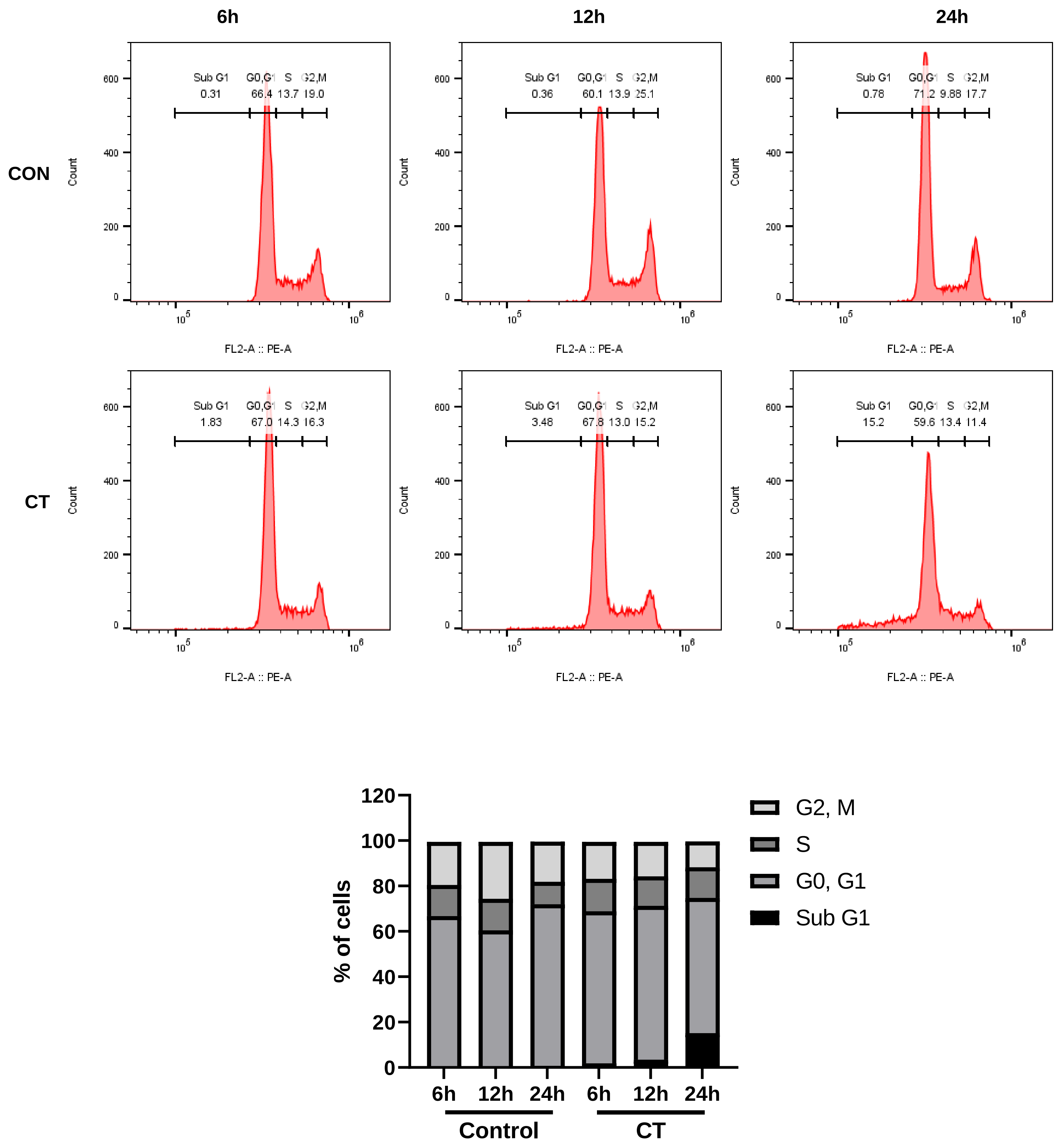


**Supplementary Figure S3. Time-dependent cell cycle analysis using flow cytometer.** MDA-MB-231 cells were stained with PI on 6h, 12h, and 24h after transfection. DNA content was analyzed by flow cytometry. The percentages of cells in Sub G1, G0/G1, S, and G2/M phase were described by histogram plots and graph.

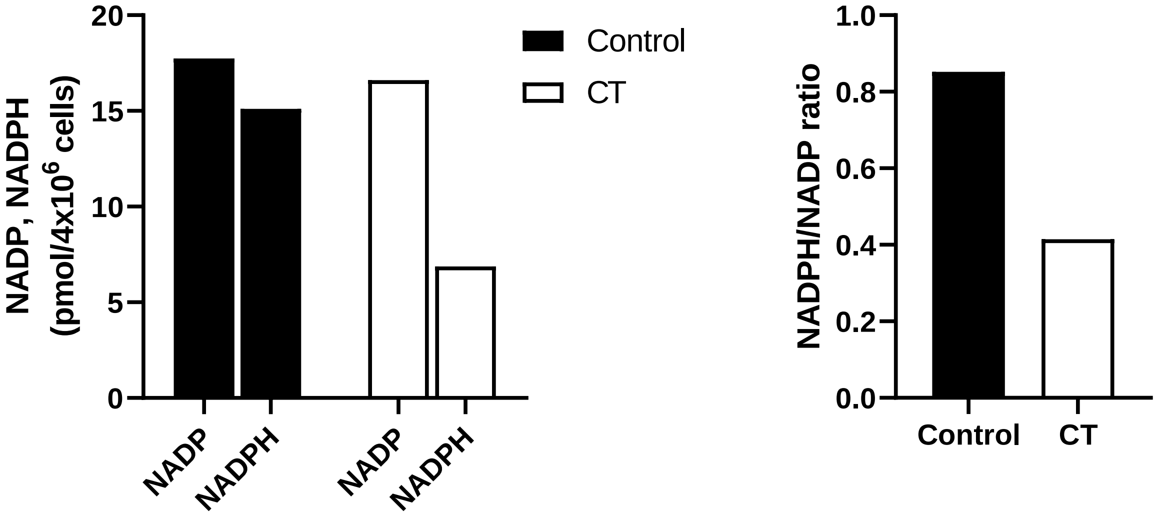


**Supplementary Figure S4. Effect of CT on cellular NADPH/NADP$^+$ ratios in MDA-MB-231 cells.** The metabolic shift of CT expression in MDA-MB-231 significantly decreased the NADP/NADPH ratio, which could decrease GSH/GSSG ratio resulting in excess ROS production.

A

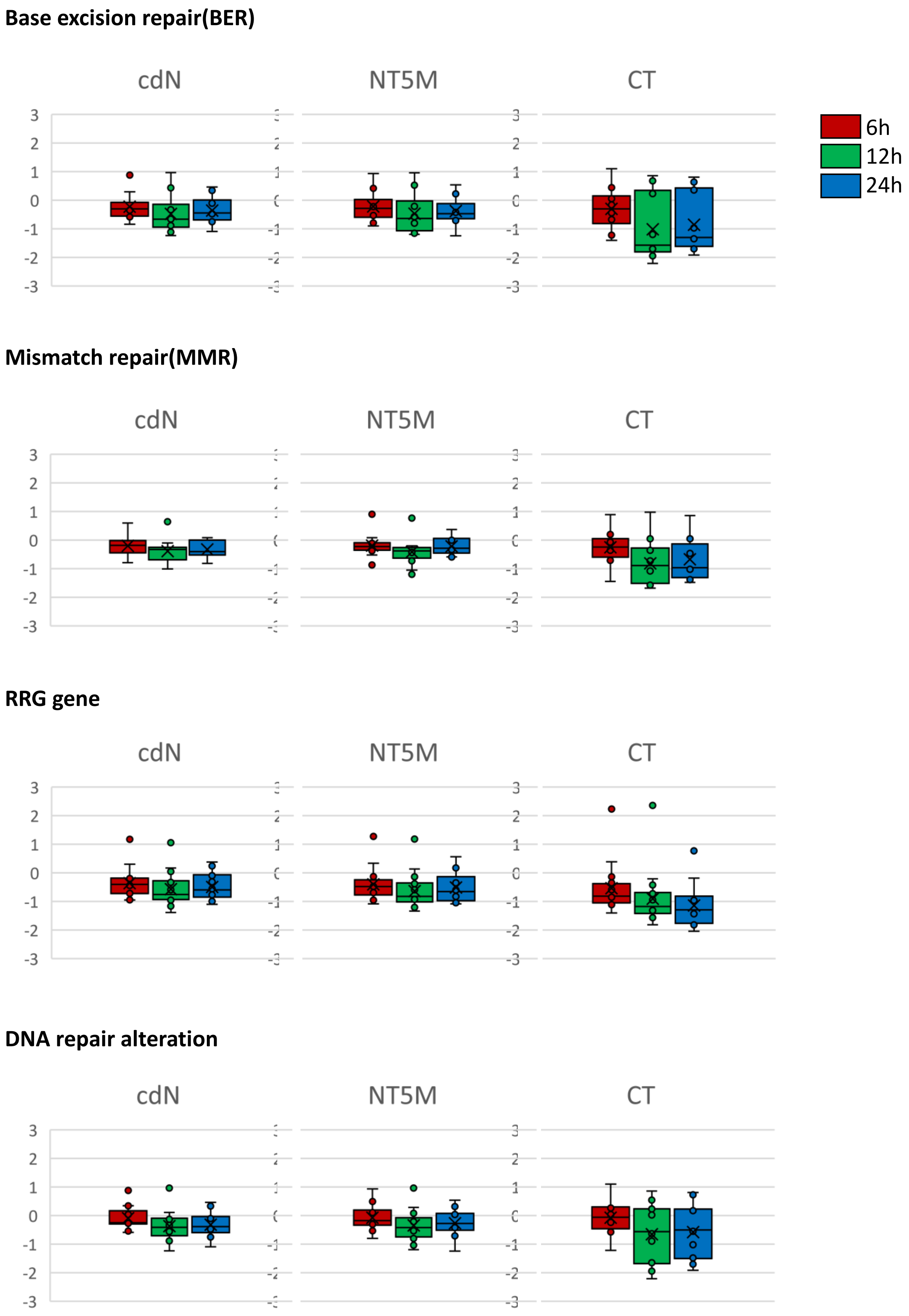


**Supplementary Figure S5. Time-course profiles of Replication-Related Genes (RRG) and DNA repair (BER/MMR) genes in MDA-MB-231.** (A) Average expression of BER, MMR, RRG genes and DNA repair alteration. (B) Average expression of replication-related genes (S-phase and M-phase). All Gene lists are derived from KEGG and shown in Fig. 6.

**B**

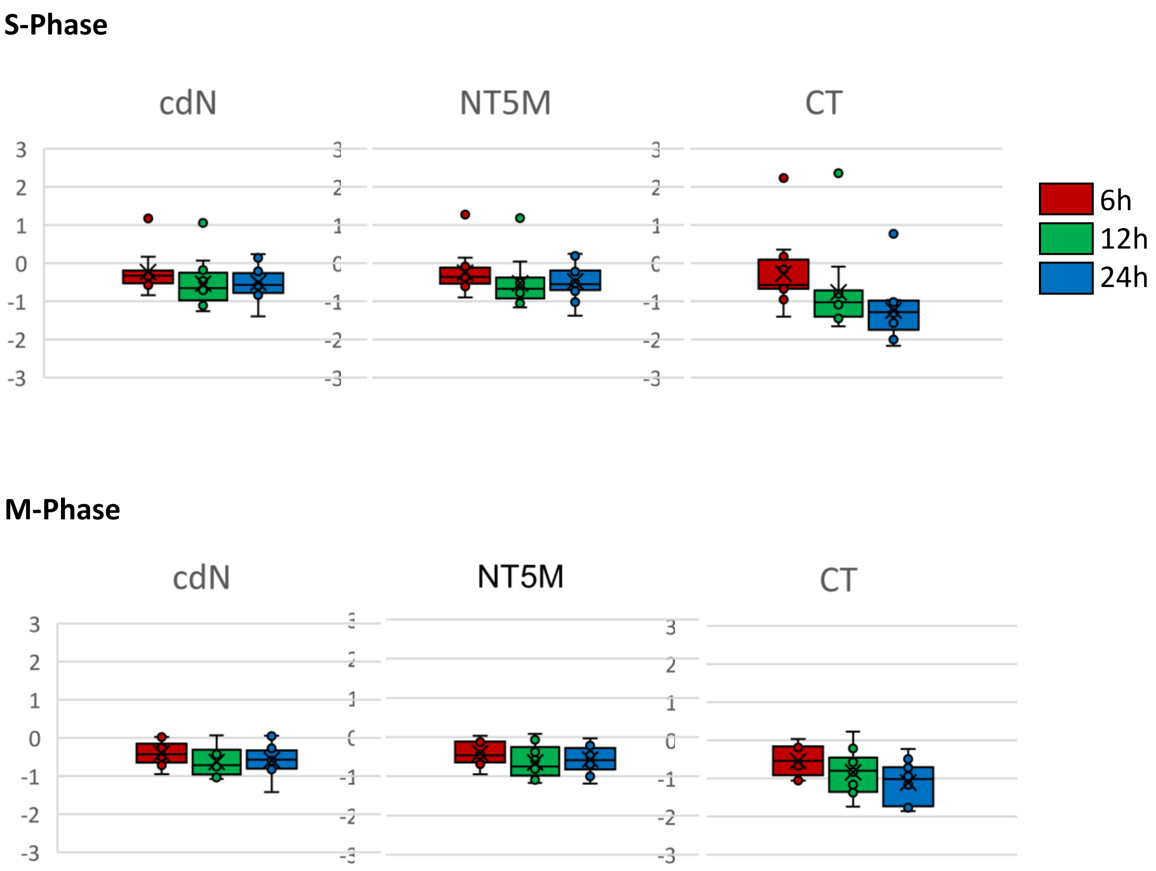


**Supplementary Figure S5 (Continued). Time-course profiles of Replication-Related Genes (RRG) and DNA repair (BER/MMR) genes in MDA-MB-231.** (A) Average expression of BER, MMR, RRG genes and DNA repair alteration. (B) Average expression of replication-related genes (S-phase and M-phase). All Gene lists are derived from KEGG and shown in Fig. 6.

**Supplementary Table S1.** Representative genes showing significant inversion of transcription pattern in RNAseq analysis of MDA-MB-231. In cancer cells, the following representative genes known to help tumor progression and suppression, appeared to be completely inverted in their transcription by CT transfection.

| Classification | Gene | Full name | CT response | Feature related to role in cancer | Ref |
|---|---|---|---|---|---|
| **S-Phase RRG** | **CCDC6** | Coiled-coil domain containing 6 | **UP** | The encoded protein is ubiquitously expressed and may function as a tumor suppressor. | Merolla et al[1]. |
| **BER** | **MBD1** | Methyl-CpG Binding Domain Protein 1 | **UP** | Up-regulation of MBD1 promotes pancreatic cancer cell epithelial-mesenchymal transition and invasion by epigenetic down-regulation of E-cadherin (Tumor suppressor) | Xu et al[2] |
| **BER** | **TDG** | Thymine DNA Glycosylase | **UP** | TDG is a novel tumor suppressor of liver malignancies | Hassan et al[3] |
| **MMR** | **LIG4** | DNA ligase 4 | **UP** | In TNBC cells, compared to nonbasal breast cancer, LIG4 is frequently amplified, and an increased gene dose is associated with higher Lig4 expression. LIG4 Prevents replication fork stalling and promotes cellular proliferation in TNBC | Joshi et al[4] |
| **DNA repair alteration** | **MDM4** | Regulator of P53 | **UP** | MDM2/4 proteins bind the p53 tumor suppressor protein and inhibit its activity, and have been shown to be overexpressed in a variety of human cancers. | Toufektchan et al[5] |
| **DNA repair alteration** | **POLB** | DNA polymerase β | **UP** | DNA polymerase β deficiency is linked to aggressive breast cancer. POLB may be a tumor suppressor and involved in breast cancer pathogenesis. | Abdel-Fatah et al[6] |
| **S-Phase BER** | **PCNA** | Proliferating cell nuclear antigen | **DOWN** | Tumor cells express high levels of PCNA, identifying it as a potentially ideal target for cancer therapy. | Dillehay et al[7] |
| **S-Phase** | **FEN1** | Flap endonuclease 1 (FEN1) | **DOWN** | FEN1 participates in various DNA repair pathways and contributes to cancer progression and drug resistance in chemotherapy. | Lu et al[8] |
| **M-Phase RRG** | **CDC20** | cell division cycle 20 | **DOWN** | CDC20 overexpression has been associated with inappropriately functioning SAC and aneuploidization in oral cancer. High CDC20 expression has been reported in several human cancer cell lines and several carcinoma tissues | Karra et al[9] |
| **RRG** | **CDC25A/B** | Cell Division Cycle 25A/25B | **DOWN** | CDC25A/B is a critical regulator of cell cycle progression and checkpoint response. CDC25A/B overexpression are frequently found in many cancers, and are often associated with high-grade tumors and poor prognosis. | Ray et al[10]<br>Boutros et al[11] |
| **RRG** | **PLK1** | Polo-like kinase 1 | **DOWN** | Inhibition of PLK1 could lead to death of cancer cells by interfering with multiple stages of mitosis. | Liu et al[12] |
| **DNA repair alteration** | **PARP1** | poly(ADP-ribose) polymerase 1 | **DOWN** | PARP1 gene expression profile in surgical samples from more than 8,000 primary malignant and normal human tissues. PARP1 expression was found to be significantly increased in several malignant tissues from patients with breast cancers | Ossovskaya et al[13] |

## References


1 Merolla, F. *et al.* Involvement of H4(D10S170) protein in ATM-dependent response to DNA damage. *Oncogene* **26**, 6167-6175, doi:10.1038/sj.onc.1210446 (2007).

2 Xu, J. *et al.* Up-regulation of MBD1 promotes pancreatic cancer cell epithelial-mesenchymal transition and invasion by epigenetic down-regulation of E-cadherin. *Current molecular medicine* **13**, 387-400 (2013).

3 Hassan, H. M., Isovic, M., Underhill, M. T. & Torchia, J. TDG is a novel tumor suppressor of liver malignancies. *Molecular & cellular oncology* **7**, 1768819, doi:10.1080/23723556.2020.1768819 (2020).

4 Joshi, R. R., Ali, S. I. & Ashley, A. K. DNA Ligase IV Prevents Replication Fork Stalling and Promotes Cellular Proliferation in Triple Negative Breast Cancer. *Journal of nucleic acids* **2019**, 9170341, doi:10.1155/2019/9170341 (2019).

5 Toufektchan, E. *et al.* Germline mutation of MDM4, a major p53 regulator, in a familial syndrome of defective telomere maintenance. *Sci Adv* **6**, eaay3511-eaay3511, doi:10.1126/sciadv.aay3511 (2020).

6 Abdel-Fatah, T. M. A. *et al.* DNA polymerase β deficiency is linked to aggressive breast cancer: A comprehensive analysis of gene copy number, mRNA and protein expression in multiple cohorts. *Molecular Oncology* **8**, 520-532, doi:https://doi.org/10.1016/j.molonc.2014.01.001 (2014).

7 Dillehay, K. L., Lu, S. & Dong, Z. Antitumor effects of a novel small molecule targeting PCNA chromatin association in prostate cancer. *Molecular cancer therapeutics* **13**, 2817-2826, doi:10.1158/1535-7163.Mct-14-0522 (2014).

8 Lu, X. *et al.* MicroRNA-140 impedes DNA repair by targeting FEN1 and enhances chemotherapeutic response in breast cancer. *Oncogene* **39**, 234-247, doi:10.1038/s41388-019-0986-0 (2020).

9 Karra, H. *et al.* Cdc20 and securin overexpression predict short-term breast cancer survival. *Br J Cancer* **110**, 2905-2913, doi:10.1038/bjc.2014.252 (2014).

10 Ray, D. & Kiyokawa, H. CDC25A phosphatase: a rate-limiting oncogene that determines genomic stability. *Cancer Res* **68**, 1251-1253, doi:10.1158/0008-5472.Can-07-5983 (2008).

11 Boutros, R., Lobjois, V. & Ducommun, B. CDC25 phosphatases in cancer cells: key players? Good targets? *Nat Rev Cancer* **7**, 495-507, doi:10.1038/nrc2169 (2007).

12 Liu, Z., Sun, Q. & Wang, X. PLK1, A Potential Target for Cancer Therapy. *Translational oncology* **10**, 22-32, doi:10.1016/j.tranon.2016.10.003 (2017).

13 Ossovskaya, V., Koo, I. C., Kaldjian, E. P., Alvares, C. & Sherman, B. M. Upregulation of Poly (ADP-Ribose) Polymerase-1 (PARP1) in Triple-Negative Breast Cancer and Other Primary Human Tumor Types. *Genes Cancer* **1**, 812-821, doi:10.1177/1947601910383418 (2010).

# Figure Raw data

**Fig. 1B.**

| Apoptotic cells | 1 | 2 | 3 |
|---|---|---|---|
| **0** | 5 | 6.69 | 5.17 |
| **0.625** | 10.71 | 8.71 | 7.17 |
| **1.25** | 22.4 | 15.69 | 14.5 |
| **2.5** | 59.24 | 49.57 | 41.48 |

**Fig. 1C.**

| Apoptotic cells | Control | | | CT | | |
|---|---|---|---|---|---|---|
| | **1** | **2** | **3** | **1** | **2** | **3** |
| **6h** | 4.87 | 5.07 | 5.19 | 8.28 | 8.14 | 8.61 |
| **12h** | 4.71 | 4.93 | 5.4 | 11.94 | 14.266 | 13.31 |
| **24h** | 5.85 | 7.86 | 6.97 | 23.13 | 23.93 | 23.8 |

**Fig. 1D.**

***Concentration dependent***

γH2AX

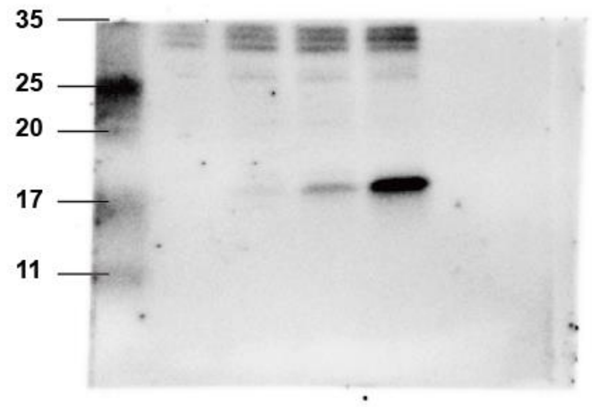


PARP

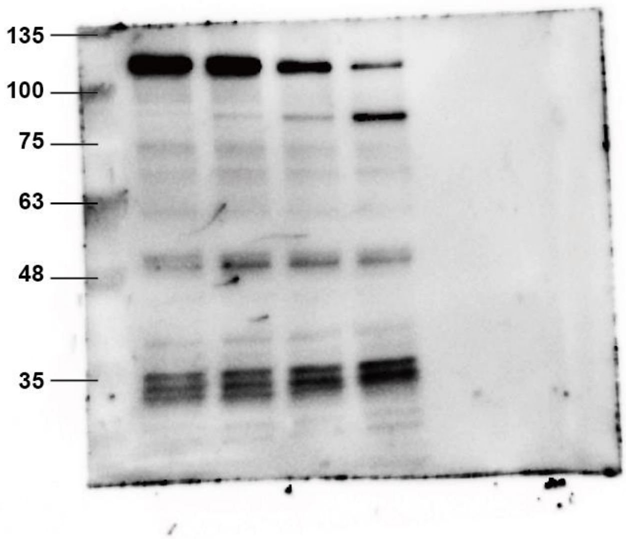


GAPDH

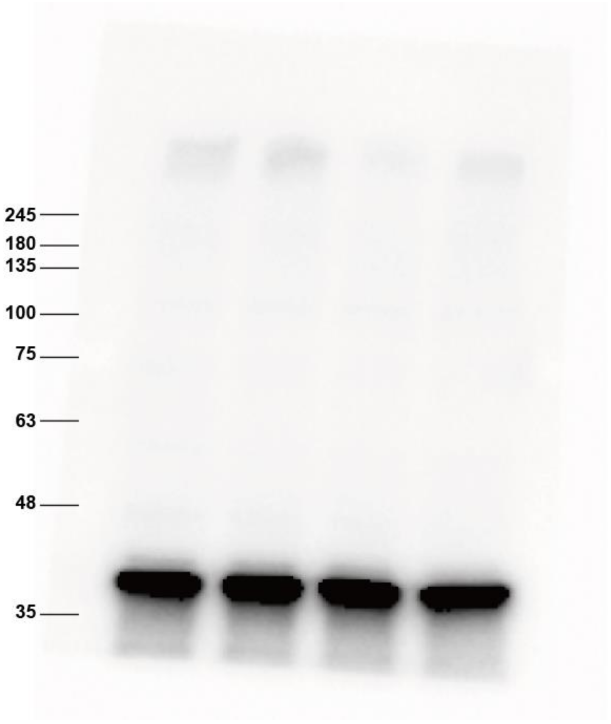


CT-myc (marker merged)

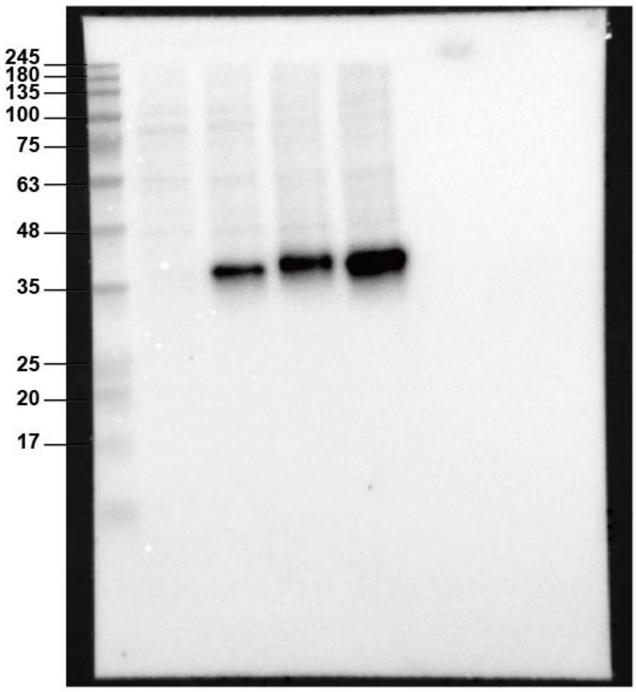

***Time dependent***

γH2AX (marker merged)

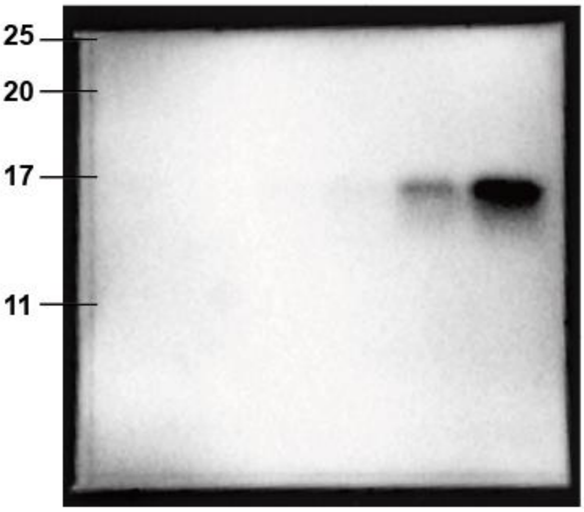


PARP (marker merged)

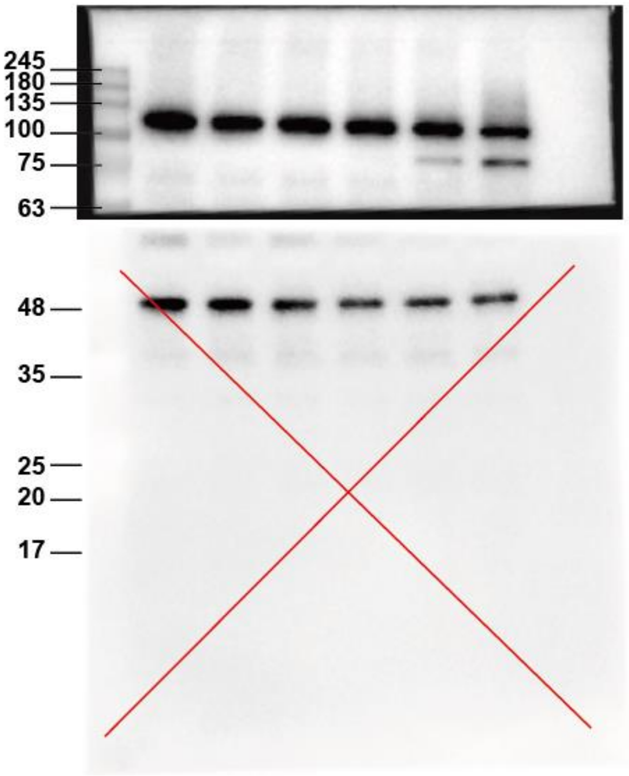


GAPDH (marker merged)

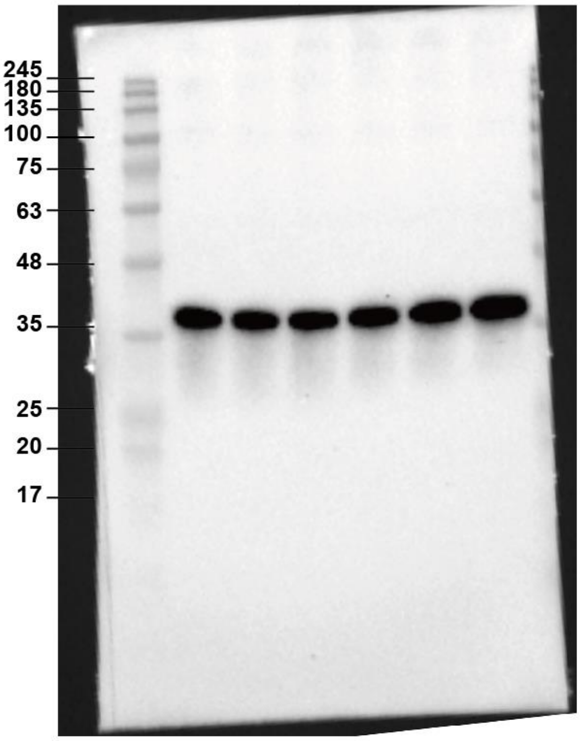


CT-myc (marker merged)

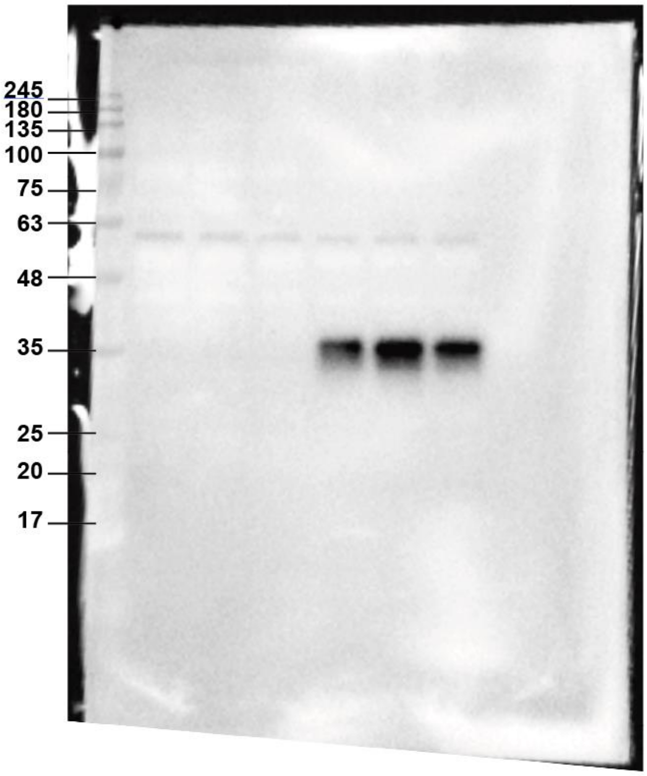

**Fig. 2.**

| dNTPs | dTTP | | | dCTP | | | dATP | | | dGTP | | |
|---|---|---|---|---|---|---|---|---|---|---|---|---|
| | **1** | **2** | **3** | **1** | **2** | **3** | **1** | **2** | **3** | **1** | **2** | **3** |
| **Control** | 13.6 | 14.1 | 15.6 | 2.1 | 2.6 | 3.6 | 2 | 1.6 | 1.6 | 1.7 | 2.4 | 3 |
| **CT** | 6.1 | 7.2 | 7.9 | 2.9 | 3.5 | 4.1 | 1.2 | 1.4 | 1.3 | 0.7 | 1.3 | 1.7 |

**Fig. 3A.**

| Gene _ID | Transcript_ID | Gene_ Symbol | Description | gene_ biotype | Protein_ID | 6hr-CON_Read_Count | 6hr-CT_Read_Count | 12hr-CON_Read_Count | 12hr-CT_Read_Count | 24hr-CON_Read_Count | 24hr-CT_Read_Count |
|---|---|---|---|---|---|---|---|---|---|---|---|
| 790 | NM_001306079,NM_004341, XM_006712101,XM_024453131 | CAD | carbamoyl-phosphate synthetase 2, aspartate transcarbamylase, and dihydroorotase | protein_ coding | NP_001293008.1; NP_004332.2; XP_006712164.1;XP_024308899.1 | 17002.0 | 9296.2 | 13103.0 | 7840.6 | 10181.0 | 16301.2 |
| 1723 | NM_001361, XM_005255827, XM_005255829, XM_017022990 | DHODH | dihydroorotate dehydrogenase (quinone) | protein_ coding | NP_001352.2; XP_005255884.1; XP_005255886.1; XP_016878479.1 | 774.0 | 879.3 | 620.0 | 557.6 | 504.0 | 1165.2 |
| 7372 | NM_000373, NR_033434, NR_033437, XR_001740253 | UMPS | uridine monophosphate synthetase | protein_ coding | NP_000364.1 | 2236.0 | 3525.6 | 1880.0 | 3358.4 | 1479.0 | 3514.2 |
| 6240 | NM_001033, NM_001318064, NM_001318065, NM_001330193 | RRM1 | ribonucleotide reductase catalytic subunit M1 | protein_ coding | NP_001024.1; NP_001304993.1; NP_001304994.1; NP_001317122.1 | 17998.0 | 18562.5 | 14158.0 | 13892.6 | 10896.0 | 9086.1 |
| 6241 | NM_001034, NM_001165931, NR_164157 | RRM2 | ribonucleotide reductase regulatory subunit M2 | protein_ coding | NP_001025.1; NP_001159403.1 | 29004.0 | 31000.1 | 20543.0 | 16543.3 | 13812.0 | 8671.6 |
| 50484 | NM_001172477, NM_001172478, NM_015713 | RRM2B | ribonucleotide reductase regulatory TP53 inducible subunit M2B | protein_ coding | NP_001165948.1; NP_001165949.1; NP_056528.2 | 2091.0 | 6658.4 | 2054.0 | 8209.4 | 2251.0 | 5123.8 |
| 1890 | NM_001113755, NM_001113756, NM_001257988, NM_001257989, NM_001953 | TYMP | thymidine phosphorylase | protein_ coding | NP_001107227.1; NP_001107228.1; NP_001244917.1; NP_001244918.1; NP_001944.1 | 279.0 | 875.0 | 156.0 | 2359.0 | 188.0 | 3749.5 |
| 7298 | NM_001071, NM_001354867, NM_001354868, XM_024451242 | TYMS | thymidylate synthetase | protein_ coding | NP_001062.1; NP_001341796.1; NP_001341797.1; XP_024307010.1 | 19125.0 | 20504.5 | 14363.0 | 12176.9 | 10381.0 | 6318.8 |
| 7083 | NM_001346663, NM_001363848, NM_003258 | TK1 | thymidine kinase 1 | protein_ coding | NP_001333592.1; NP_001350777.1; NP_003249.3 | 10351.0 | 11438.8 | 8050.0 | 9856.5 | 6883.0 | 6412.2 |
| 1841 | NM_001165031, NM_001320902, NM_001320903, NM_001320904, NM_001320905, NM_012145, NR_033255, NR_135492 | DTYMK | deoxythymidylate kinase | protein_ coding | NP_001158503.1; NP_001307831.1; NP_001307832.1; NP_001307833.1; NP_001307834.1; NP_036277.2 | 3838.0 | 3713.4 | 3093.0 | 2281.8 | 2324.0 | 2546.9 |
| 25939 | NM_001363729, NM_001363733, NM_015474 | SAMHD1 | SAM and HD domain containing deoxynucleoside triphosphate triphosphohydrolase 1 | protein_ coding | NP_001350658.1; NP_001350662.1; NP_056289.2 | 6080.0 | 26023.3 | 6024.0 | 16050.0 | 5447.0 | 9425.9 |
| 1854 | NM_001025248, NM_001025249, NM_001330286, NM_001948, XM_017021988, XR_001751125, XR_931760 | DUT | deoxyuridine triphosphatase | protein_ coding | NP_001020419.1; NP_001020420.1; NP_001317215.1; NP_001939.1; XP_016877477.1 | 5422.0 | 4981.0 | 3542.0 | 3650.1 | 3214.0 | 2349.0 |
| 2805 | NM_002079 | GOT1 | glutamic-oxaloacetic transaminase 1 | protein_ coding | NP_002070.1 | 5048.0 | 11199.8 | 4558.0 | 24229.4 | 3562.0 | 35877.6 |
| 4831 | NM_001018137, NM_001018138, NM_001018139, NM_001198682, NM_002512 | NME2 | NME/NM23 nucleoside diphosphate kinase 2 | protein_ coding | NP_001018147.1; NP_001018148.1; NP_001018149.1; NP_001185611.1; NP_002503.1 | 20086.0 | 17892.4 | 14528.0 | 14600.3 | 12915.0 | 15927.8 |
| 2746 | NM_001318900, NM_001318901, NM_001318902, NM_001318904, NM_001318905, NM_001318906, NM_005271 | GLUD1 | glutamate dehydrogenase 1 | protein_ coding | NP_001305829.1; NP_001305830.1; NP_001305831.1; NP_001305833.1; NP_001305834.1; NP_001305835.1; NP_005262.1 | 15007.0 | 16735.7 | 11690.0 | 14373.0 | 10304.0 | 25114.7 |
| 2752 | NM_001033044, NM_001033056, NM_002065, XM_006711278 | GLUL | glutamate-ammonia ligase | protein_ coding | NP_001028216.1; NP_001028228.1; NP_002056.2; XP_006711341.1 | 5867.0 | 12902.8 | 5325.0 | 11640.8 | 4952.0 | 13929.8 |
| 60 | NM_001101 | ACTB | actin beta | protein_ coding | NP_001092.1 | 319280.0 | 319280.0 | 248119.0 | 248119.0 | 234588.0 | 234588.0 |

**Fig. 3B.**

p-CAD (S1859)
(marker merged)

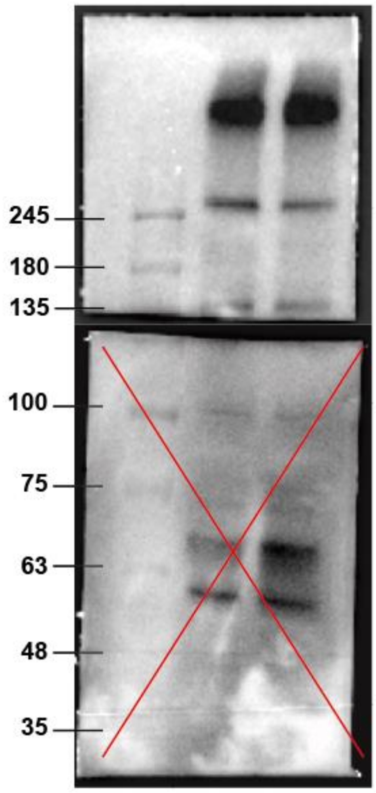


p-CAD (T456)
(marker merged)

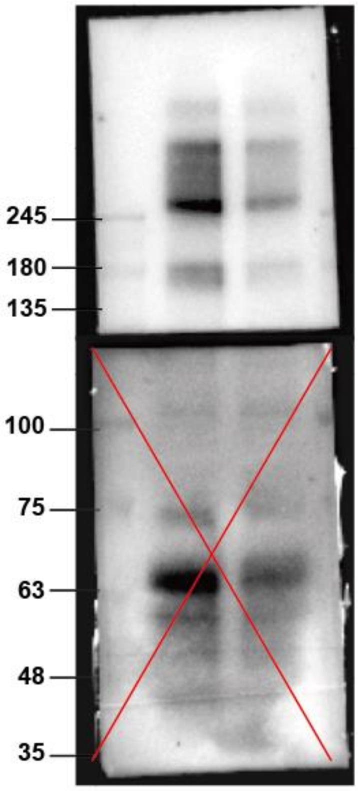


CAD (marker merged)

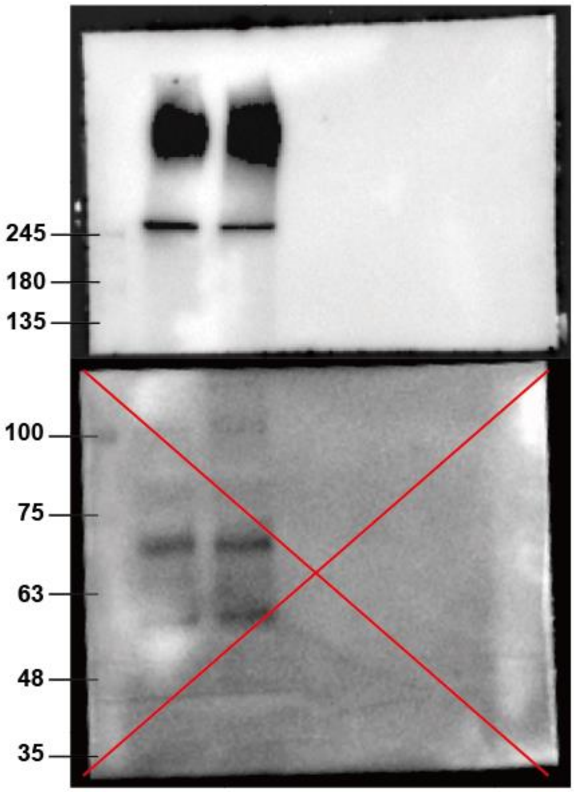


p-p70S6K1 (T389) (marker merged)

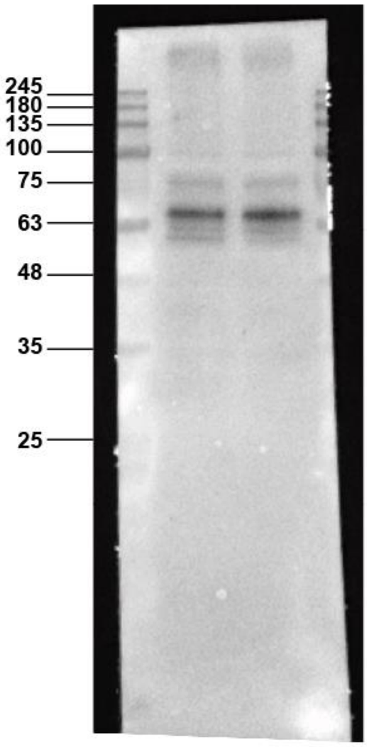


p70S6K1 (marker merged)

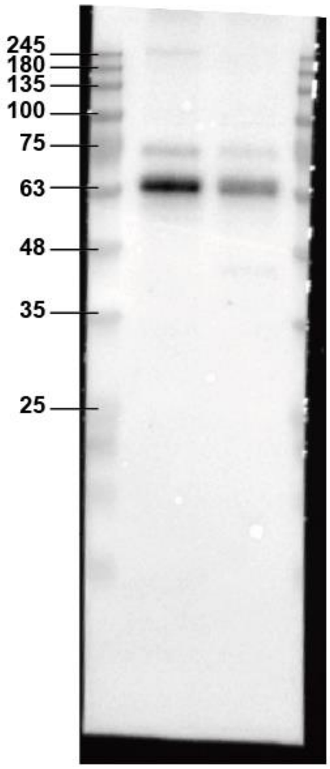


p-p44/42 MAPK(ERK1/2) (T202/Y204)
(marker merged)

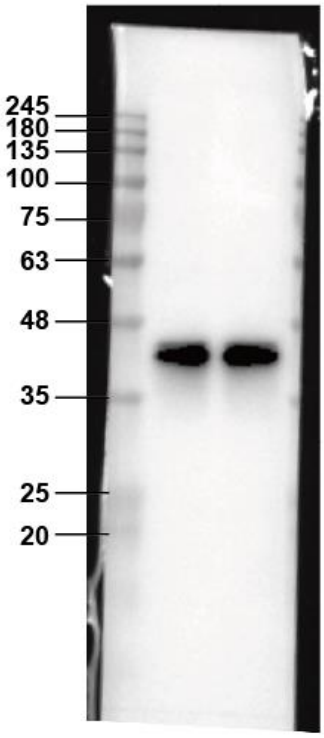

p44/42 MAPK (ERK1/2)
(marker merged)

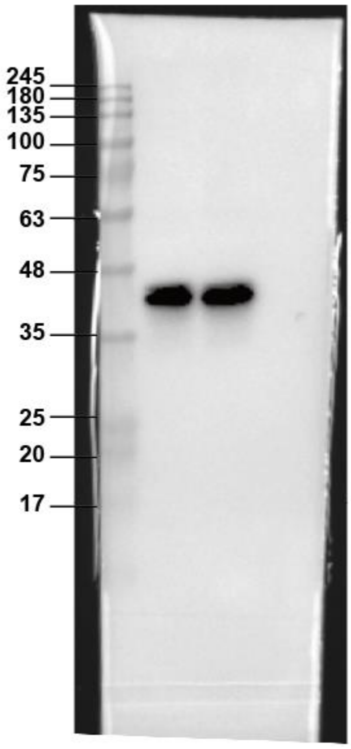


CT-myc
(marker merged)

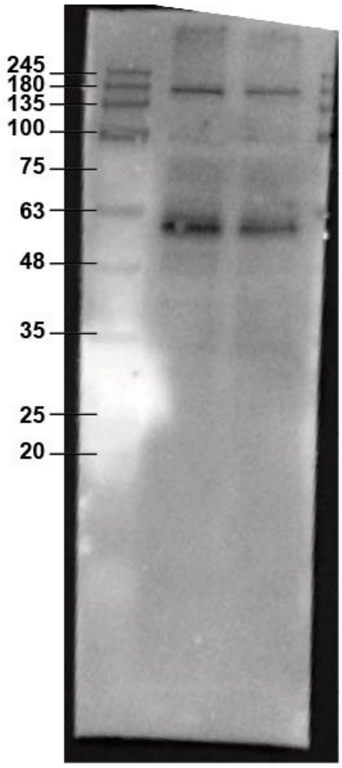


p53 and RRM1
(marker merged)

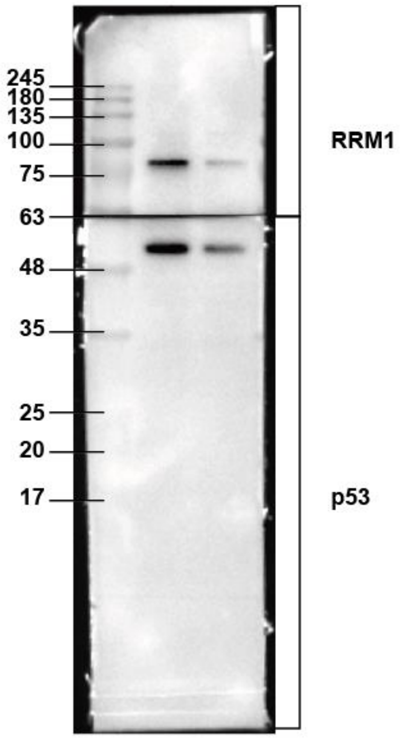


RRM2 (marker merged)

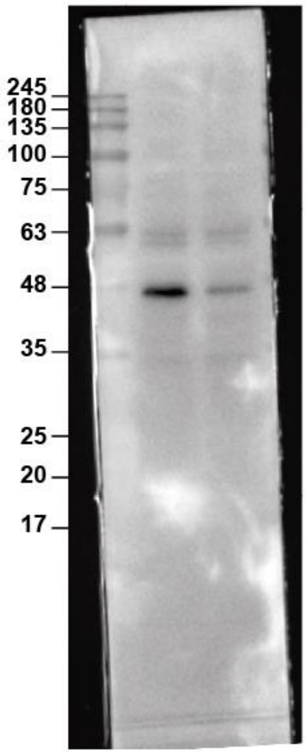


SAMHD1 (marker merged)

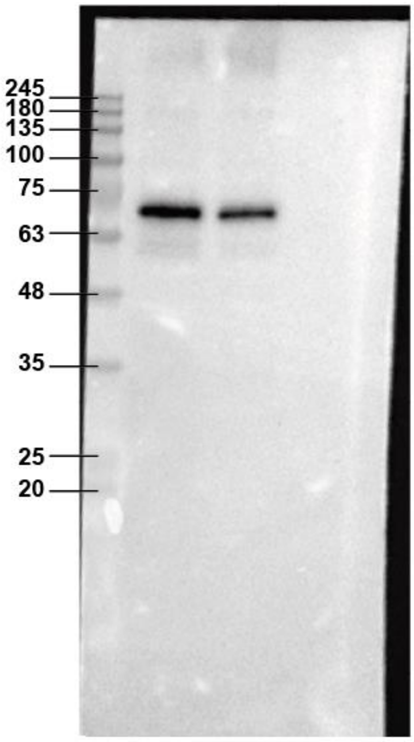


GAPDH (marker merged)

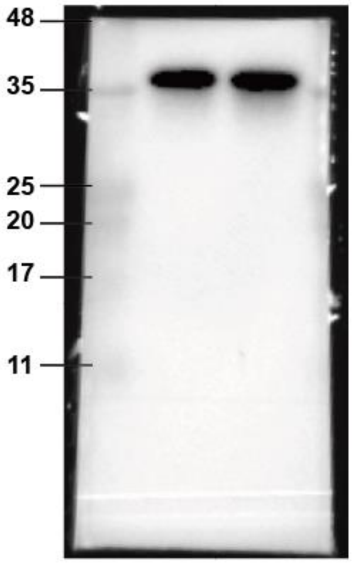

Fig. 4B.

| Time (minutes) | Control | | | | CT | | | |
|---|---|---|---|---|---|---|---|---|
| | 1 | 2 | 3 | 4 | 1 | 2 | 3 | 4 |
| 1.31 | 82.968107 | 85.647508 | 94.423427 | 92.085781 | 48.39327 | 49.599356 | 47.219131 | 43.810525 |
| 7.78 | 77.172536 | 75.831324 | 85.048869 | 83.9695 | 43.313629 | 45.199547 | 42.561634 | 39.519806 |
| 14.24 | 75.398182 | 74.145932 | 83.057656 | 81.604274 | 42.671889 | 43.742255 | 41.299599 | 39.358831 |
| 20.79 | 29.76843 | 26.504734 | 35.042469 | 32.677347 | 25.727005 | 25.832321 | 24.128409 | 22.357504 |
| 27.22 | 33.250664 | 29.547753 | 37.282952 | 36.734712 | 28.513797 | 28.805073 | 27.682436 | 24.360479 |
| 33.67 | 32.533588 | 29.450231 | 36.279682 | 35.257054 | 27.855918 | 27.614638 | 27.75421 | 24.21178 |
| 40.19 | 132.62077 | 136.27763 | 139.61621 | 153.36959 | 98.326148 | 96.262271 | 89.098638 | 89.652961 |
| 46.65 | 109.71598 | 114.46248 | 121.39811 | 123.48951 | 89.173259 | 88.16394 | 79.520674 | 79.627674 |
| 53.11 | 109.63871 | 110.09145 | 118.21601 | 120.38802 | 87.467463 | 87.960879 | 79.400947 | 79.815356 |
| 59.62 | 27.283512 | 20.746075 | 30.841103 | 30.701105 | 24.273137 | 24.060318 | 24.543622 | 23.012596 |
| 66.04 | 25.974175 | 19.547698 | 29.778808 | 28.501848 | 22.890413 | 21.662812 | 21.511211 | 20.943346 |
| 72.46 | 25.55626 | 20.473302 | 29.28715 | 28.316925 | 22.264421 | 21.302 | 21.534521 | 19.808546 |

Fig. 4C.

| Time (minutes) | Control | | | | CT | | | |
|---|---|---|---|---|---|---|---|---|
| | 1 | 2 | 3 | 4 | 1 | 2 | 3 | 4 |
| 1.31 | 32.015602 | 32.269969 | 32.378888 | 31.581529 | 34.356051 | 33.887974 | 34.120634 | 31.510465 |
| 7.79 | 31.863953 | 31.035269 | 32.959914 | 31.379112 | 33.94851 | 33.401549 | 33.472847 | 31.434188 |
| 14.27 | 31.679979 | 31.648982 | 32.280189 | 31.635872 | 33.621122 | 32.998579 | 33.389216 | 31.213694 |
| 20.84 | 52.495273 | 50.611687 | 53.197345 | 51.205292 | 44.105472 | 43.54565 | 42.599293 | 40.319837 |
| 27.32 | 53.969159 | 52.678833 | 55.286988 | 52.527832 | 44.583899 | 45.132363 | 43.904463 | 40.835764 |
| 33.79 | 55.945191 | 55.566503 | 58.008902 | 55.121068 | 44.954605 | 45.831073 | 44.691651 | 41.040612 |
| 40.35 | 19.159514 | 17.918184 | 20.146247 | 18.604005 | 21.817963 | 21.746867 | 22.338049 | 20.547343 |
| 46.82 | 15.636992 | 15.376977 | 15.930747 | 14.866451 | 15.145145 | 16.055383 | 16.138708 | 14.975219 |
| 53.27 | 14.696247 | 14.98707 | 14.994697 | 14.232899 | 13.859387 | 14.713111 | 14.668332 | 13.838398 |
| 59.72 | 14.108658 | 14.326699 | 14.183219 | 13.200069 | 13.086385 | 13.992757 | 14.003553 | 13.290913 |
| 66.20 | 13.242297 | 13.53479 | 13.174766 | 12.66103 | 12.694214 | 13.547346 | 13.372994 | 13.074257 |

**Fig. 5A.**

| dNTPs | Control | | | CdN | | | NT5M | | | CT | | |
|---|---|---|---|---|---|---|---|---|---|---|---|---|
| | 1 | 2 | 3 | 1 | 2 | 3 | 1 | 2 | 3 | 1 | 2 | 3 |
| dTTP | 10.3 | 12 | 8.96 | 9.9 | 11.2 | 10.59 | 6.7 | 10.1 | 9.88 | 5.6 | 5.5 | 6.91 |
| dCTP | 1.1 | 1.6 | 1.9 | 1.2 | 0.7 | 0.7 | 1.6 | 1.9 | 1.8 | 1.1 | 2.1 | 2.38 |
| dATP | 0 | 0.3 | 0.93 | 0 | 0.7 | 0.67 | 0 | 2.7 | 2.1 | 0 | 1.7 | 2.32 |
| dGTP | 2.5 | 1.8 | 2.39 | 2.1 | 1.9 | 2.01 | 1.5 | 1.7 | 2.33 | 1.2 | 1.4 | 1.6 |

**Fig. 5B.**

| | Control | | | | | | CdN | | | | | | NT5M | | | | | | CT | | | | | |
|---|---|---|---|---|---|---|---|---|---|---|---|---|---|---|---|---|---|---|---|---|---|---|---|---|
| | 1 | 2 | 3 | 4 | 5 | 6 | 1 | 2 | 3 | 4 | 5 | 6 | 1 | 2 | 3 | 4 | 5 | 6 | 1 | 2 | 3 | 4 | 5 | 6 |
| **Cell viability (% of control)** | 103 | 101 | 101 | 99 | 98 | 97 | 76 | 73 | 72 | 75 | 73 | 73 | 73 | 71 | 74 | 71 | 71 | 71 | 51 | 50 | 49 | 50 | 49 | 50 |

**Fig. 5C.**

*ECAR*

| Time (minutes) | Control | | | | CdN | | | | NT5M | | | | CT | | | |
|---|---|---|---|---|---|---|---|---|---|---|---|---|---|---|---|---|
| | 1 | 2 | 3 | 4 | 1 | 2 | 3 | 4 | 1 | 2 | 3 | 4 | 1 | 2 | 3 | 4 |
| 1.31 | 32.015602 | 32.269969 | 32.378888 | 31.581529 | 36.609701 | 37.425204 | 34.389234 | 35.25251 | 37.362211 | 33.062929 | 37.852313 | 31.042196 | 34.356051 | 33.887974 | 34.120634 | 31.510465 |
| 7.79 | 31.863953 | 31.035269 | 32.959914 | 31.379112 | 35.967408 | 36.45689 | 33.651601 | 35.46628 | 36.810748 | 31.984027 | 37.190456 | 30.315666 | 33.94851 | 33.401549 | 33.472847 | 31.434188 |
| 14.27 | 31.679979 | 31.648982 | 32.280189 | 31.635872 | 35.453392 | 36.415144 | 32.888028 | 35.180004 | 36.555339 | 32.066157 | 36.879157 | 29.842755 | 33.621122 | 32.998579 | 33.389216 | 31.213694 |
| 20.84 | 52.495273 | 50.611687 | 53.197345 | 51.205292 | 48.739647 | 51.272971 | 45.017555 | 47.131831 | 51.959271 | 42.271579 | 49.867919 | 38.151996 | 44.105472 | 43.54565 | 42.599293 | 40.319837 |
| 27.32 | 53.969159 | 52.678833 | 55.286988 | 52.527832 | 50.721312 | 54.046335 | 46.688706 | 48.408927 | 52.44569 | 43.485383 | 51.335939 | 38.979041 | 44.583899 | 45.132363 | 43.904463 | 40.835764 |
| 33.79 | 55.945191 | 55.566503 | 58.008902 | 55.121068 | 52.480321 | 55.772355 | 48.402402 | 48.990272 | 53.51412 | 44.392797 | 52.110105 | 39.334846 | 44.954605 | 45.831073 | 44.691651 | 41.040612 |
| 40.35 | 19.159514 | 17.918184 | 20.146247 | 18.604005 | 21.75099 | 21.853443 | 21.108007 | 21.597246 | 24.451226 | 21.760332 | 23.687701 | 19.073681 | 21.817963 | 21.746867 | 22.338049 | 20.547343 |
| 46.82 | 15.636992 | 15.376977 | 15.930747 | 14.866451 | 16.062087 | 17.196191 | 16.19354 | 15.756272 | 17.905561 | 15.662348 | 16.867034 | 14.135651 | 15.145145 | 16.055383 | 16.138708 | 14.975219 |
| 53.27 | 14.696247 | 14.98707 | 14.994697 | 14.232899 | 15.245546 | 15.961764 | 14.435282 | 14.328057 | 16.0187 | 13.998008 | 15.256222 | 12.621467 | 13.859387 | 14.713111 | 14.668332 | 13.838398 |
| 59.72 | 14.108658 | 14.326699 | 14.183219 | 13.200069 | 14.295947 | 14.99586 | 13.901899 | 13.049164 | 15.194073 | 13.165177 | 14.481589 | 12.116862 | 13.086385 | 13.992757 | 14.003553 | 13.290913 |
| 66.20 | 13.242297 | 13.53479 | 13.174766 | 12.66103 | 13.696489 | 14.293146 | 13.370908 | 12.503924 | 14.294792 | 12.715222 | 13.575681 | 11.639543 | 12.694214 | 13.547346 | 13.372994 | 13.074257 |

*OCR*

| Time (minutes) | Control | | | | CdN | | | | NT5M | | | | CT | | | |
|---|---|---|---|---|---|---|---|---|---|---|---|---|---|---|---|---|
| | **1** | **2** | **3** | **4** | **1** | **2** | **3** | **4** | **1** | **2** | **3** | **4** | **1** | **2** | **3** | **4** |
| **1.31** | 82.968107 | 85.647508 | 94.423427 | 92.085781 | 82.38871 | 84.568053 | 77.496125 | 86.447258 | 73.034152 | 70.260549 | 69.20607 | 66.018804 | 48.39327 | 49.599356 | 47.219131 | 43.810525 |
| **7.78** | 77.172536 | 75.831324 | 85.048869 | 83.9695 | 75.076184 | 78.055025 | 70.883904 | 80.129356 | 65.27999 | 63.035173 | 62.62228 | 60.15115 | 43.313629 | 45.199547 | 42.561634 | 39.519806 |
| **14.24** | 75.398182 | 74.145932 | 83.057656 | 81.604274 | 72.859406 | 76.575527 | 67.732981 | 78.719378 | 63.256286 | 61.727639 | 61.61503 | 59.815132 | 42.671889 | 43.742255 | 41.299599 | 39.358831 |
| **20.79** | 29.76843 | 26.504734 | 35.042469 | 32.677347 | 31.984966 | 34.527268 | 32.876536 | 35.48724 | 32.506292 | 30.142665 | 30.439085 | 29.572643 | 25.727005 | 25.832321 | 24.128409 | 22.357504 |
| **27.22** | 33.250664 | 29.547753 | 37.282952 | 36.734712 | 33.754944 | 36.376246 | 34.005905 | 37.222044 | 34.825808 | 33.034752 | 33.253253 | 31.533485 | 28.513797 | 28.805073 | 27.682436 | 24.360479 |
| **33.67** | 32.533588 | 29.450231 | 36.279682 | 35.257054 | 33.557245 | 35.543296 | 33.676272 | 36.251925 | 33.914682 | 33.28276 | 32.580689 | 31.416477 | 27.855918 | 27.614638 | 27.75421 | 24.21178 |
| **40.19** | 132.62077 | 136.27763 | 139.61621 | 153.36959 | 146.70534 | 150.49423 | 112.86663 | 147.313 | 122.48686 | 134.28078 | 124.57388 | 116.62688 | 98.326148 | 96.262271 | 89.098638 | 89.652961 |
| **46.65** | 109.71598 | 114.46248 | 121.39811 | 123.48951 | 124.92418 | 128.52501 | 100.12212 | 130.36601 | 108.14534 | 114.50694 | 107.56864 | 104.98008 | 89.173259 | 88.16394 | 79.520674 | 79.627674 |
| **53.11** | 109.63871 | 110.09145 | 118.21601 | 120.38802 | 121.75399 | 125.55833 | 100.54625 | 126.24782 | 107.5549 | 110.76276 | 106.57719 | 105.20959 | 87.467463 | 87.960879 | 79.400947 | 79.815356 |
| **59.62** | 27.283512 | 20.746075 | 30.841103 | 30.701105 | 27.455005 | 29.531514 | 27.378238 | 31.561237 | 30.936806 | 27.069097 | 26.835337 | 26.968008 | 24.273137 | 24.060318 | 24.543622 | 23.012596 |
| **66.04** | 25.974175 | 19.547698 | 29.778808 | 28.501848 | 26.79992 | 28.903468 | 27.104247 | 29.141732 | 28.794869 | 27.056121 | 25.940132 | 24.773595 | 22.890413 | 21.662812 | 21.511211 | 20.943346 |
| **72.46** | 25.55626 | 20.473302 | 29.28715 | 28.316925 | 26.039288 | 28.906183 | 26.687285 | 29.822857 | 27.598599 | 25.391516 | 25.957021 | 24.921989 | 22.264421 | 21.302 | 21.534521 | 19.808546 |

**Fig. 5D.**

HK1, GLUD and TK (marker merged)

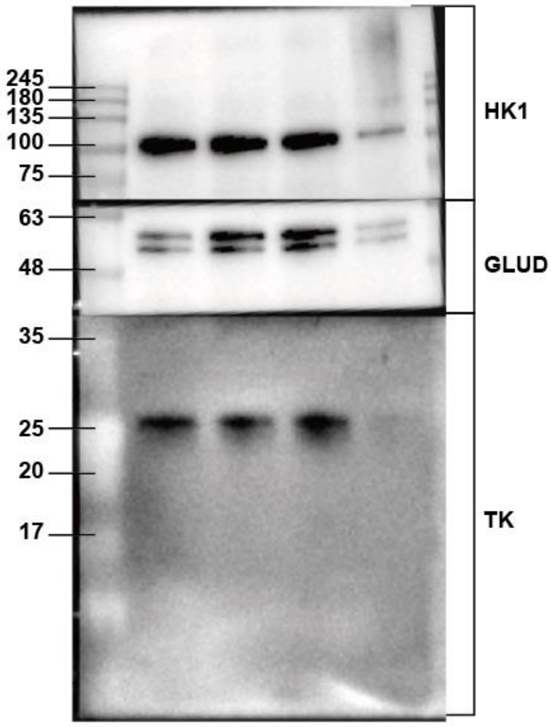


HK2 and GLS1 (marker merged)

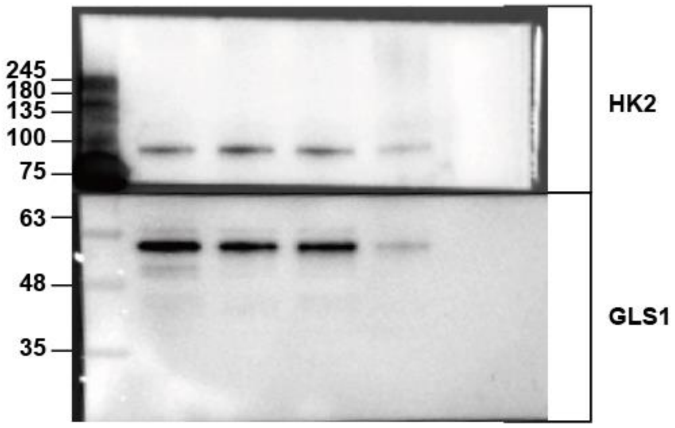


OGDH (marker merged)

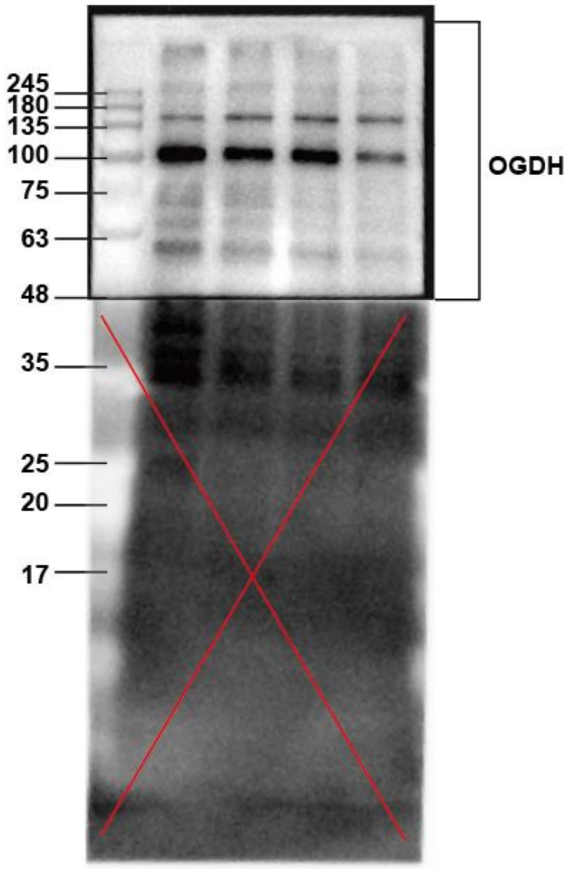


P53 and GAPDH (marker merged)

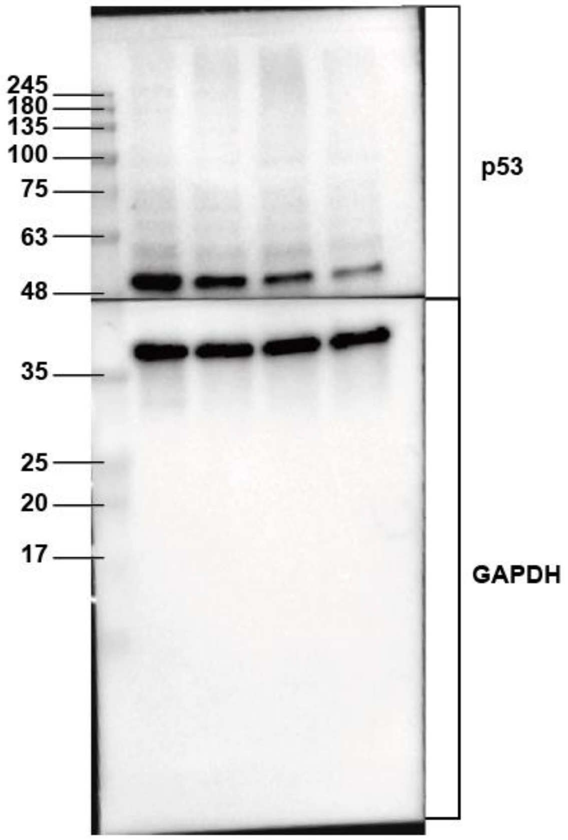

PARP and $\gamma$ H2AX
(marker merged)

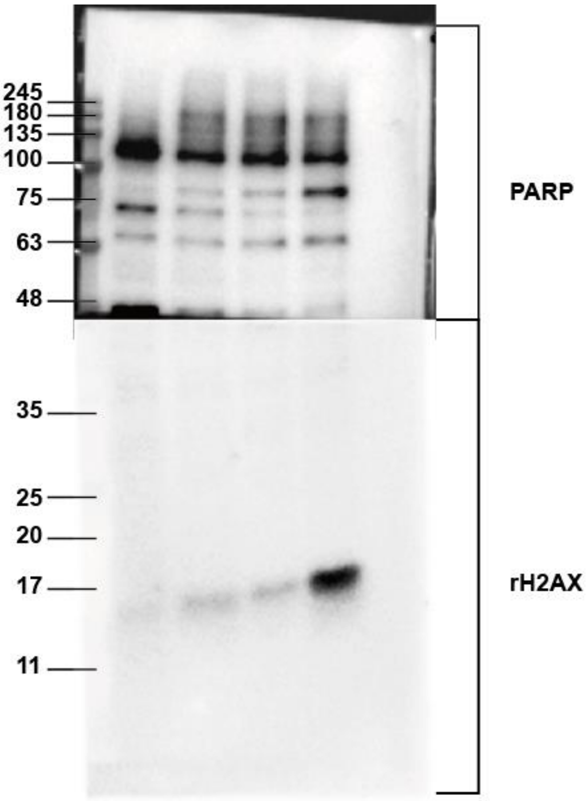


TYMS
(marker merged)

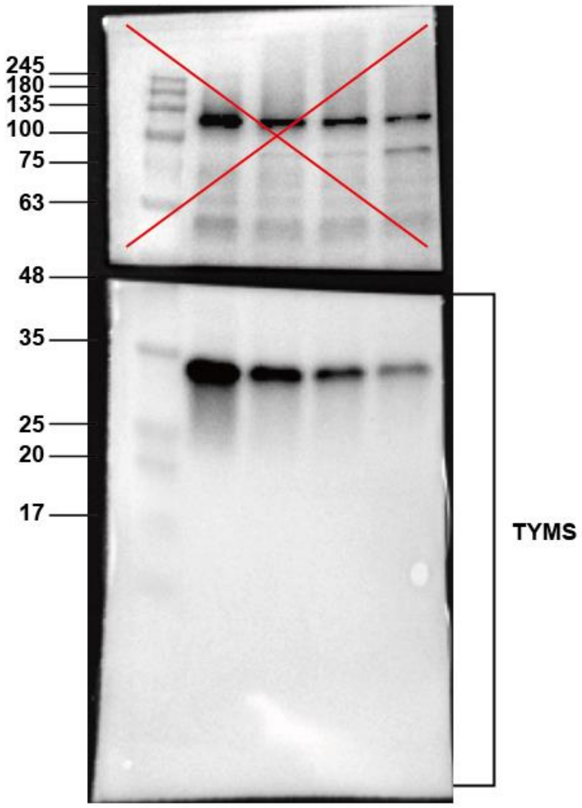

**Fig. 6.**

| | cdN | | | NT5M | | | CT | | |
|---|---|---|---|---|---|---|---|---|---|
| | MMR | | | MMR | | | MMR | | |
| | 6h | 12h | 24h | 6h | 12h | 24h | 6h | 12h | 24h |
| EXO1 | 0.0565587 | -0.3124959 | -0.4086755 | -0.1160575 | -0.2946687 | -0.3354965 | -0.0695171 | -1.5734521 | -1.3071933 |
| RFC4 | -0.1645086 | -0.3691847 | -0.3193147 | -0.0759887 | -0.2477853 | -0.283848 | 0.1966553 | -0.7293107 | -0.4643601 |
| RFC2 | -0.5775084 | -0.9420429 | -0.814871 | -0.5233714 | -1.0516782 | -0.5900311 | -0.6476196 | -1.4596191 | -1.3099906 |
| MSH6 | -0.2758317 | -0.8029334 | -0.7009057 | -0.3336657 | -0.7260194 | -0.5072236 | -0.5428553 | -1.5720896 | -1.4827104 |
| MSH2 | -0.1884093 | -0.3267295 | -0.3997631 | -0.2249786 | -0.4030728 | -0.2631714 | -0.2400782 | -0.89413 | -1.018057 |
| SSBP1 | -0.1417658 | -0.1039877 | 0.0833396 | -0.2129278 | -0.2741773 | 0.126558 | -0.2764531 | -0.2018355 | 0.0440568 |
| POLD2 | -0.3764437 | -0.2544767 | -0.472477 | -0.371695 | -0.3368741 | -0.5919978 | -0.7121185 | -1.0837071 | -0.9691003 |
| PMS2 | 0.0855369 | -0.2523155 | -0.4768134 | 0.0847207 | -0.2003127 | -0.1812982 | 0.0718041 | -0.360222 | -0.3524901 |
| PMS1 | -0.2684799 | -0.4843296 | 0.0753894 | -0.3029162 | -0.43343 | 0.1860662 | 0.0367031 | 0.0442207 | 0.1684725 |
| LIG4 | 0.5937204 | 0.6436042 | 0.0717674 | 0.905805 | 0.7689196 | 0.3735408 | 0.8871198 | 0.9792666 | 0.8613025 |
| RFC3 | -0.0833424 | -0.2776466 | -0.560133 | -0.2147279 | -0.374868 | -0.4085908 | -0.2236062 | -1.4531499 | -1.1894934 |
| RPA2 | -0.7872111 | -1.005362 | -0.2474367 | -0.8719946 | -1.1959931 | -0.4066084 | -1.4448529 | -1.680028 | -1.3869532 |
| RPA1 | -0.516178 | -0.5804123 | -0.0794818 | -0.3007946 | -0.5358112 | -0.0039872 | -0.3551941 | -0.5631453 | -0.3211409 |

| | BER | | | BER | | | BER | | |
|---|---|---|---|---|---|---|---|---|---|
| | 6h | 12h | 24h | 6h | 12h | 24h | 6h | 12h | 24h |
| PARP1 | -0.5858139 | -0.8828567 | -0.5576994 | -0.794912 | -1.0390045 | -0.5306257 | -1.2198699 | -1.9500973 | -1.5833055 |
| MBD4 | 0.2969304 | 0.4292299 | 0.3368163 | 0.412055 | 0.5245247 | 0.2236288 | 0.4444204 | 0.6766581 | 0.63865 |
| POLD1 | -0.4435102 | -0.6594875 | -0.379661 | -0.5098954 | -0.6979867 | -0.5228876 | -0.6778135 | -1.4963881 | -1.2623773 |
| LIG1 | -0.2491348 | -0.5606783 | -0.75032 | -0.1283513 | -0.5329703 | -0.7138522 | -0.1600784 | -1.6501054 | -1.3497417 |
| PCNA | -0.8429777 | -1.1126298 | -0.668581 | -0.9034698 | -1.1562104 | -0.6262422 | -1.4078498 | -1.6982074 | -1.5680365 |
| NEIL3 | -0.5459949 | -1.2383644 | -1.0947872 | -0.5323001 | -1.1943702 | -1.244453 | -0.5795403 | -2.2072544 | -1.9125413 |
| UNG | -0.2596428 | -0.6770566 | -0.4737257 | -0.3479369 | -0.8004243 | -0.4251103 | -0.4400337 | -1.7634223 | -1.6997404 |
| TDG | 0.8790681 | 0.9663347 | 0.4595558 | 0.9306975 | 0.9616962 | 0.5374281 | 1.0994944 | 0.8575203 | 0.8104265 |
| POLD3 | -0.1967389 | -0.7082563 | -0.4189876 | -0.2224015 | -0.5721496 | -0.2259848 | -0.1641495 | -1.1969976 | -0.9693644 |
| MUTYH | -0.3503151 | -0.3297953 | -0.1012924 | -0.0989029 | -0.2140614 | -0.2961657 | 0.0553742 | 0.2320575 | 0.3558478 |

| | | DNA repair alteration | | | DNA repair alteration | | | DNA repair alteration | |
|---|---|---|---|---|---|---|---|---|---|
| | 6h | 12h | 24h | 6h | 12h | 24h | 6h | 12h | 24h |
| MDM4 | 0.3377931 | 0.0165065 | -0.0671922 | 0.4903466 | 0.0791262 | 0.0416218 | 0.4376327 | 0.246338 | 0.1889392 |
| UNG | -0.2596428 | -0.6770566 | -0.4737257 | -0.3479369 | -0.8004243 | -0.4251103 | -0.4400337 | -1.7634223 | -1.6997404 |
| TDG | 0.8790681 | 0.9663347 | 0.4595558 | 0.9306975 | 0.9616962 | 0.5374281 | 1.0994944 | 0.8575203 | 0.8104265 |
| POLB | 0.0392365 | 0.1121792 | 0.3359962 | 0.1087094 | 0.282637 | 0.3129992 | 0.2666823 | 0.5396051 | 0.7255315 |
| NEIL3 | -0.5459949 | -1.2383644 | -1.0947872 | -0.5323001 | -1.1943702 | -1.244453 | -0.5795403 | -2.2072544 | -1.9125413 |
| LIG1 | -0.2491348 | -0.5606783 | -0.75032 | -0.1283513 | -0.5329703 | -0.7138522 | -0.1600784 | -1.6501054 | -1.3497417 |
| PARP1 | -0.5858139 | -0.8828567 | -0.5576994 | -0.794912 | -1.0390045 | -0.5306257 | -1.2198699 | -1.9500973 | -1.5833055 |
| APEX1 | -0.2594535 | -0.5217658 | -0.3069676 | -0.2678822 | -0.4300773 | -0.2480809 | -0.4086679 | -0.6358078 | -0.5499542 |
| MUTYH | -0.3503151 | -0.3297953 | -0.1012924 | -0.0989029 | -0.2140614 | -0.2961657 | 0.0553742 | 0.2320575 | 0.3558478 |
| MSH2 | -0.2758317 | -0.8029334 | -0.7009057 | -0.3336657 | -0.7260194 | -0.5072236 | -0.5428553 | -1.5720896 | -1.4827104 |
| MSH3 | 0.3475578 | -0.1266665 | -0.5658979 | 0.2738874 | -0.1275694 | -0.2955533 | 0.3494797 | -0.4844422 | -0.4622107 |
| MSH6 | -0.1884093 | -0.3267295 | -0.3997631 | -0.2249786 | -0.4030728 | -0.2631714 | -0.2400782 | -0.89413 | -1.018057 |
| MLH3 | 0.1072063 | -0.3414035 | -0.379861 | 0.1636273 | -0.20269 | -0.2637236 | 0.2908743 | 0.0214864 | -0.3402549 |
| PMS2 | -0.2684799 | -0.4843296 | 0.0753894 | -0.3029162 | -0.43343 | 0.1860662 | 0.0367031 | 0.0442207 | 0.1684725 |

| | | M | | | M | | | M | |
|---|---|---|---|---|---|---|---|---|---|
| Data Table | 6h | 12h | 24h | 6h | 12h | 24h | 6h | 12h | 24h |
| PLK1 | -0.951018 | -1.0766702 | -0.8272736 | -0.9523511 | -1.0914563 | -1.0008432 | -1.051997 | -1.377158 | -1.7626469 |
| AURKA | -0.6120789 | -0.7504562 | -0.5589107 | -0.4993031 | -0.812578 | -0.4685916 | -0.5401241 | -0.7495531 | -1.0745708 |
| CENPE | 0.0205288 | -0.2464473 | -0.739652 | -0.0249224 | -0.0707229 | -0.5837893 | -0.1888 | -0.2168778 | -0.6994169 |
| TPX2 | 0.0222248 | 0.0692874 | -0.2729413 | 0.0326867 | 0.0840373 | -0.2158478 | 0.0328861 | 0.2304974 | -0.2271108 |
| CKS2 | -0.2391188 | -0.5553357 | 0.048158 | -0.1107753 | -0.588413 | -0.029811 | -0.1540923 | -0.5759859 | -0.7166518 |
| BUB1 | -0.1263918 | -0.4315993 | -0.5587956 | -0.1263642 | -0.379438 | -0.5240301 | -0.1353418 | -0.4247906 | -0.9637669 |
| CENPF | -0.4402944 | -0.751502 | -0.8109069 | -0.6909029 | -0.8998274 | -0.8769158 | -1.0577683 | -1.3179629 | -1.7726307 |
| NUF2 | -0.4134109 | -1.0399278 | -1.417813 | -0.4496281 | -0.9973887 | -1.1882234 | -0.6696465 | -1.3667131 | -1.8551152 |
| CDC20 | -0.722702 | -0.883236 | -0.4829245 | -0.5510621 | -0.9310782 | -0.601708 | -0.8549418 | -1.1660206 | -0.9421821 |
| TACC3 | -0.4882392 | -0.6835346 | -0.5928058 | -0.4885641 | -0.6954907 | -0.6059738 | -0.4613606 | -0.8393867 | -1.1622156 |
| BUB1B | -0.6622769 | -0.9823395 | -0.7970592 | -0.6734759 | -1.1687587 | -0.6891146 | -0.9395839 | -1.7418371 | -1.6487062 |
| UBE2S | -0.2648646 | -0.2686623 | 0.0563853 | -0.3387961 | -0.2100532 | -0.0723449 | -0.539888 | -0.5263656 | -0.4932681 |

| | | RRG | | | RRG | | | RRG | |
|---|---|---|---|---|---|---|---|---|---|
| Data Table | 6h | 12h | 24h | 6h | 12h | 24h | 6h | 12h | 24h |
| BUB1 | -0.1263918 | -0.4315993 | -0.5587956 | -0.1263642 | -0.379438 | -0.5240301 | -0.1353418 | -0.4247906 | -0.9637669 |
| BUB3 | -0.0551474 | 0.0442831 | 0.0986689 | -0.071053 | -0.1409551 | 0.1754501 | -0.3503179 | -0.5935964 | -0.18685 |
| CDC25B | -0.2833711 | -0.1219289 | 0.3821393 | -0.477117 | -0.2817467 | 0.5597651 | -0.8540547 | -1.1803315 | -0.7697322 |
| CDC25A | -0.5434486 | -0.8291908 | -0.3143245 | -0.6567169 | -0.7675021 | -0.3472408 | -0.58904 | -0.9468317 | -0.8193621 |
| BIRC5 | -0.7195081 | -1.1635815 | -0.9965483 | -0.77521 | -1.207933 | -1.0463975 | -0.9557437 | -1.5712252 | -1.8234025 |
| CCNE2 | -0.3238512 | -0.3283944 | -0.7695402 | -0.2853756 | -0.8598421 | -0.7497422 | -0.8556134 | -1.5717535 | -1.7966276 |
| CCNB1 | -0.5314298 | -0.9310792 | -0.6320076 | -0.437926 | -0.901904 | -0.7157633 | -0.4346602 | -0.9140817 | -1.2468393 |
| CCNB2 | -0.3629944 | -0.886138 | -0.924768 | -0.2954405 | -0.7802526 | -1.0072825 | -0.3924214 | -0.7676881 | -1.3484163 |
| CCNF | -0.9457399 | -0.9527949 | -0.6609309 | -1.0810671 | -1.0286588 | -0.8307716 | -1.4029473 | -1.5622433 | -1.3733553 |
| CDC20 | -0.722702 | -0.883236 | -0.4829245 | -0.5510621 | -0.9310782 | -0.601708 | -0.8549418 | -1.1660206 | -0.9421821 |
| CDC6 | 1.175002 | 1.0538018 | 0.2368769 | 1.2759198 | 1.1846511 | 0.2457415 | 2.2332356 | 2.3566867 | 0.765094 |
| CDCA3 | -0.7369761 | -1.384824 | -1.101294 | -0.9109816 | -1.3336882 | -1.0809714 | -1.1122873 | -1.8138584 | -2.0465047 |
| CENPF | -0.4402944 | -0.751502 | -0.8109069 | -0.6909029 | -0.8998274 | -0.8769158 | -1.0577683 | -1.3179629 | -1.7726307 |
| CKS1B | -0.682307 | -0.7679148 | -0.0978478 | -0.7804344 | -1.0155613 | -0.2383637 | -1.0654487 | -1.3701011 | -0.8153289 |
| DTL | 0.3061132 | 0.1640356 | 0.029194 | 0.3330395 | 0.1357512 | 0.2753659 | 0.3894689 | -0.211438 | -0.9474233 |
| MCM2 | -0.3593145 | -0.5940672 | -0.471442 | -0.4789116 | -0.5897413 | -0.51044 | -0.7691982 | -1.2560766 | -1.3477832 |
| MCM6 | -0.2014314 | -0.3949882 | -0.9785165 | -0.2929906 | -0.5689751 | -0.9654439 | -0.4383697 | -0.7261204 | -1.4361855 |
| PLK1 | -0.951018 | -1.0766702 | -0.8272736 | -0.9523511 | -1.0914563 | -1.0008432 | -1.051997 | -1.377158 | -1.7626469 |

| | | S | | | S | | | S | |
|---|---|---|---|---|---|---|---|---|---|
| Data Table | 6h | 12h | 24h | 6h | 12h | 24h | 6h | 12h | 24h |
| CDC6 | 1.175002 | 1.0538018 | 0.2368769 | 1.2759198 | 1.1846511 | 0.2457415 | 2.2332356 | 2.3566867 | 0.765094 |
| PCNA | -0.8429777 | -1.1126298 | -0.668581 | -0.9034698 | -1.1562104 | -0.6262422 | -1.4078498 | -1.6565566 | -1.5680365 |
| RRM2 | -0.1935703 | -0.4637175 | -0.6338881 | -0.3619932 | -0.6467523 | -0.6027653 | -0.6475409 | -1.4179881 | -2.0012838 |
| E2F8 | -0.2761477 | -1.2638161 | -1.3973241 | -0.0880725 | -0.948209 | -1.3817853 | 0.3531462 | -0.8043411 | -2.1695552 |
| MCM5 | -0.3370953 | -0.587032 | -0.5867879 | -0.338043 | -0.5159012 | -0.7338556 | -0.5166757 | -0.686145 | -1.4060215 |
| ATAD2 | 0.1717655 | 0.0707771 | -0.5510476 | 0.1412388 | 0.0414055 | -0.347259 | 0.1754684 | -0.0893408 | -1.2253005 |
| CDCA5 | -0.320287 | -0.1831593 | 0.137044 | -0.3679604 | -0.3370346 | 0.1912147 | -0.4689051 | -0.9148103 | -0.9680416 |
| FEN1 | -0.5416892 | -0.6473563 | -0.2155874 | -0.5398102 | -0.7743834 | -0.1802445 | -0.9566514 | -1.3519596 | -1.025199 |

| | | | | | | | | | |
|---|---|---|---|---|---|---|---|---|---|
| POLD1 | -0.4435102 | -0.6594875 | -0.379661 | -0.5098954 | -0.6979867 | -0.5228876 | -0.6778135 | -1.0870087 | -1.2623773 |
| POLD3 | -0.1967389 | -0.7082563 | -0.4189876 | -0.2224015 | -0.5721496 | -0.2259848 | -0.1641495 | -0.9799648 | -0.9693644 |
| RFC4 | -0.5775084 | -0.9420429 | -0.814871 | -0.5233714 | -1.0516782 | -0.5900311 | -0.6476196 | -1.4479196 | -1.3099906 |
| E2F1 | -0.4872361 | -0.9894162 | -0.8364148 | -0.6127526 | -0.8603588 | -1.021701 | -0.6248251 | -1.0703002 | -1.8050203 |

**Fig. 7A.**

| | dNTPs | | |
|---|---|---|---|
| | 1 | 2 | 3 |
| MDA-MB-231 | 17.01 | 18.85 | 13.52 |
| BT-549 | 22.03 | 20.89 | 27.89 |
| MDA-MB-468 | 56.74 | 58.66 | 59.78 |

**Fig. 7B.**

| | Basal ECAR | | | |
|---|---|---|---|---|
| | 1 | 2 | 3 | 4 |
| MDA-MB-231 | 32.015602 | 32.269969 | 32.378888 | 31.581529 |
| BT-549 | 77.359686 | 84.872227 | 81.888099 | 81.640969 |
| MDA-MB-468 | 66.116488 | 65.244702 | 58.786557 | 58.045178 |

| | Basal OCR | | | |
|---|---|---|---|---|
| | 1 | 2 | 3 | 4 |
| MDA-MB-231 | 82.968107 | 85.647508 | 94.423427 | 92.085781 |
| BT-549 | 188.554286 | 192.36109 | 192.09838 | 194.43096 |
| MDA-MB-468 | 279.957637 | 251.43249 | 254.97411 | 248.81868 |

**Fig. 7C.**

| | Maximal ECAR | | | |
|---|---|---|---|---|
| | 1 | 2 | 3 | 4 |
| MDA-MB-231 | 52.495273 | 50.611687 | 53.197345 | 51.205292 |
| BT-549 | 92.708864 | 103.40958 | 97.92809 | 101.90857 |
| MDA-MB-468 | 96.383168 | 98.296111 | 90.04696 | 85.786411 |

| | Maximal OCR | | | |
|---|---|---|---|---|
| | 1 | 2 | 3 | 4 |
| MDA-MB-231 | 132.620765 | 136.27763 | 139.61621 | 153.36959 |
| BT-549 | 440.966465 | 426.59953 | 436.70573 | 436.33102 |
| MDA-MB-468 | 465.327592 | 441.48003 | 452.04562 | 445.40519 |

**Fig. 7D.**

| | Glycolytic reserve | | | |
|---|---|---|---|---|
| | 1 | 2 | 3 | 4 |
| MDA-MB-231 | 20.47967 | 18.34172 | 20.81846 | 19.62376 |
| BT-549 | 15.34918 | 18.53735 | 16.03999 | 20.2676 |
| MDA-MB-468 | 30.26668 | 33.05141 | 31.2604 | 27.74123 |

| | Spare respiratory capacity rates | | | |
|---|---|---|---|---|
| | 1 | 2 | 3 | 4 |
| MDA-MB-231 | 49.65266 | 50.63013 | 45.19278 | 61.28381 |
| BT-549 | 252.4122 | 234.2384 | 244.6074 | 241.9001 |
| MDA-MB-468 | 185.37 | 190.0475 | 197.0715 | 196.5865 |

**Fig. 7E.**

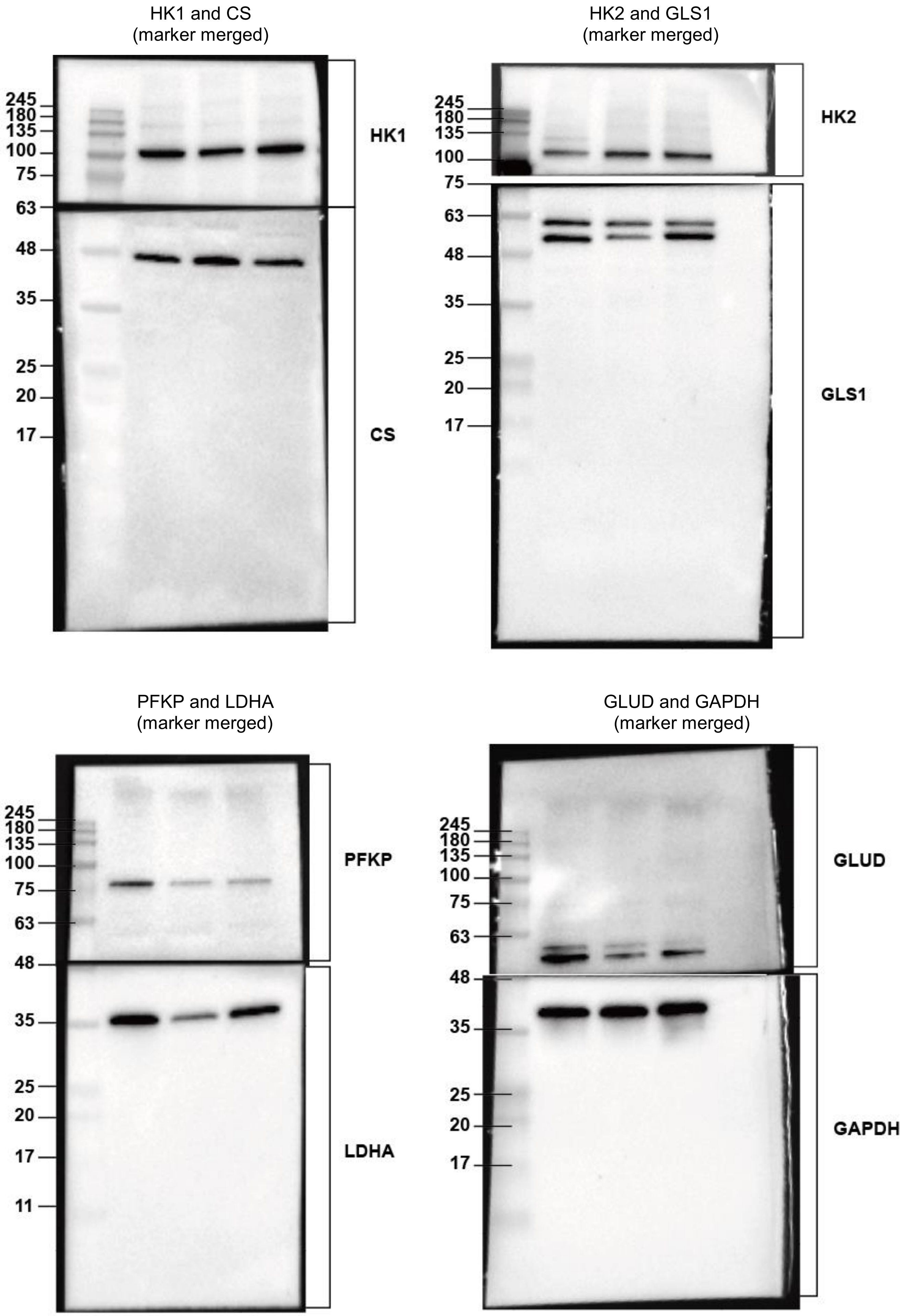
HK1 and CS
(marker merged)
245
180
135
100
75
63
48
35
25
20
17
HK1
CS
HK2 and GLS1
(marker merged)
245
180
135
100
75
63
48
35
25
20
17
HK2
GLS1
PFKP and LDHA
(marker merged)
245
180
135
100
75
63
48
35
25
20
17
11
PFKP
LDHA
GLUD and GAPDH
(marker merged)
245
180
135
100
75
63
48
35
25
20
17
GLUD
GAPDH

SDHA
(marker merged)

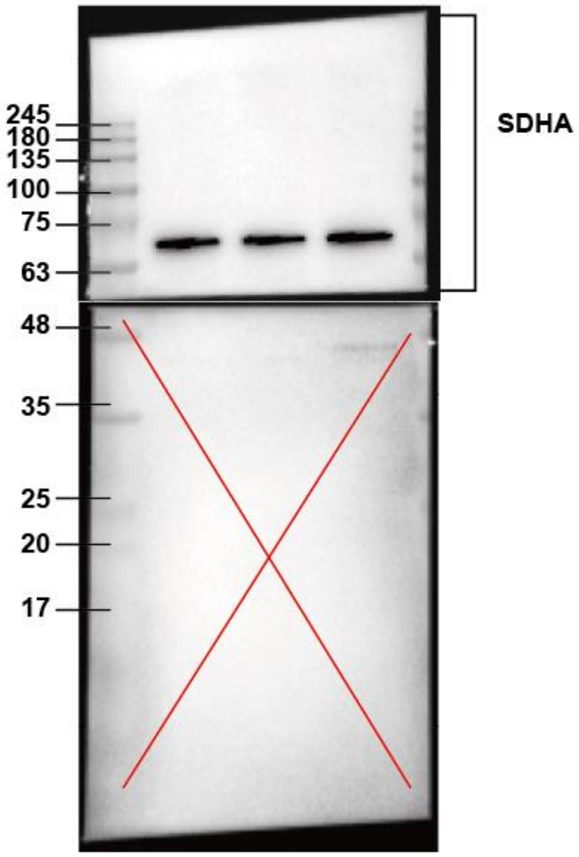


OGDH and FH
(marker merged)

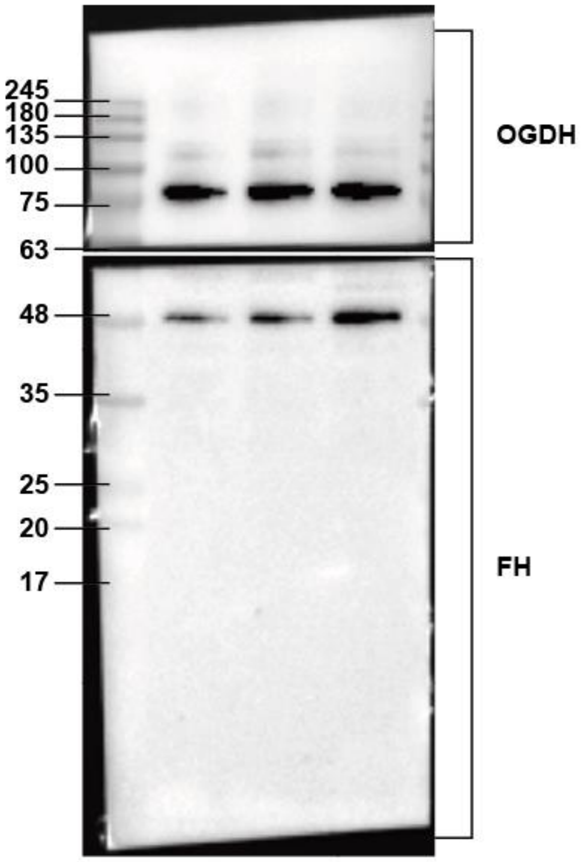


ACO2 and IDH2 (marker merged)

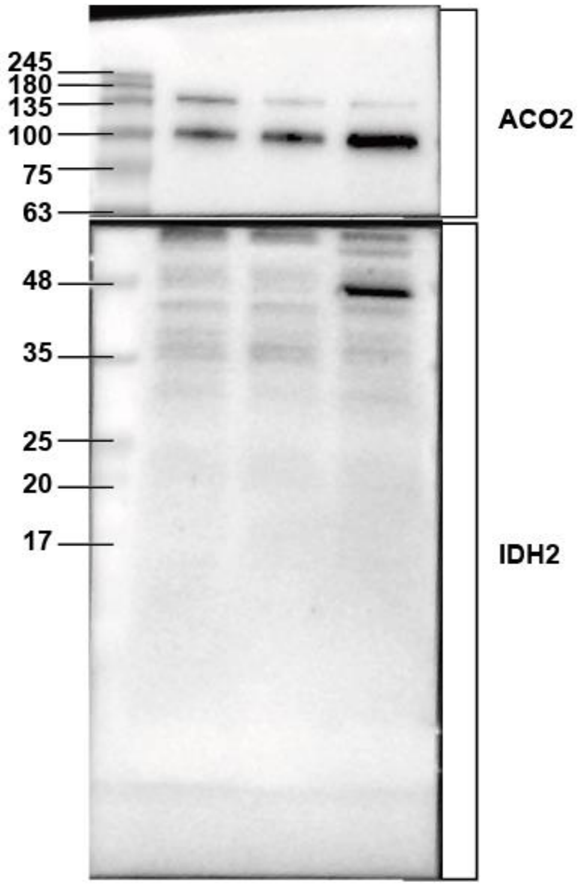


PKM1/2 and PDH (marker merged)

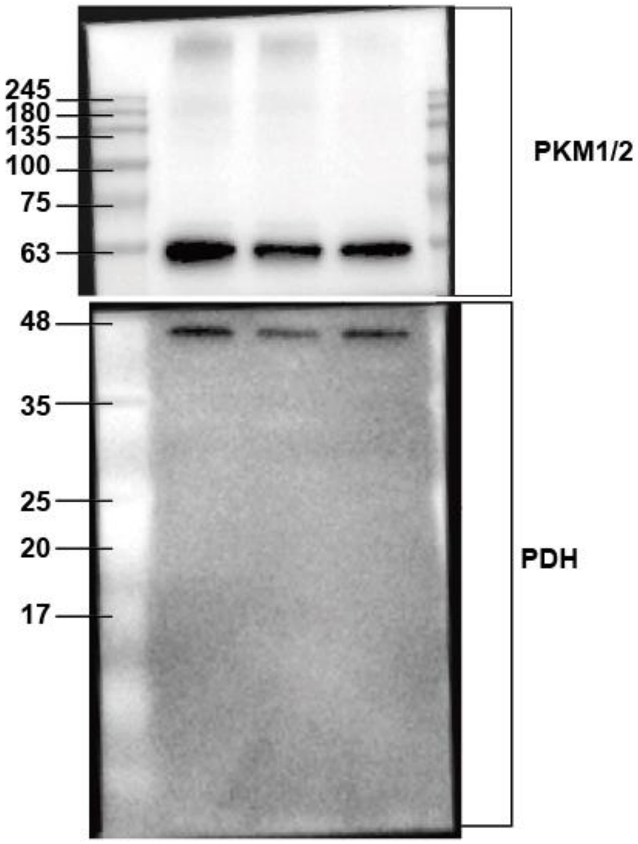

Fig. 8A.

| | Control | | | CT | | | Control | | | CT | | | Control | | | CT | | |
|---|---|---|---|---|---|---|---|---|---|---|---|---|---|---|---|---|---|---|
| | **1** | **2** | **3** | **1** | **2** | **3** | **1** | **2** | **3** | **1** | **2** | **3** | **1** | **2** | **3** | **1** | **2** | **3** |
| **dTTP** | 12.98 | 14.1 | 13.8 | 5.47 | 5.45 | 5.34 | 14.29 | 14.35 | 14.33 | 9.33 | 10.4 | 9.45 | 26.28 | 27.99 | 26.88 | 23.26 | 23.82 | 22.99 |
| **dCTP** | 2.3 | 2.68 | 2.45 | 2.68 | 2 | 2.35 | 4.21 | 3.9 | 4 | 3.86 | 3.33 | 3.55 | 12.55 | 14.62 | 13.56 | 12.54 | 13.55 | 13.14 |
| **dATP** | 0.5 | 0.58 | 0.63 | 0.5 | 0 | 0.4 | 2.19 | 0.98 | 1.88 | 1.37 | 0.64 | 1.21 | 10.97 | 9.23 | 9.88 | 9.23 | 7.33 | 8.99 |
| **dGTP** | 1.23 | 1.49 | 1.55 | 0 | 0 | 0 | 1.34 | 1.66 | 1.42 | 0.97 | 1.09 | 1.11 | 6.94 | 6.82 | 6.45 | 4.31 | 3.93 | 4.21 |

Fig. 8B.

| **Basal ECAR** | **Control** | | | | **CT** | | | |
|---|---|---|---|---|---|---|---|---|
| | **1** | **2** | **3** | **4** | **1** | **2** | **3** | **4** |
| **MDA-MB-231** | 32.015602 | 32.269969 | 32.378888 | 31.581529 | 34.356051 | 33.887974 | 34.120634 | 31.510465 |
| **BT-549** | 77.359686 | 84.872227 | 81.888099 | 81.640969 | 68.459454 | 71.57466 | 67.352519 | 65.28791 |
| **MDA-MB-468** | 66.116488 | 65.244702 | 58.786557 | 58.045178 | 30.731891 | 35.194234 | 34.563946 | 31.698793 |

| **Basal OCR** | **Control** | | | | **CT** | | | |
|---|---|---|---|---|---|---|---|---|
| | **1** | **2** | **3** | **4** | **1** | **2** | **3** | **4** |
| **MDA-MB-231** | 82.968107 | 85.647508 | 94.423427 | 92.085781 | 48.39327 | 49.599356 | 47.219131 | 43.810525 |
| **BT-549** | 188.554286 | 192.36109 | 192.09838 | 194.43096 | 126.7127 | 132.96802 | 121.75831 | 123.61615 |
| **MDA-MB-468** | 279.957637 | 251.43249 | 254.97411 | 248.81868 | 166.82875 | 154.55749 | 165.99434 | 144.48739 |

**Fig. 8C.**

| | | Control | | | | CT | | | |
|---|---|---|---|---|---|---|---|---|---|
| | | 1 | 2 | 3 | 4 | 1 | 2 | 3 | 4 |
| Basal ECAR | MDA-MB-231 | 32.015602 | 32.269969 | 32.378888 | 31.581529 | 34.356051 | 33.887974 | 34.120634 | 31.510465 |
| | BT-549 | 77.359686 | 84.872227 | 81.888099 | 81.640969 | 68.459454 | 71.57466 | 67.352519 | 65.28791 |
| | MDA-MB-468 | 66.116488 | 65.244702 | 58.786557 | 58.045178 | 30.731891 | 35.194234 | 34.563946 | 31.698793 |
| Basal OCR | MDA-MB-231 | 82.968107 | 85.647508 | 94.423427 | 92.085781 | 48.39327 | 49.599356 | 47.219131 | 43.810525 |
| | BT-549 | 188.55429 | 192.36109 | 192.09838 | 194.43096 | 126.7127 | 132.96802 | 121.75831 | 123.61615 |
| | MDA-MB-468 | 279.95764 | 251.43249 | 254.97411 | 248.81868 | 166.82875 | 154.55749 | 165.99434 | 144.48739 |

**Fig. 8D.**

| | Control | | | CT | | |
|---|---|---|---|---|---|---|
| Apoptotic cells | 1 | 2 | 3 | 1 | 2 | 3 |
| MDA-MB-231 | 6.25 | 6.65 | 7.89 | 28.79 | 31.49 | 29.85 |
| BT-549 | 16.23 | 6.73 | 12.25 | 56.9 | 58.7 | 48.75 |
| MDA-MB-468 | 5.67 | 14.19 | 6.53 | 76.26 | 80.93 | 77.68 |

**Fig. 9.**

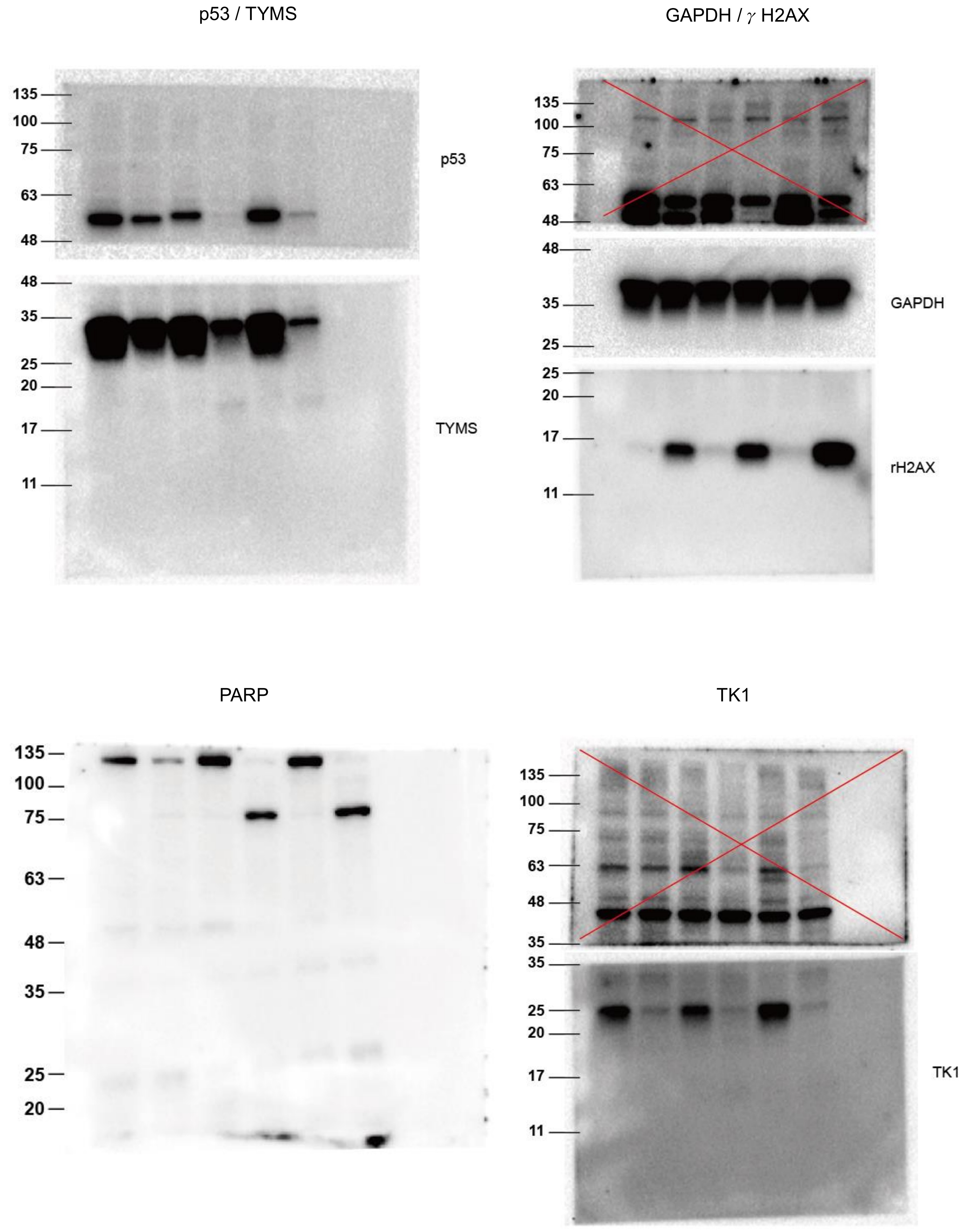

p53 / TYMS
135
100
75
63
48
p53
48
35
25
20
17
11
TYMS
GAPDH / γ H2AX
135
100
75
63
48
48
35
25
GAPDH
25
20
17
11
rH2AX
PARP
135
100
75
63
48
35
25
20
TK1
135
100
75
63
48
35
35
25
20
17
11
TK1
